# Racial disparities in inpatient palliative care consultation among frail older patients undergoing high-risk elective surgical procedures in the United States: a cross-sectional study of the national inpatient sample

**DOI:** 10.1093/haschl/qxad026

**Published:** 2023-07-13

**Authors:** Kyung Mi Kim, Ulrike Muench, John E Maki, Maria Yefimova, Anna Oh, Jeffrey K Jopling, Francesca Rinaldo, Nirav R Shah, Karleen Frances Giannitrapani, Michelle Y Williams, Karl A Lorenz

**Affiliations:** Office of Research Patient Care Services, Stanford Health Care, Menlo Park, CA 94025, United States; Clinical Excellence Research Center, School of Medicine, Stanford University, Palo Alto, CA 94304, United States; Department of Social and Behavioral Sciences, School of Nursing, University of California, San Francisco, San Francisco, CA 94143, United States; Department of Social and Behavioral Sciences, School of Nursing, University of California, San Francisco, San Francisco, CA 94143, United States; Philip R. Lee Institute for Health Policy Studies, School of Medicine, University of California, San Francisco, San Francisco, CA 94143, United States; Saint Francis Memorial Hospital, San Francisco, CA 94109, United States; Center for Nursing Excellence and Innovation, UCSF Health, San Francisco, CA 94143, United States; Department of Physiological Nursing, School of Nursing, University of California, San Francisco, San Francisco, CA 94143, United States; Office of Research Patient Care Services, Stanford Health Care, Menlo Park, CA 94025, United States; Clinical Excellence Research Center, School of Medicine, Stanford University, Palo Alto, CA 94304, United States; Department of Surgery, School of Medicine, Johns Hopkins University, Baltimore, MD 21287, United States; Clinical Excellence Research Center, School of Medicine, Stanford University, Palo Alto, CA 94304, United States; Clinical Excellence Research Center, School of Medicine, Stanford University, Palo Alto, CA 94304, United States; Center for Innovation to Implementation (Ci2i), Veterans Affairs Palo Alto Health Care System, US Department of Veterans Affairs, Palo Alto, CA 94304, United States; Quality Improvement Resource Center for Palliative Care, Stanford University, Stanford, CA 94305, United States; Primary Care and Population Health, School of Medicine, Stanford University, Stanford, CA 94305, United States; Office of Research Patient Care Services, Stanford Health Care, Menlo Park, CA 94025, United States; Primary Care and Population Health, School of Medicine, Stanford University, Stanford, CA 94305, United States; Center for Innovation to Implementation (Ci2i), Veterans Affairs Palo Alto Health Care System, US Department of Veterans Affairs, Palo Alto, CA 94304, United States; Quality Improvement Resource Center for Palliative Care, Stanford University, Stanford, CA 94305, United States; Primary Care and Population Health, School of Medicine, Stanford University, Stanford, CA 94305, United States

**Keywords:** palliative care consultation, racial/ethnic disparities, high-risk surgery, frail older patients

## Abstract

Surgical interventions are common among seriously ill older patients, with nearly one-third of older Americans facing surgery in their last year of life. Despite the potential benefits of palliative care among older surgical patients undergoing high-risk surgical procedures, palliative care in this population is underutilized and little is known about potential disparities by race/ethnicity and how frailty my affect such disparities. The aim of this study was to examine disparities in palliative care consultations by race/ethnicity and assess whether patients’ frailty moderated this association. Drawing on a retrospective cross-sectional study of inpatient surgical episodes using the National Inpatient Sample of the Healthcare Cost and Utilization Project from 2005 to 2019, we found that frail Black patients received palliative care consultations least often, with the largest between-group adjusted difference represented by Black–Asian/Pacific Islander frail patients of 1.6 percentage points, controlling for sociodemographic, comorbidities, hospital characteristics, procedure type, and year. No racial/ethnic difference in the receipt of palliative care consultations was observed among nonfrail patients. These findings suggest that, in order to improve racial/ethnic disparities in frail older patients undergoing high-risk surgical procedures, palliative care consultations should be included as the standard of care in clinical care guidelines.

## Introduction

Approximately 40% of all inpatient operations are performed on patients aged 65 years and older, and nearly one-third of older Americans face surgery in their last year of life.^1^ Compared with younger people, older adults are at a higher risk of postoperative mortality and complications due to decreased physiological reserve and diverse factors that contribute to frailty.^2,3^ Among older surgical patients, the prevalence of frailty is over 40%,^4^ and in-hospital mortality can be as high as 11%.^5^ With a 1-year mortality risk of 27.8%,^6^ frail older patients are likely to benefit from palliative care consultations when facing decisions about high-risk operations.

The benefits of palliative care consultations are becoming increasingly clear in surgical care.^7^ These consultations can help manage pain and symptoms, ascertain preferences to guide treatment (including life-sustaining care), provide emotional support, guide postoperative care, and help with discharge and transition plans for seriously ill patients and families.^8^ Notably, palliative care consultations are not confined to end-of-life situations. Such consultations also support patients with treatable, high-risk conditions, limited daily functionality, and burdensome symptoms, or aim to alleviate caregiver stress.^9^ Palliative care consultations do not imply limiting or withdrawing care,^10^ but could improve patients’ quality of life and reduce inappropriate, potentially burdensome care.^8^

Despite its potential benefits, palliative care consultations remain underutilized. Only 3.7% of surgical patients who underwent high-risk procedures received palliative care consultations within the period from 30 days before to 90 days after surgery.^11^ Even more concerning, the provision of palliative care is strikingly limited among Black and Hispanic/Latine patients who tend to be frailer^12^ and are at greater risk of mortality^13^ than White patients.^14,15^

Palliative care consultations, crucial for aligning care with the goals of patients and their families, are disproportionally underutilized in surgical patients compared with medical patients.^16^ Particularly at risk are frail older surgical patients who face a disproportionate burden of pain,^17^ lower survival rates, and other adverse postoperative outcomes,^18^ especially among certain racial and ethnic minorities.^19,20^ Therefore, research focusing on examining, understanding, and addressing racial and ethnic disparities in palliative care is of critical importance.^21^ It is also vital to understand potential racial/ethnic differences in the receipt of palliative care consultations for resource-distribution planning and targeted interventions to provide equal access and opportunities to quality care respecting the goals-of-care, dignity, and comfort of patients and families. However, little is known about whether disparities by race/ethnicity exist in the utilization of palliative care consultations,^22,23^ particularly among older patients undergoing high-risk surgery. We aimed to examine the association between palliative care consultations and race/ethnicity during hospital stays and whether frailty modified this association. We focused on elective surgical procedures, since in nonelective surgeries there may be little time to discuss patient preferences salient to palliative care consultations.^24^

## Data and methods

### Data source and study sample

**Figure 1.**
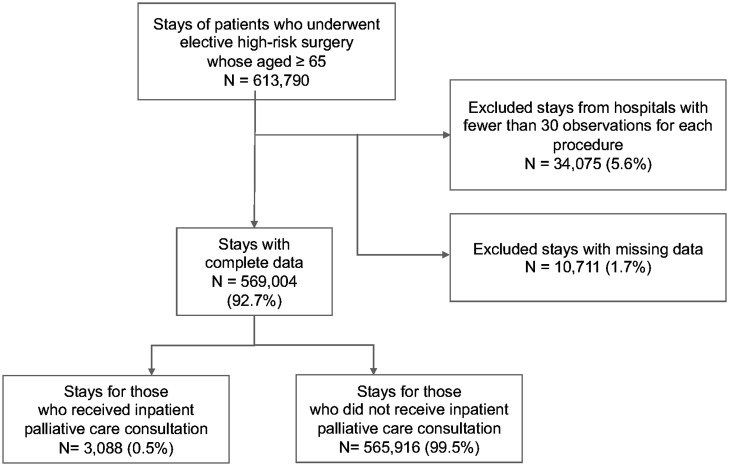
Flow diagram of sample selection. Source: Authors’ analysis of the Nationwide Inpatient Sample of the Healthcare Cost and Utilization Project data for 2005–2019.

We used the National Inpatient Sample (NIS) of the Healthcare Cost and Utilization Project (HCUP), the largest all-payer administrative database, to conduct a retrospective cross-sectional analysis of 569 004 inpatient surgical episodes, representing 3088 stays for those who received a palliative care consultation and 565 916 stays for those who did not receive a palliative care consultation. We included patients who were 65 years of age and older and admitted for elective high-risk surgical procedures, with the primary procedure having been performed between 2005 and 2019. We identified surgical risk using a list of high-risk surgeries developed by previous researchers.^25^ Originally, high-risk surgeries were identified using International Classification of Diseases, Ninth Revision (ICD-9), Clinical Modification (CM) codes. Because ICD codes were transitioned from ICD-9-CM to ICD-Tenth Revision CM and Procedure Coding System (PCS) codes in the fourth quarter of 2015, we converted the ICD-9-CM codes to the ICD-10-PCS codes aligned with our study period (2015 quarter 4–2019) using the equivalence mapping developed by the Centers for Medicare and Medicaid Services and the conversion files developed by the National Bureau of Economic Research.^26^ The full list of converted ICD-10-PCS codes is available in the Table S1. We excluded hospitals with fewer than 30 observations to avoid unstable estimates due to small sample sizes and observations with missing information on key study variables.^27,28^ Figure 1 shows the sample selection process.

### Outcomes and variables

The primary outcome was a binary variable indicating the receipt of a palliative care consultation during hospital stays for a high-risk surgical procedure. We identified receipt of a palliative care consultation using the ICD 9/10-CM codes (V66.7, Z515) from previous studies^15,29,30^ validated in Veterans Health Administration (VHA) data, the largest health care system in the United States.^31^

We used 5 race/ethnicity categories as available in the HCUP data: Asian and Pacific Islander, Black, Hispanic/Latine, Other, and White. Individuals who identified as Native American, multiracial, and other were grouped as “Other” because of the small sample size. The Hispanic/Latine category includes patients reported as either Hispanic or Latino. In the HCUP, Hispanic/Latine ethnicity is prioritized over race. This means that, if a person reports their ethnicity as Hispanic/Latine, they are defined as Hispanic/Latine regardless of reported race.

We selected known or hypothesized characteristics associated with inpatient palliative care consultation as covariates a priori. Patient characteristics included the following: frailty (frail/nonfrail) measured as the presence of at least 1 of 10 frailty diagnoses as per Johns Hopkins Adjusted Clinical Groups (ACG) frailty score (Table S2),^32^ sex (female/male), age, median household income for patient's zip code by quartile, and 26 indicators from the modified Elixhauser comorbidity index. We excluded dementia and weight loss from the comorbidity index because they were accounted for in the ACG frailty index.^33^ Fluid and electrolyte disorders were also excluded from the comorbidity index because they were not available after 2018. Hospital characteristics included bed size (small/medium/large), ownership (public/private), location and teaching status (rural teaching and nonteaching/urban nonteaching/urban teaching), and log-transformed surgical volume. We also included fixed effects for procedure type (general, neurology, otorhinolaryngology, cardiac, thoracic, vascular, orthopedic, urology, gynecology, transplant), hospital, and year to account for secular trends. We used clinical classification software developed by the HCUP to categorize the surgeries into specialty groups.^34^ Two of the authors (K.K. and J.E.M.) with clinical expertise in surgery reviewed these categories to ensure they aligned with clinical practice, and we created a 10-category surgical specialty variable, as indicated above.

### Statistical analyses

We summarized patient characteristics descriptively based on whether a patient received a palliative care consultation during their hospital stays. To compare the groups of patients with and without the palliative care consultations, we used standardized mean differences because they are less sensitive to large sample sizes than tests of significance.^35^ We also examined patient characteristics by race/ethnicity.

To assess the association between the receipt of a palliative care consultation and race/ethnicity, we used linear probability models and adjusted for all covariates listed above. Linear probability models provide unbiased, consistent estimation with fixed effects,^36,37^ and they outperform logistic regression, particularly when the binary outcome is of low prevalence or rare.^38^ To evaluate whether frailty moderates the association between race/ethnicity and the receipt of a palliative care consultation, we included an interaction term of race/ethnicity and frailty in the fully adjusted model.

A series of sensitivity analyses were conducted to assess the robustness of our results. These included repeating the main analyses using logistic regression and using a conditional multiple imputation by chained equation to address concerns about a moderate amount of missing race/ethnicity data in the HCUP.^39^

Survey weights were applied to all analyses to obtain nationally representative estimates and to account for the HCUP's complex survey designs. Analyses were performed using Stata MP version 17.0 (StataCorp LLC) between November 2022 and June 2023. All *P* values were from 2-sided tests, and results were deemed statistically significant at the false discovery rate (FDR) adjusted *P*-value < .05 to address the multiple comparisons between different racial/ethnic groups.^40,41^ We used a 2-stage approach to control the FDR to adjust *P*-values^42^ due to the greater statistical power of FDR control, especially when testing a large number of hypotheses (Appendix Methods).^40,41^ Patient consent was waived because the study comprised secondary analysis of archival data prior to the analysis. The study was exempted by the Stanford University Institutional Review Board. The study followed the Strengthening the Reporting of Observational Studies in Epidemiology (STROBE) reporting guideline for cross-sectional studies.

### Limitations

This study used administrative data, which rely on hospitals accurately reporting palliative care consultations and identifying them using ICD-9/10-CM codes. Although we used the ICD-9/10-CM codes, validated in prior research for identifying specialist palliative care in the VHA, surgical teams’ discussion about goals of care may not be coded in non-VHA administrative data. This likely resulted in an underestimation of palliative care consultations in these data. Nonetheless, our findings aligned with those from the VHA, known for its longstanding practice of capturing palliative care consultations data.^11,43^ Observed associations with race/ethnicity and palliative care consultations may be underestimated due to undetected palliative care consultations prior to admission or after discharge. Our results pertain specifically to US inpatient surgical care and are not generalizable to other contexts. While the HCUP, a large-scale data set, has been widely utilized in health care research, it is known to have a moderate amount of missing race and ethnicity data, which may bias the estimates.^39^ To address these concerns, we used a conditional multiple imputation by chained equation and obtained consistent results. Further limitations include the aggregated nature of race/ethnicity data reported in the HCUP, which hampers our ability to scrutinize potential differences among more granular racial and ethnic groups. The heterogeneity within these aggregated race/ethnic groups is well documented, and the problems associated with the lack of detailed racial and ethnic data, limiting the delivery of targeted interventions, are increasingly recognized.^44^ Systematic disaggregation of racial and ethnic data is critical during all stages of research: data collection, reporting, analysis, and dissemination.^44^ Finally, our results may be subject to random error due to the large sample size and the low prevalence of palliative care consultations.^45,46^ To address this issue, we reported statistical significance at the FDR-adjusted *P*-value and adhered to the reporting standards for low-prevalence health care outcomes as established by the National Center for Health Statistics.^47,48^

## Results

Of 569 004 surgical episodes, the majority of episodes were from White patients (83.2%), followed by patients identifying as Black (6.4%), Hispanic/Latine (5.3%), Asian/Pacific Islander (2.7%), and other (2.1%).

A summary of sociodemographic characteristics in Table 1 shows that racial/ethnic differences were the largest for income. A proportion of patients residing in the zip code of the 75–100th-percentile median household income was the largest among Asian/Pacific Islander patients (46.1%) and the lowest among Black patients (12.4%). Differences examined using standardized mean differences are presented in Figure S1. The differences between people identifying as Asian/Pacific Islander and Black were the largest in the income quartile, whereas the differences between people identifying as Asian/Pacific Islander and White were the largest for the location and teaching status of hospitals where they received care.

**Table 1.** Characteristics of hospital stays for patients aged 65 years and older admitted for elective high-risk elective surgical procedures by race/ethnicity.

	Asian/Pacific Islander (n = 11 253)		Black (n = 32 961)		Hispanic/Latine (n = 27 473)		Other (n = 14 376)		White (n = 425 102)	
	n	%	n	%	n	%	n	%	n	%
Sex										
Male	6741	59.9	16 447	49.9	15 788	57.5	8661	60.3	252 697	59.5
Female	4509	40.1	16 505	50.1	11 679	42.5	5711	39.7	172 329	40.5
Age, mean (SD), y	73.3	6.2	72.4	5.9	73.0	6.1	73.2	6.0	73.7	6.3
Frailty										
Frail	1263	11.2	4502	13.7	2846	10.4	1464	10.2	40 848	9.6
Inpatient mortality	235	2.1	667	2.0	541	2.0	272	1.9	8056	1.9
Median income quartile										
0–25th percentile	1256	11.3	15 889	49.1	9288	34.7	3338	23.8	88 669	21.2
26–50th percentile	1876	16.9	7097	21.9	6403	23.9	3134	22.4	112 893	27
51–75th percentile	2840	25.6	5361	16.6	6333	23.7	3391	24.2	110 750	26.5
76–100th percentile	5110	46.1	4013	12.4	4732	17.7	4146	29.6	105 895	25.3
Type of insurance										
Medicare	8664	77.1	27 634	84	22 345	81.5	11 641	81.2	373 287	87.9
Medicaid	730	6.5	531	1.6	1062	3.9	496	3.5	1688	0.4
Private	1626	14.5	4117	12.5	3409	12.4	1893	13.2	44 144	10.4
Self-pay	122	1.1	166	0.5	286	1.0	167	1.2	1447	0.3
Others										
Comorbidity										
0	1327	11.9	2700	8.3	2811	10.3	1648	11.5	48 434	11.4
1	2789	24.8	6692	20.3	5976	21.8	3350	23.3	102 882	24.2
2	3134	27.8	8829	26.7	7302	26.6	3952	27.5	114 587	27.0
≥3	4003	35.5	14 740	44.7	11 384	41.4	5426	37.7	159 199	37.4
Bed size										
Small	1279	11.4	3501	10.7	3311	12.1	1790	12.5	45 919	10.8
Medium	2367	21.1	8139	24.8	6559	23.9	2926	20.4	99 125	23.4
Large	7589	67.5	21 209	64.6	17 571	64.0	9597	67.1	278 773	65.8
Location and teaching status										
Rural	126	1.1	1095	3.3	364	1.3	591	4.1	23 379	5.5
Urban, non-teaching	2850	25.4	6655	20.3	8227	30.0	3837	26.8	120 521	28.4
Urban, teaching	8259	73.5	25 099	76.4	18 850	68.7	9885	69.1	279 917	66.0
Ownership										
Government	1668	14.9	4792	14.6	3914	14.3	2335	16.3	54 080	12.8
Private	9564	85.1	28 047	85.4	23 512	85.7	11 951	83.7	368 948	87.2

Source: Authors’ analysis of the Nationwide Inpatient Sample of the Healthcare Cost and Utilization Project data for 2005–2019. The numbers of observations are unweighted raw numbers. Percentages are survey weighted. Rows may not add up to 100% due to rounding. The individuals who identified as Native American, multiracial, and other were combined into a single convenience category (“Other”) because of the small sample size.

Compared with the cohort who did not receive palliative care consultations (99.5%) during their hospital stay, patients who did receive such consultations (0.5%) tended to be frail (36.1% vs 9.7%), female (47.8% vs 41.2%), older (mean age: 76.3 vs 73.6 y), and covered by Medicare (90.1% vs 86.8%), and had a higher prevalence of congestive heart failure (12.2% vs 3.3%), coagulopathy (21.0% vs 10.0%), metastatic cancer (16.0% vs 6.3%), neurologic disorders such as dementia (8.8% vs 3.7%), paralysis (5.8% vs 1.2%), or renal failure (18.8% vs 10.8%) (Table 2). In addition, patients who underwent otorhinolaryngology (3.7% vs 0.7%) or general (33.8% vs 27.5%) surgeries or those in urban teaching (74.9% vs 67.1%) or private (90.4% vs 86.4%) hospitals were more likely to receive palliative care consultations compared with those who did not. While 1.6% of patients who did not receive palliative care consultations died during hospitalization, in-hospital mortality increased to 61.1% among those who received such consultations. Black patients (13.7%) were the most frail, Asian/Pacific Islanders (11.2%) were the second-most frail, and White patients (9.6%) were the least frail.

**Table 2.** Characteristics of hospital stays for patients who received inpatient palliative care consultation and those who did not receive inpatient palliative care consultation.

	Inpatient palliative care consultation (n = 569 004)				Standardized difference in means
	No (n = 569 916)		Yes (n = 3088)		
	n	%	n	%	
**Patient characteristics**					
Race/ethnicity					
Asian/Pacific Islander	11 176	2.2	77	2.7	0.04
Black	32 762	6.4	199	6.9	0.02
Hispanic/Latine	27 320	5.4	153	5.3	0.00
Other	14 317	2.8	59	2.1	−0.05
White	422 703	83.2	2399	83.0	−0.01
Sex					
Male	331 919	58.8	1613	52.2	−0.14
Female	233 387	41.2	1474	47.8	0.14
Age, mean (SD), y	73.6	6.2	76.3	6.9	0.42
Frailty					
Nonfrail	511 099	90.3	1976	63.9	−0.66
Frail	54 817	9.7	1112	36.1	0.66
Inpatient mortality	8614	1.6	1799	61.1	1.67
Median income quartile					
0–25th percentile	129 304	23.2	744	24.3	0.03
26–50th percentile	148 884	26.8	847	27.9	−0.02
51–75th percentile	143 998	25.9	767	25.2	−0.04
76–100th percentile	133 935	24.1	687	22.6	−0.07
Type of insurance					
Medicare	466 129	86.8	2649	90.1	0.10
Medicaid	4555	0.9	31	1.1	0.02
Private	58 876	11.0	214	7.3	−0.13
Self-pay	2174	0.4	16	0.6	0.02
Others	5413	1.0	29	1.0	0.00
Elixhauser comorbidity index					
AIDS	238	0.0	<5	0.0	0.00
Alcohol abuse	8241	1.5	47	1.5	0.01
Deficiency anemias	77 936	13.7	504	16.1	0.07
Arthropathies	14 087	2.5	80	2.6	0.01
Chronic blood loss anemia	7886	1.4	67	2.1	0.05
Congestive heart failure	18 677	3.3	382	12.2	0.34
Chronic pulmonary disease	118 209	20.9	749	24.3	0.08
Coagulopathy	56 732	10.0	647	21.0	0.30
Depression	37 342	6.6	203	6.6	−0.01
Diabetes without chronic complications	119 997	21.1	451	14.5	−0.18
Diabetes with chronic complications	41 342	7.4	237	7.7	0.01
Drug abuse	1550	0.3	10	0.3	0.01
Hypertension	357 756	63.1	1567	50.6	−0.27
Hypothyroidism	69 770	12.4	316	10.2	−0.07
Liver disease	8252	1.5	98	3.2	0.11
Lymphoma	3416	0.6	30	1.0	0.04
Metastatic cancer	35 813	6.3	496	16.0	0.31
Neurological disorders	20 880	3.7	273	8.8	0.21
Obesity	72 932	13.0	253	8.2	−0.16
Paralysis	6523	1.2	178	5.8	0.26
Peripheral vascular disease	83 316	14.7	580	18.7	0.11
Psychoses	6908	1.2	43	1.4	0.01
Pulmonary circulation disease	4729	0.8	115	3.7	0.19
Renal failure	60 953	10.8	583	18.8	0.23
Solid tumor without metastasis	13 907	2.5	101	3.3	0.04
Peptic ulcer disease excluding bleeding	947	0.2	21	0.7	0.08
Valvular disease	14 569	2.6	134	4.3	0.10
Weight loss	24 384	4.3	793	25.6	0.63
Type of procedure					
Neurology	2829	0.5	26	0.8	0.04
Otorhinolaryngology	3856	0.7	113	3.7	0.21
Thoracic	16 205	2.9	111	3.6	0.03
Cardiac	242 750	42.9	1197	38.7	−0.09
Vascular	38 578	6.8	196	6.3	−0.02
General	156 879	27.5	1046	33.8	0.14
Urology	83 459	14.8	243	7.9	−0.22
Gynecology	20 177	3.6	138	4.5	0.05
Orthopedics	446	0.1	11	0.4	0.06
Transplant	737	0.1	7	0.2	0.02
**Hospital characteristics**					
Bed size					
Small	61 595	10.8	275	8.9	−0.07
Medium	128 892	23.0	690	22.5	−0.01
Large	373 808	66.2	2114	68.5	0.05
Location and teaching status					
Rural	30 312	5.2	125	4.0	−0.04
Urban, non-teaching	157 462	27.7	660	21.1	−0.16
Urban, teaching	376 521	67.1	2294	74.9	0.17
Ownership					
Government	77 891	13.6	297	9.6	−0.12
Private	484 901	86.4	2781	90.4	0.12

Source: Authors’ analysis of the Nationwide Inpatient Sample of the Healthcare Cost and Utilization Project data for 2005–2019. The numbers of observations are unweighted raw numbers. Percentages are survey weighted. Rows may not add up to 100% due to rounding. The individuals who identified as Native American, multiracial, and other were combined into a single convenience category (“Other”) because of the small sample size. Standardized mean differences between 0.2 and less than 0.5, 0.5 and 0.8, and greater than 0.8 are considered small, medium, and large, respectively.

**Figure 2.**
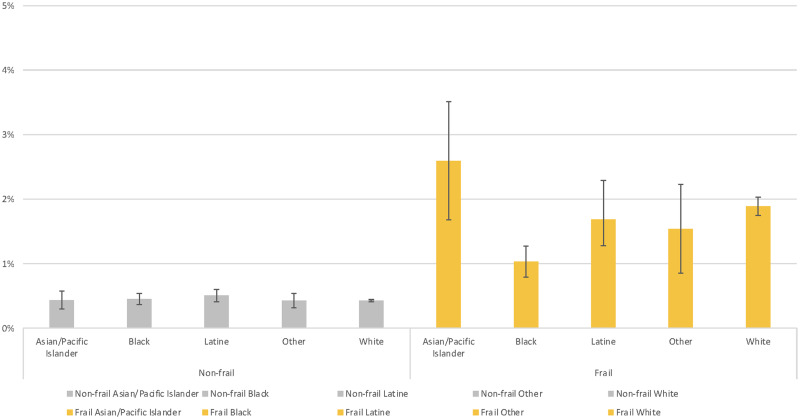
Covariate-adjusted estimates for receiving palliative care consultations during hospital stays by race/ethnicity and frailty. Models adjusted for frailty, interaction between race/ethnicity and frailty, sociodemographic characteristics (sex, age, median household income for the patient's zip code), comorbidities, hospital characteristics (bed size, location and teaching status, ownership), and fixed effects for procedure type (general, neurology, otorhinolaryngology, cardiac, thoracic, vascular, orthopedics, urology, gynecology, transplant), hospital, and year. Source: Authors’ analysis of the Nationwide Inpatient Sample of the Healthcare Cost and Utilization Project data for 2005–2019.

From the covariate-adjusted linear probability model, estimates for receiving palliative care consultations during hospital stays indicate that, of those who were frail, Black patients were least likely to receive palliative care consultations (Figure 2). Among frail patients, the largest difference in receipt of palliative care consultations was between Black and Asian/Pacific Islander patients (−1.6 percentage points; 95% CI: −2.5 to −0.6; *P* = .021). The second largest difference was between Black and White patients (−0.9 percentage points; 95% CI: −1.2 to −0.5; *P* = .021). No racial/ethnic difference in the receipt of palliative care consultations during hospital stays was observed among nonfrail patients.

**Figure 3.**
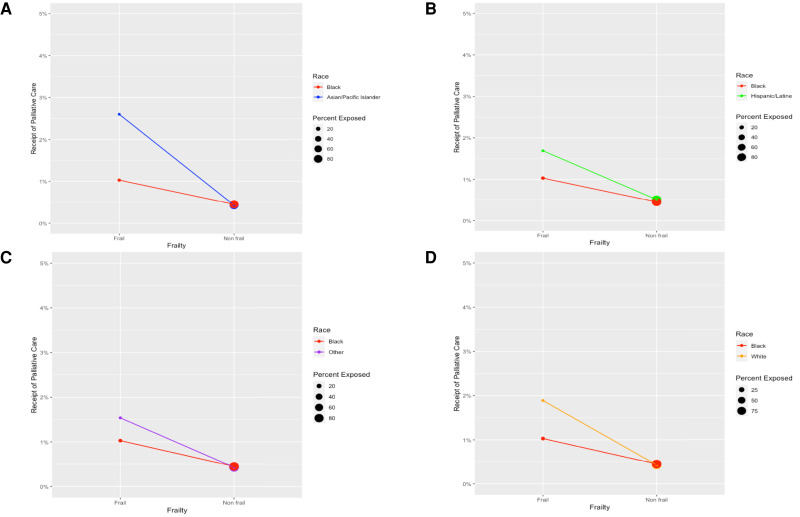
A–D: Interaction effect present between race/ethnicity and frailty: covariate-adjusted estimates for receiving palliative care. Models adjusted for frailty, interaction between race/ethnicity and frailty, sociodemographic characteristics (sex, age, median household income for the patient's zip code), comorbidities, hospital characteristics (bed size, location and teaching status, ownership), and fixed effects for procedure type (general, neurology, otorhinolaryngology, cardiac, thoracic, vascular, orthopedics, urology, gynecology, transplant), hospital, and year. The circles represent the proportion of each racial/ethnic group at each level of frailty (ie, frail vs nonfrail). Larger circles indicate a greater proportion. For example, the circle representing the frailty of Black patients is larger than that for Asian/Pacific Islanders, which indicates that a greater proportion of Black patients are exposed to frailty. Source: Authors’ analysis of the Nationwide Inpatient Sample of the Healthcare Cost and Utilization Project data for 2005–2019.

**Figure 4.**
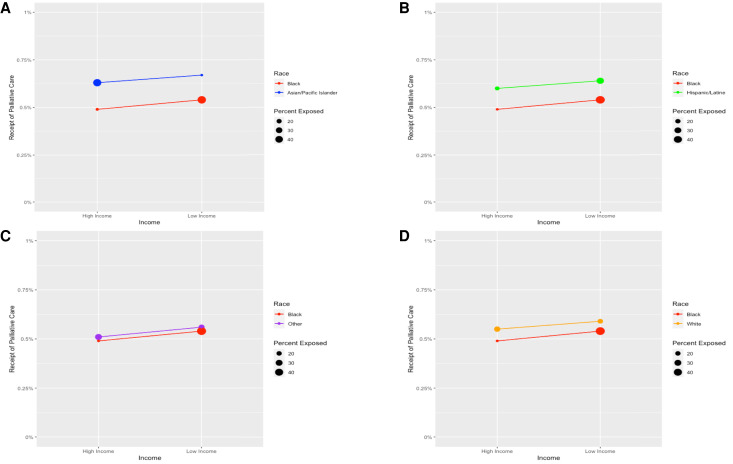
A–D: No interaction effect present between race/ethnicity and income: covariate-adjusted estimates for receiving palliative care. Models adjusted for frailty, interaction between race/ethnicity and frailty, sociodemographic characteristics (sex, age, median household income for the patient's zip code), comorbidities, hospital characteristics (bed size, location and teaching status, ownership), and fixed effects for procedure type (general, neuro logy, otorhinolaryngology, cardiac, thoracic, vascular, orthopedics, urology, gynecology, transplant), hospital, and year. The circles represent the proportion of each racial/ethnic group at each level of income (ie, low vs high). Larger circles indicate a greater proportion. For example, the circle representing low-income Black patients is larger than that for Asian/Pacific Islanders, which indicates a greater proportion of low-income Black patients. The figure presents racial/ethnic disparities in income, but income does not have an interaction effect on the receipt of palliative care. Source: Authors’ analysis of the Nationwide Inpatient Sample of the Healthcare Cost and Utilization Project data for 2005–2019.

Figure 3 illustrates the interaction effect between race/ethnicity and frailty. The difference in slope indicates that the receipt of palliative care consultations associated with frailty among Black patients was relatively small compared with the increased probability of receiving palliative care consultations observed among Asian/Pacific Islander, White, and Hispanic/Latine patients, despite racial/ethnic disparities in frailty. Meanwhile, income, showing the largest racial/ethnic difference among socioeconomic factors, was not attributable to the racial/ethnic disparity in the receipt of palliative care consultations during hospital stays (Figure 4).

A sensitivity analysis using logistic regression produced results almost identical to those of the linear probability model, indicating racial disparities in palliative care consultations during hospital stays (Table S3). We also imputed missing race/ethnicity variables using a conditional multiple imputation by chained equation and found consistent results.

## Discussion

In a nationally representative inpatient sample, the rate of palliative care consultations during hospital stays for all patients undergoing high-risk surgeries was low, at less than 1%. Despite an already-low overall palliative care consultation rate, Black frail patients were the least likely to receive palliative care consultations than any other racial/ethnic group of frail patients. Black frail patients were offered palliative care consultations only one-third and one-half of the rates of such consultations among similarly frail Asian/Pacific Islander and White patients, respectively.

Our findings align with other studies of palliative care among seriously ill adults and suggest that multiple factors faced by this marginalized population may similarly impact the receipt of palliative care consultations: family or neighborhood socioeconomic status, social group experiences (eg, culturally specific experiences differing by racial/ethnic or socioeconomic groups),^49^ or structural racism (eg, provider and institutional conscious and unconscious racist cultural beliefs and practices, systematic underinvestment in certain neighborhoods, inadequate access to pain and symptom management, and a lack of documentation of end-of-life wishes to be honored).^50,51^

Racial disparities in surgical care are generally rooted at the intersections of multiple factors, including higher rates of comorbidities, delays in seeking care, receipt of care at low-quality hospitals, and inadequate access to care.^52^ Our findings suggest that frail, older Black patients also have inadequate access to palliative care, which may limit their end-of-life care choices and impede their access to high-quality care including their family members and other caregivers.^8^

It is troubling both that the disparity we documented is so pervasive among medical, as well as apparently surgical, patients but also that so little intervention work has focused on improving palliative care outcomes among Black, Hispanic/Latine, and other marginalized populations. A recent extensive and rigorous systematic review found only 5 randomized controlled studies that attempted to directly address such disparities.^53^ An important gap but promising trend in health services research is the adoption of co-design. This approach deeply involves vulnerable individuals, allowing them to identify challenges and craft solutions from their unique perspective, instead of relying exclusively on insights from experts or providers.^23,54^ Certainly, support for research, conducted by racially/ethnically diverse investigators, focused on innovations to address disparities in palliative care among marginalized populations is badly needed.^54^ Explicitly acknowledging the impact of structural racism is also important, in addition to an individual's conscious and unconscious biases and stereotyping, as an uppermost factor on disparities in palliative care.^23,51^

Our study has 2 important implications. First, the provider's initiation of inpatient palliative care might be rooted in their assessment of the patient's frailty.^7^ Considering that frail patients received inpatient palliative care about 4 times more often than nonfrail patients (36.1% vs 9.7%), providing a tool that enables clinicians to assess frailty easily might abet increasing the use of palliative care consultations. Although there are emerging tools to assess frailty and surgical risk, such as the Risk Analysis Index,^55^ if these tools are not well incorporated into the existing workflow or clinical guidelines, assessment of frailty risk will rely on subjective perceptions, or it might not be performed at all. Opportunities likely exist to improve the use of palliative care among frail surgical patients because a palliative care consultation was not offered to 98% of frail patients during hospitalization. Second, despite the positive association between frailty and the receipt of palliative care consultations in all racial/ethnic groups, frailty had the smallest contribution to Black patients’ receipt of palliative care consultations. Clinicians may discuss prognostic uncertainty, life expectancy, and all possible care options less frequently with frail Black patients^56^ than they do with other racial/ethnic groups of patients. Clinicians’ implicit bias, such as a tendency to falsely assume that non-White patients, especially Black and Hispanic/Latine patients, can tolerate more pain or prefer to have more aggressive end-of-life care, might be associated with decreased clinician engagement in such discussions.^57,58^ Further, Black patients may be concerned that this type of care could compromise their access to treatment, a belief possibly grounded in mistrust in the health care system exacerbated by historical and extant racism.^22,51,59,60^

Despite the main takeaway from our study being the disparities in the use of palliative care consultations among frail Black patients, it is important to note that the rate of palliative care consultations during hospital stays for patients undergoing high-risk surgeries is strikingly low at less than 1% across all racial/ethnic groups. In contrast, higher utilization rates were reported in the VHA system, where the documentation of such consultations is mandatory.^43^ Although clinicians generally agree on the importance of palliative care, many surgeons report receiving minimal to no palliative care education, feel uncomfortable introducing and talking with their patients about palliative care, and fear confrontations from patients and their families or caregivers.^61^ Providing appropriate education could empower clinicians to initiate these consultations more often, potentially enhancing care for frail older surgical patients undergoing high-risk procedures by honoring the preferences and goals of care and offering early/timely referral to a palliative care consultation, thereby improving patients’ quality of life, symptom management, end-of-life care, and survival.^62,63^ Surgical care could potentially learn from other specialties, such as oncology, where over 30% of patients receive palliative care consultations.^64^ Such efforts would benefit the health care system, patients, and their families and caregivers, and may mitigate the immense costs of care while expanding options and avoiding harm to patients.

The low utilization of palliative care among surgical patients, especially Black patients, may be linked to a lack of diversity in the palliative care workforce. This workforce gap in surgical specialties, including surgeons and anesthesiologists, is particularly concerning.^65^ With only 75 surgeons in the United States who specialize in hospice and palliative care,^66^ there is likely a shortage of racial and ethnic representation among clinicians. This deficit potentially hinders the capacity to serve the diverse needs of surgical patients in palliative care. While diversifying the surgical workforce may be a long-term goal, policy efforts need to be initiated.

Our results revealed that palliative care consultations are vastly underutilized in older adults undergoing high-risk surgeries. Given the procedural risks in these frail older adults and the associated risk of a poor prognosis, such consultations should be integrated into the standard care for frail older patients undergoing high-risk surgical procedures. These consultations should span the full spectrum of routine perioperative care,^10^ from discussing care goals preoperatively to aiding postoperative recovery in case of severe complications. Furthermore, access to these consultations should be equitable, available to all seriously ill patients and their families. Despite the current infrequent use of palliative care consultations, our study has uncovered disparities in their use. If the utilization of these consultations were to increase, these disparities might persist or even widen. Therefore, documenting these disparities is a crucial first step toward achieving equitable access to palliative care for frail older patients undergoing high-risk surgical procedures.

## Conclusion

Older Black frail patients undergoing surgery were less likely to receive a palliative care consultation during their hospitalization than other frail racial/ethnic groups. Our findings suggest that proactive interventions addressing frailty alone would not be sufficient to ameliorate the racial/ethnic disparity in palliative care for surgical patients. Our study underscores the need to continually assess disparities stemming from health care system factors and clinician discrimination (eg, clinician unconscious bias, stereotyping).^67^ Furthermore, systematic efforts are warranted to improve access to palliative care for all patients undergoing high-risk elective procedures, particularly for frail Black patients. This would enable patients to make choices that better align with their goals-of-care, including expanded end-of-life care choices in surgical settings.

## Supplementary Material

qxad026_Supplementary_Data
