# Supplementary material for: Racial disparities in inpatient palliative care consultation among frail older patients undergoing high-risk elective surgical procedures in the United States: a cross-sectional study of the national inpatient sample
Source: Health Aff Sch. 2023 Jul 13;1(2):qxad026. doi: 10.1093/haschl/qxad026 (PMC10986263; doi:10.1093/haschl/qxad026)
Supplement: qxad026_Supplementary_Data [file qxad026_Supplementary_Data.zip › Kim_Appendix_Revision v2_Clean.docx]

**Appendix Materials**

**Appendix Method.** Accounting for multiple comparisons: Unadjusted p-value vs. false discovery rate-adjusted p-value

**Appendix Table 1.** ICD-9-CM and ICD-10-PCS codes to identify elective high-risk surgical procedures

**Appendix Table 2.** Frailty measures from the 10-Item Johns Hopkins ACG with associated conditions and related ICD-9/10-CM codes

**Appendix Table 3.** Estimates of the association between race and ethnicity and the receipt of inpatient palliative care consultation from the Logistic Regression Model

**Appendix Figure 1.** Boxplots of standardized mean differences of covariates between racial/ethnic groups

**Appendix Method.** Accounting for multiple comparisons: Unadjusted p-value vs. false discovery rate-adjusted p-value

To account for multiple comparisons, we employed a two-stage approach for controlling the false discovery rate (FDR).^1^ We chose this method over controlling the familywise error rate, such as the Bonferroni correction, due to the greater statistical power of FDR control when testing a large number of hypotheses.^2,3^ Our multiple comparison correction yielded results largely consistent with the unadjusted p-values. We noted statistically significant differences between Black and Asian/Pacific Islander patients, as well as between Black and White patients. However, the discrepancy between Black and Hispanic/Latine patients was deemed statistically non-significant after p-value adjustment. We reported results deemed statistically significant at the FDR-adjusted p-value < 0.05. The table below compares the unadjusted p-value to the FDR-adjusted p-value (* denotes statistically significant differences at FDR-adjusted P < 0.05).

|  | Covariate-Adjusted Probabilities of receiving inpatient palliative care consultation (%)  from  Linear Probability Model (95% CI) | p-value | False discovery rate adjusted p value  (sharpened q-value) |
| --- | --- | --- | --- |
| **Difference among non-frail patients** |  |  |  |
| Black – Asian/Pacific Islander | 0.02 (-0.26, 0.30) | 0.830 | 1 |
| Black – Hispanic/Latine | -0.05 (-0.27, 0.16) | 0.427 | 0.799 |
| Black – Other | 0.03 (-0.21, 0.26) | 0.704 | 1 |
| Black – White | 0.03 (-0.12, 0.18) | 0.552 | 1 |
| Asian/Pacific Islander – Hispanic/Latine | -0.07 (-0.35, 0.21) | 0.411 | 0.799 |
| Asian/Pacific Islander – Other | 0.01 (-0.28, 0.30) | 0.917 | 1 |
| Asian/Pacific Islander – White | 0.01 (-0.23, 0.24) | 0.902 | 1 |
| Hispanic/Latine – Other | 0.08 (-0.16, 0.32) | 0.283 | 0.692 |
| Hispanic/Latine – White | 0.08 (-0.09, 0.25) | 0.121 | 0.510 |
| Other – White | -0.00 (-0.19, 0.19) | 0.994 | 1 |
| **Difference among frail patient** |  |  |  |
| Black – Asian/Pacific Islander* | -1.56 (-3.20, 0.07) | 0.002 | 0.021 |
| Black – Hispanic/Latine | -0.65 (-1.67, 0.37) | 0.037 | 0.286 |
| Black – Other | -0.51 (-1.80, 0.79) | 0.201 | 0.605 |
| Black – White* | -0.89 (-1.46, -0.26) | <0.001 | 0.021 |
| Asian/Pacific Islander – Hispanic/Latine | 0.91 (-0.82, 2.64) | 0.086 | 0.414 |
| Asian/Pacific Islander – Other | 1.06 (-0.88, 2.99) | 0.076 | 0.414 |
| Asian/Pacific Islander – White | 0.70 (-0.85, 2.26) | 0.139 | 0.510 |
| Hispanic/Latine – Other | 0.15 (-1.21, 1.50) | 0.726 | 1 |
| Hispanic/Latine – White | -0.21 (-1.08, 0.67) | 0.444 | 0.799 |
| Other – White | -0.35 (-1.52, 0.82) | 0.328 | 0.744 |

**References**

1. Benjamini Y, Krieger AM, Yekutieli D. Adaptive linear step-up procedures that control the false discovery rate. *Biometrika*. 2006;93(3):491-507.

2. Anderson ML. Multiple inference and gender differences in the effects of early intervention: a reevaluation of the abecedarian, perry preschool, and early training projects. *J Am Stat Assoc*. 2008;103(484):1481-1495.

3. Lee S, Lee DK. What is the proper way to apply the multiple comparison test? *Korean J Anesthesiol*. 2018;73(6):572.

**Appendix Table 1.** ICD-9-CM and ICD-10-PCS codes to identify elective high-risk surgical procedures

| **Specialty** | **Modified CCS description** | **Name of procedure** | **ICD9** | **ICD10 PCS** |
| --- | --- | --- | --- | --- |
| Neuro | Incision and excision of CNS | other craniotomy | 1.24 | \| 00J00ZZ,00W00JZ,00W00KZ,0N800ZZ,0N803ZZ \| \| \| \| \| \| --- \| --- \| --- \| --- \| --- \| \| 0N804ZZ,0NC10ZZ,0NC13ZZ,0NC14ZZ,0NC20ZZ \| \| \| \| \| \| 0NC23ZZ,0NC24ZZ,0NC30ZZ,0NC33ZZ,0NC34ZZ \| \| \| \| \| \| 0NC40ZZ,0NC43ZZ,0NC44ZZ,0NC50ZZ,0NC53ZZ \| \| \| \| \| \| 0NC54ZZ,0NC60ZZ,0NC63ZZ,0NC64ZZ,0NC70ZZ \| \| \| \| \| \| 0NC73ZZ,0NC74ZZ,0NC80ZZ,0NC83ZZ,0NC84ZZ \| \| \| \| \| \| 0NH00MZ,0NH03MZ,0NH04MZ,0NP000Z,0NP004Z \| \| \| \| \| \| 0NP005Z,0NP007Z,0NP00KZ,0NP00SZ,0NP030Z \| \| \| \| \| \| 0NP034Z,0NP037Z,0NP03KZ,0NP03SZ,0NP040Z \| \| \| \| \| \| 0NP044Z,0NP047Z,0NP04KZ,0NP04SZ,0NP0X4Z \| \| \| \| \| \| 0NP0XSZ,0NW000Z,0NW004Z,0NW005Z,0NW007Z \| \| \| \| \| \| 0NW00JZ,0NW00KZ,0NW00MZ,0NW00SZ,0NW030Z \| \| \| \| \| \| 0NW034Z,0NW035Z,0NW037Z,0NW03JZ,0NW03KZ \| \| \| \| \| \| 0NW03MZ,0NW03SZ,0NW040Z,0NW044Z,0NW045Z \| \| \| \| \| \| 0NW047Z,0NW04JZ,0NW04KZ,0NW04MZ,0NW04SZ \| \| \| \| \| \| 0W9100Z,0W910ZZ,0WC10ZZ,0WC13ZZ,0WC14ZZ \| \| \| \| \| \| 0WH10YZ,0WH13YZ,0WH14YZ,0WJ10ZZ,0WP100Z \| \| \| \| \| \| 0WP101Z,0WP10JZ,0WP10YZ,0WP130Z,0WP131Z \| \| \| \| \| \| 0WP13JZ,0WP13YZ,0WP140Z,0WP141Z,0WP14JZ \| \| \| \| \| \| 0WP14YZ,0WW100Z,0WW101Z,0WW103Z,0WW10JZ \| \| \| \| \| \| 0WW10YZ,0WW130Z,0WW131Z,0WW133Z,0WW13JZ \| \| \| \| \| \| 0WW13YZ,0WW140Z,0WW141Z,0WW143Z,0WW14JZ \| \| \| \| \| \| 0WW14YZ \|  \|  \|  \| |
|  |  | other craniectomy | 1.25 | \| 0N500ZZ,0N503ZZ,0N504ZZ,0NB00ZZ,0NB03ZZ \| \| --- \| \| 0NB04ZZ,0NT10ZZ,0NT20ZZ,0NT30ZZ,0NT40ZZ \| \| 0NT50ZZ,0NT60ZZ,0NT70ZZ,0,0 \| |
|  |  | incise cerebral meninges | 1.31 | 009100Z,00910ZZ,00C10ZZ,00C13ZZ,00C14ZZ |
|  |  | other brain incision | 1.39 | \| 009000Z,00900ZZ,009030Z,00903ZZ,009040Z \| \| --- \| \| 00904ZZ,00C00ZZ,00C03ZZ,00C04ZZ,00H002Z \| \| 00H003Z,00H032Z,00H033Z,00H042Z,00H043Z \| \| 00H602Z,00H603Z,00H632Z,00H633Z,00H642Z \| \| 00H643Z,00P000Z,00P002Z,00P003Z,00P007Z \| \| 00P00JZ,00P00KZ,00P030Z,00P032Z,00P033Z \| \| 00P037Z,00P03JZ,00P03KZ,00P040Z,00P042Z \| \| 00P043Z,00P047Z,00P04JZ,00P04KZ,00P600Z \| \| 00P602Z,00P603Z,00P630Z,00P632Z,00P633Z \| \| 00P640Z,00P642Z,00P643Z,00P6X2Z,00W000Z \| \| 00W002Z,00W003Z,00W007Z,00W00MZ,00W030Z \| \| 00W032Z,00W033Z,00W037Z,00W03JZ,00W03KZ \| \| 00W03MZ,00W040Z,00W042Z,00W043Z,00W047Z \| \| 00W04JZ,00W04KZ,00W04MZ,00W600Z,00W602Z \| \| 00W603Z,00W60MZ,00W630Z,00W632Z,00W633Z \| \| 00W63MZ,00W640Z,00W642Z,00W643Z,00W64MZ \| |
|  |  | ex cereb meningeal les | 1.51 | \| 00510ZZ,00513ZZ,00514ZZ,00B10ZZ,00B13ZZ \| \| --- \| \| 00B14ZZ,00D10ZZ,00D13ZZ,00D14ZZ \| |
|  |  | brain lobectomy | 1.53 | 08QM3ZZ,08QL0ZZ,08QL3ZZ,08QM0ZZ |
|  |  | other brain excision | 1.59 | 08QL3ZZ,08QM3ZZ,08QL0ZZ,08QM0ZZ |
| Otolaryngologic | Pineal & pituitary | exc pituit les-transfron | 7.61 | 0G800ZZ,0,0G803ZZ,0G804ZZ,0GB00ZZ |
| Thoracic | Lobectomy or pneumonectomy | lobectomy of lung | 32.4 |  |
|  |  | complete pneumonectomy | 32.5 | 0BTL4ZZ,0BTK4ZZ,0BTM4ZZ |
|  |  | emphysema bleb plication | 32.21 | \| 0BQK4ZZ,0BQM7ZZ,0BQK8ZZ,0BQL7ZZ,0BQM3ZZ \| \| --- \| \| 0BQK7ZZ,0BQM8ZZ,0BQM0ZZ,0BQL0ZZ,0BQL3ZZ \| \| 0BQL8ZZ,0BQL4ZZ,0BQK0ZZ,0BQK3ZZ,0BQM4ZZ \| |
|  |  | lung vol reduction surg | 32.22 | \| 0BBK3ZZ,0B5K7ZZ,0BBM7ZZ,0B5K3ZZ,0BBL0ZZ \| \| --- \| \| 0B5M0ZZ,0B5L0ZZ,0BBL3ZZ,0B5M3ZZ,0BBK0ZZ \| \| 0B5M7ZZ,0BBM3ZZ,0BBL7ZZ,0B5L7ZZ,0B5K0ZZ \| \| 0B5L3ZZ,0BBM0ZZ,0BBK7ZZ \| |
|  |  | partial lobectomy of lung | 32.3 | \| 0BBG4ZZ,0BBD4ZZ,0BBC4ZZ,0BBL4ZZ,0BTH4ZZ \| \| --- \| \| 0BBF4ZZ,0BBK4ZZ,0BBJ4ZZ,0BBH4ZZ \| |
| Otolaryngologic | Major Larynx procedures | complete laryngectomy | 30.3 | \| 0B110F4,0,0B110Z4,0B113F4,0B113Z4 \| \| --- \| \| 0B114F4,0B114Z4,0CTS0ZZ,0CTS0ZZ,0CTS4ZZ \| \| 0CTS4ZZ,0CTS7ZZ,0CTS7ZZ,0CTS8ZZ,0CTS8ZZ \| |
|  | Major Tracheal/bronchial | bronchial repair nec | 33.48 | \| 0BV63ZZ,0BU84JZ,0BV54ZZ,0BV53CZ,0BV98DZ \| \| --- \| \| 0BV64DZ,0BV40ZZ,0BS70ZZ,0BV44CZ,0BSB0ZZ \| \| 0BVB7DZ,0BV90DZ,0BV58DZ,0BVB7ZZ,0BN74ZZ \| \| 0BMB0ZZ,0BV74DZ,0BF87ZZ,0BFB3ZZ,0BUB4JZ \| \| 0BV77ZZ,0BU747Z,0BQ93ZZ,0BFB7ZZ,0BV94ZZ \| \| 0BU44KZ,0BUB47Z,0BV50DZ,0BU807Z,0BV90CZ \| \| 0BU54JZ,0BQ57ZZ,0BVB3DZ,0BU407Z,0BV54DZ \| \| 0BQ97ZZ,0BV73CZ,0BS30ZZ,0BF60ZZ,0BU70JZ \| \| 0BV47DZ,0BF33ZZ,0BF83ZZ,0BV44ZZ,0BV93CZ \| \| 0BV83CZ,0BV90ZZ,0BU647Z,0BN70ZZ,0BV53ZZ \| \| 0BNB0ZZ,0BF48ZZ,0BQB7ZZ,0BU44JZ,0BU94JZ \| \| 0BV34CZ,0BVB3CZ,0BV50ZZ,0BQ37ZZ,0BNB4ZZ \| \| 0BF50ZZ,0BF63ZZ,0BU30KZ,0BV84ZZ,0BM30ZZ \| \| 0BQ48ZZ,0BS50ZZ,0BU60KZ,0BVB0ZZ,0BQ50ZZ \| \| 0BM70ZZ,0BV74CZ,0BU907Z,0BN73ZZ,0BVB4DZ \| \| 0BU947Z,0BUB0KZ,0BV33CZ,0BV37ZZ,0BF74ZZ \| \| 0BFB4ZZ,0BV50CZ,0BU34JZ,0BV43CZ,0BN60ZZ \| \| 0BQ94ZZ,0BV30DZ,0BU40KZ,0BV94DZ,0BN30ZZ \| \| 0BN44ZZ,0BV44DZ,0BF57ZZ,0BU34KZ,0BN58ZZ \| \| 0BNB8ZZ,0BF80ZZ,0BQ44ZZ,0BUB07Z,0BU90JZ \| \| 0BV67ZZ,0BU94KZ,0BU50KZ,0BF88ZZ,0BM50ZZ \| \| 0BV57DZ,0BV43DZ,0BF84ZZ,0BV84DZ,0BQ33ZZ \| \| 0BQ54ZZ,0BQ68ZZ,0BU30JZ,0BQ38ZZ,0BF40ZZ \| \| 0BN97ZZ,0BU84KZ,0BV48DZ,0BQB4ZZ,0BN87ZZ \| \| 0BF78ZZ,0BU80JZ,0BF38ZZ,0BV87ZZ,0BS60ZZ \| \| 0BN53ZZ,0BU447Z,0BQ98ZZ,0BQ53ZZ,0BN40ZZ \| \| 0BU507Z,0BQ58ZZ,0BV40CZ,0BU607Z,0BU90KZ \| \| 0BF34ZZ,0BU347Z,0BV37DZ,0BV68ZZ,0BV93ZZ \| \| 0BN84ZZ,0BVB0DZ,0BQ60ZZ,0BQB0ZZ,0BU707Z \| \| 0BN93ZZ,0BN68ZZ,0BV80ZZ,0BV53DZ,0BS80ZZ \| \| 0BVB4ZZ,0BN34ZZ,0BU70KZ,0BV88ZZ,0BV78DZ \| \| 0BV67DZ,0BF90ZZ,0BU547Z,0BF68ZZ,0BS90ZZ \| \| 0BQ80ZZ,0BU50JZ,0BQ47ZZ,0BN90ZZ,0BU847Z \| \| 0BV63DZ,0BU40JZ,0BV48ZZ,0BNB3ZZ,0BQ67ZZ \| \| 0BS40ZZ,0BV38ZZ,0BF53ZZ,0BU54KZ,0BV47ZZ \| \| 0BF64ZZ,0BV84CZ,0BFB8ZZ,0BV60ZZ,0BV80CZ \| \| 0BN50ZZ,0BQ83ZZ,0BN47ZZ,0BQ40ZZ,0BV93DZ \| \| 0BV87DZ,0BQ63ZZ,0BQB3ZZ,0BU64KZ,0BQ73ZZ \| \| 0BV78ZZ,0BV74ZZ,0BN78ZZ,0BU74JZ,0BQ43ZZ \| \| 0BF98ZZ,0BF73ZZ,0BN63ZZ,0BVB3ZZ,0BV33ZZ \| \| 0BF97ZZ,0BQ77ZZ,0BV97ZZ,0BN38ZZ,0BN57ZZ \| \| 0BV64ZZ,0BUB4KZ,0BQ88ZZ,0BN67ZZ,0BV58ZZ \| \| 0BF37ZZ,0BQ64ZZ,0BM40ZZ,0BV88DZ,0BQ30ZZ \| \| 0BVB4CZ,0BN48ZZ,0BN83ZZ,0BF70ZZ,0BF43ZZ \| \| 0BV60CZ,0BN54ZZ,0BV63CZ,0BV57ZZ,0BV80DZ \| \| 0BQ87ZZ,0BV83ZZ,0BQ34ZZ,0BV60DZ,0BU64JZ \| \| 0BV73ZZ,0BVB8ZZ,0BN98ZZ,0BF44ZZ,0BV70ZZ \| \| 0BVB0CZ,0BU307Z,0BQ70ZZ,0BM90ZZ,0BF67ZZ \| \| 0BF54ZZ,0BN80ZZ,0BN88ZZ,0BV64CZ,0BV30CZ \| \| 0BF93ZZ,0BUB0JZ,0BV34DZ,0BV70DZ,0BV98ZZ \| \| 0BF47ZZ,0BQB8ZZ,0BN94ZZ,0BU74KZ,0BV34ZZ \| \| 0BN43ZZ,0BV30ZZ,0BM80ZZ,0BQ90ZZ,0BF30ZZ \| \| 0BFB0ZZ,0BV40DZ,0BN37ZZ,0BQ84ZZ,0BV73DZ \| \| 0BV70CZ,0BV54CZ,0BF77ZZ,0BF58ZZ,0BN77ZZ \| \| 0BV83DZ,0BV38DZ,0BN64ZZ,0BQ74ZZ,0BU80KZ \| \| 0BM60ZZ,0BN33ZZ,0BV77DZ,0BF94ZZ,0BQ78ZZ \| \| 0BV94CZ,0BVB8DZ,0BV97DZ,0BU60JZ,0BNB7ZZ \| \| 0BV68DZ,0BV33DZ,0BV43ZZ \| |
| Thoracic | Extrapulmonary procedure | decortication of lung | 34.51 | \| 0BDN0ZX,0BDN3ZZ,0BDN0ZZ,0BDP3ZX,0BDP0ZZ \| \| --- \| \| 0BDN3ZX,0BDP3ZZ,0BDP0ZX \| |
|  |  | other pleural excision | 34.59 | \| 0BBP4ZZ,0BBN4ZZ,0BBP3ZZ,0BBP0ZZ,0BBN0ZZ \| \| --- \| \| 0BBN3ZZ \| |
|  |  | clos thoracic fistul nec | 34.73 | \| 0WQ84ZZ,0WQ83ZZ,0WQC4ZZ,0BQ33ZZ,0BQ80ZZ \| \| --- \| \| 0BQ50ZZ,0BQ97ZZ,0BQ38ZZ,0BQN3ZZ,0BQ84ZZ \| \| 0BQP0ZZ,0BQ44ZZ,0BQB3ZZ,0BQN4ZZ,0BQ83ZZ \| \| 0BQ84ZZ,0BQ83ZZ,0BQ30ZZ,0WQ80ZZ,0WQ80ZZ \| \| 0BQK7ZZ,0BQ93ZZ,0BQ48ZZ,0BQ53ZZ,0BQ33ZZ \| \| 0FQ03ZZ,0WQ84ZZ,0BQ64ZZ,0BQP3ZZ,0BQP4ZZ \| \| 0BQ88ZZ,0BQB3ZZ,0BQ33ZZ,0BQP3ZZ,0BQ53ZZ \| \| 0WQ83ZZ,0BQ50ZZ,0BQ77ZZ,0BQ68ZZ,0BQP3ZZ \| \| 0BQP4ZZ,0BQ77ZZ,0BQ67ZZ,0WQC3ZZ,0WQ84ZZ \| \| 0BQB0ZZ,0BQ73ZZ,0BQ38ZZ,0BQ43ZZ,0BQM3ZZ \| \| 0BQ78ZZ,0BQB0ZZ,0BQ97ZZ,0BQ57ZZ,0BQN3ZZ \| \| 0BQ30ZZ,0BQ88ZZ,0BQM4ZZ,0BQ63ZZ,0BQ53ZZ \| \| 0BQB0ZZ,0BQP0ZZ,0BQ38ZZ,0WQC0ZZ,0BQ68ZZ \| \| 0BQ70ZZ,0WQC0ZZ,0BQ48ZZ,0BQ44ZZ,0BQM8ZZ \| \| 0BQB4ZZ,0BQM0ZZ,0BQN4ZZ,0BQ54ZZ,0BQ94ZZ \| \| 0WQ83ZZ,0FQ00ZZ,0BQ43ZZ,0BQ64ZZ,0BQ98ZZ \| \| 0BQN4ZZ,0BQ30ZZ,0BQP0ZZ,0BQ37ZZ,0FQ00ZZ \| \| 0BQ63ZZ,0BQN3ZZ,0BQN0ZZ,0BQ77ZZ,0BQB8ZZ \| \| 0BQ90ZZ,0BQL7ZZ,0BQ68ZZ,0BQ78ZZ,0BQM7ZZ \| \| 0DQ53ZZ,0BQN3ZZ,0WQ80ZZ,0BQB4ZZ,0BQN0ZZ \| \| 0BQ84ZZ,0BQ34ZZ,0WQC4ZZ,0BQK0ZZ,0BQ37ZZ \| \| 0BQN3ZZ,0BQ63ZZ,0BQ40ZZ,0BQ33ZZ,0BQP3ZZ \| \| 0BQ84ZZ,0BQ98ZZ,0BQ94ZZ,0BQ74ZZ,0BQN4ZZ \| \| 0BQ74ZZ,0BQL4ZZ,0BQB0ZZ,0BQ97ZZ,0BQ47ZZ \| \| 0BQP4ZZ,0BQ43ZZ,0BQ94ZZ,0BQ90ZZ,0BQ74ZZ \| \| 0WQC3ZZ,0WQC4ZZ,0BQN0ZZ,0BQ47ZZ,0BQ83ZZ \| \| 0BQ93ZZ,0BQK8ZZ,0BQ88ZZ,0BQB4ZZ,0BQ40ZZ \| \| 0BQ48ZZ,0BQB7ZZ,0BQ60ZZ,0BQ44ZZ,0BQ94ZZ \| \| 0BQP0ZZ,0BQ43ZZ,0BQ60ZZ,0BQ80ZZ,0BQN3ZZ \| \| 0BQ60ZZ,0BQ67ZZ,0BQ87ZZ,0BQ68ZZ,0BQ57ZZ \| \| 0BQ73ZZ,0BQ48ZZ,0BQ50ZZ,0BQ57ZZ,0BQB7ZZ \| \| 0BQ80ZZ,0BQB4ZZ,0BQ98ZZ,0WQ84ZZ,0BQ70ZZ \| \| 0BQ57ZZ,0BQ93ZZ,0BQ90ZZ,0BQP0ZZ,0BQ87ZZ \| \| 0BQ97ZZ,0BQ34ZZ,0BQP4ZZ,0BQ73ZZ,0DQ58ZZ \| \| 0BQ58ZZ,0BQB3ZZ,0BQB7ZZ,0BQ64ZZ,0BQ58ZZ \| \| 0BQ78ZZ,0BQP4ZZ,0BQN4ZZ,0BQN4ZZ,0BQ90ZZ \| \| 0BQ63ZZ,0BQP4ZZ,0BQ54ZZ,0DQ54ZZ,0BQ88ZZ \| \| 0WQC3ZZ,0FQ04ZZ,0BQ40ZZ,0BQB3ZZ,0BQ70ZZ \| \| 0BQ40ZZ,0BQ98ZZ,0BQL3ZZ,0DQ57ZZ,0BQ38ZZ \| \| 0BQ80ZZ,0BQ44ZZ,0BQP3ZZ,0BQ60ZZ,0BQ87ZZ \| \| 0BQ58ZZ,0BQ34ZZ,0BQ78ZZ,0BQL8ZZ,0BQK4ZZ \| \| 0BQ73ZZ,0BQB8ZZ,0BQB8ZZ,0BQ54ZZ,0BQ50ZZ \| \| 0WQ83ZZ,0BQN0ZZ,0BQ74ZZ,0BQ54ZZ,0FQ03ZZ \| \| 0BQ67ZZ,0BQ64ZZ,0BQ37ZZ,0BQP3ZZ,0BQ70ZZ \| \| 0BQB7ZZ,0BQL0ZZ,0BQB8ZZ,0WQC0ZZ,0DQ50ZZ \| \| 0BQ37ZZ,0BQP0ZZ,0BQ47ZZ,0BQ34ZZ,0BQ53ZZ \| \| 0BQ30ZZ,0BQ87ZZ,0BQ93ZZ,0BQ67ZZ,0BQN0ZZ \| \| 0WQ80ZZ,0BQ47ZZ,0BQ58ZZ,0BQ83ZZ,0BQN0ZZ \| \| 0FQ04ZZ,0BQK3ZZ,0BQ77ZZ \| |
| Cardiac | Mediastinal procedure | incision of mediastinum | 34.1 | \| 0W9C00Z,0W9C0ZZ,0W9C30Z,0W9C3ZZ,0W9C40Z \| \| --- \| \| 0W9C4ZZ,0WCC0ZZ,0WCC3ZZ,0WCC4ZZ \| |
|  | Heart valve procedures | opn aortic valvuloplasty | 35.11 | 02QF0ZZ,027F0ZZ,02NF0ZZ,027F0DZ,027F04Z |
|  |  | opn mitral valvuloplasty | 35.12 | 02QG0ZZ,027G04Z,027G0DZ,027G0ZZ,02NG0ZZ |
|  |  | opn tricus valvuloplasty | 35.14 | 027J0DZ,02NJ0ZZ,027J04Z,027J0ZZ,02QJ0ZZ |
|  |  | replace aort valv-tissue | 35.21 | \| 02RF08Z,02RF07Z,02RF0KZ,02RF4KZ,02RF47Z \| \| --- \| \| 02RF48Z \| |
|  |  | replace aortic valve nec | 35.22 | 02RF0JZ,02RF4JZ |
|  |  | replace mitr valv-tissue | 35.23 | \| 02RG3KZ,02RG07Z,02RG08Z,02RG48Z,02RG47Z \| \| --- \| \| 02RG0KZ,02RG37Z,02RG38Z,02RG4KZ \| |
|  |  | replace mitral valve nec | 35.24 | 02RG4JZ,02RG0JZ,02RG3JZ |
|  |  | replace tric valv-tissue | 35.27 | \| 02RJ48Z,02RJ4KZ,02RJ08Z,02RJ07Z,02RJ0KZ \| \| --- \| \| 02RJ47Z \| |
|  |  | replace tricusp valv nec | 35.28 | 02RJ0JZ,02RJ4JZ |
|  | Coronary artery bypass graft (CABG) | aortocor bypas-1 cor art | 36.11 | \| 021009W,02100KW,02104AW,02104KW,021049W \| \| --- \| \| 02104JW,02100AW,02100JW \| |
|  |  | aortocor bypas-2 cor art | 36.12 | \| 021149W,02110KW,021109W,02114JW,02110JW \| \| --- \| \| 02110AW,02114KW,02114AW \| |
|  |  | aortocor bypas-3 cor art | 36.13 | \| 021249W,02124KW,02124JW,02120JW,02120AW \| \| --- \| \| 02120KW,02124AW,021209W \| |
|  |  | aortcor bypas-4+ cor art | 36.14 | \| 02130AW,02130JW,02130KW,021349W,02134JW \| \| --- \| \| 02134KW,021309W,02134AW \| |
|  |  | 1 int mam-cor art bypass | 36.15 | \| 02100JC,02100J8,02100A8,02104JC,02104A9 \| \| --- \| \| 210099,02100KC,02104K9,210498,02104K8 \| \| 02104Z9,021049C,02104A8,02100K9,02100AC \| \| 02100K8,02104KC,02100Z9,02104Z8,02104J8 \| \| 02100ZC,210499,02100J9,02104J9,02100Z8 \| \| 02100A9,210098,02104AC,021009C,02104ZC \| |
|  |  | 2 int mam-cor art bypass | 36.16 | \| 02114AC,02114KC,02110J8,02124AC,02110AC \| \| --- \| \| 02130ZC,02134ZC,211098,02134JC,211498 \| \| 02114J9,021349C,211499,02124JC,02114K9 \| \| 211099,02114Z9,02110Z8,021109C,02114JC \| \| 02130JC,02110ZC,02134KC,02114ZC,02120KC \| \| 021309C,02124ZC,02120JC,02110KC,02130AC \| \| 02114A9,02130KC,02120ZC,02110A9,02110A8 \| \| 02134AC,021249C,02114K8,02110J9,02114Z8 \| \| 02124KC,02110Z9,02110JC,02114J8,021209C \| \| 02114A8,02120AC,021149C,02110K9,02110K8 \| |
|  |  | open chest trans revasc | 36.31 | 021L0Z5,021K0Z5 |
|  | Pericardial/Extracardiac | heart & pericard repair# | 37.4 | \| 0W9D0ZZ,02NN3ZZ,0WCD4ZZ,02NN0ZZ,02CN4ZZ \| \| --- \| \| 02CN0ZZ,02NN4ZZ,0W9D0ZX,0W9D00Z,0WCD0ZZ \| \| 0WCD3ZZ,02CN3ZZ \| |
|  |  | pericardiotomy | 37.12 | \| 0W9D0ZZ,02NN3ZZ,0WCD4ZZ,02NN0ZZ,02CN4ZZ \| \| --- \| \| 02CN0ZZ,02NN4ZZ,0W9D0ZX,0W9D00Z,0WCD0ZZ \| \| 0WCD3ZZ,02CN3ZZ \| |
|  |  | pericardiectomy | 37.31 | \| 02BN3ZZ,02TN4ZZ,02BN0ZZ,02TN0ZZ,02BN4ZZ \| \| --- \| \| 02TN3ZZ \| |
|  | Intracardiac | annuloplasty | 35.33 | \| 02QJ0ZZ,02QG3ZZ,02QH0ZZ,02QH4ZZ,02QF4ZZ \| \| --- \| \| 02QJ4ZZ,02QH3ZZ,02QJ3ZZ,02QG4ZZ,02QF0ZZ \| \| 02QG0ZZ,02QF3ZZ \| |
|  |  | pros rep ventric def-opn | 35.53 | 02UM4JZ,02UM3JZ,02RM0JZ,02UM0JZ |
|  |  | graft repair ventric def | 35.62 | \| 02UM07Z,02RM07Z,02UM38Z,02RM0KZ,02RM47Z \| \| --- \| \| 02UM48Z,02UM0KZ,02RM4KZ \| |
|  |  | ventr septa def rep nec | 35.72 | 02QM4ZZ,02QM0ZZ,02QM4ZZ,02QM3ZZ |
|  |  | conduit left ventr-aorta | 35.93 | 021L0ZW,021L4ZW |
|  |  | cardiotomy | 37.11 | \| 02CK3ZZ,02CL3ZZ,02C60ZZ,02C84ZZ,02CL4ZZ \| \| --- \| \| 02C93ZZ,02C90ZZ,02CK0ZZ,02C83ZZ,02CL0ZZ \| \| 02C73ZZ,02C80ZZ,02C70ZZ,02CK4ZZ,02C74ZZ \| \| 02C63ZZ,02C64ZZ,02C94ZZ \| |
|  |  | heart aneurysm excision | 37.32 | \| 02B64ZZ,02B73ZZ,02BL3ZZ,02B60ZZ,02BK3ZZ \| \| --- \| \| 02BL4ZZ,02BK4ZZ,02BL0ZZ,02B63ZZ,02BK0ZZ \| \| 02B70ZZ,02B74ZZ \| |
|  |  | exc/dest hrt lesion open | 37.33 | \| 02B70ZZ,025L0ZZ,02560ZZ,02570ZZ,025K0ZZ \| \| --- \| \| 02BK0ZZ,02T80ZZ,02B60ZZ,02BL0ZZ \| |
|  |  | partial ventriculectomy | 37.35 | \| 02BL3ZZ,02BK3ZZ,02BK0ZZ,02BL4ZZ,02BK4ZZ \| \| --- \| \| 02BL0ZZ \| |
|  | Other Coronary Artery procedure | open coronry angioplasty | 36.03 | \| 02C10ZZ,02730ZZ,02720ZZ,02C20ZZ,02C30ZZ \| \| --- \| \| 02710ZZ,02700ZZ,02C00ZZ \| |
|  |  | rem of cor art obstr nec | 36.09 | \| 02C13ZZ,02C33ZZ,02C03ZZ,02C04ZZ,02C14ZZ \| \| --- \| \| 02C23ZZ,02C34ZZ,02C24ZZ \| |
|  |  | heart vessel op nec | 36.99 | \| 02Q00ZZ,02Q04ZZ,02Q43ZZ,02Q44ZZ,02Q40ZZ \| \| --- \| \| 02Q03ZZ \| |
|  | Artificial Heart/Assist Devices | insrt non-impl circ dev | 37.62 | 5A02216,5A02116 |
|  |  | repair heart assist sys | 37.63 | \| 02WA3QZ,02WA0RZ,02WA3RZ,02WA4QZ,02WA4RZ \| \| --- \| \| 02WA0QZ \| |
|  |  | imp vent ext hrt ast sys | 37.65 | 02HA0RZ |
|  |  | implantable hrt assist | 37.66 | \| 02HA3QZ,02HA0QZ,02HA4QZ,02HA3QZ,02HA0QZ \| \| --- \| \| 02HA4QZ,5A02216,5A02116 \| |
|  | Aortic resection; replacement of anastomosis | aorta resection & anast | 38.34 | 02BW4ZZ,02BW0ZZ,04B00ZZ,04B04ZZ |
|  |  | resect abdm aorta w repl | 38.44 | \| 04R00JZ,04R04JZ,04R047Z,04R007Z,04R04KZ \| \| --- \| \| 04R00KZ \| |
|  |  | excision of aorta | 38.64 | \| 04B03ZZ,04503ZZ,04504ZZ,04B04ZZ,04B00ZZ \| \| --- \| \| 04500ZZ \| |
|  |  | endo imp grft thor aorta | 39.73 | 02VW4DZ,02VW0DZ,02UW3JZ,02UW4JZ,02VW3DZ |
|  | Peripheral Vascular Bypass | aorta-iliac-femor bypass | 39.25 | \| 041C0JK,041J4JH,04104JR,041D4ZH,041F0ZK \| \| --- \| \| 041C0KH,041H09J,041F0JJ,041049Q,04100JB \| \| 041F0ZJ,041C4JH,04100ZH,04104A9,041E0KK \| \| 041E0AJ,041D09K,041E0ZJ,04100A6,041F4AH \| \| 041J4KK,041C4JK,041H49H,041E0JJ,041E49J \| \| 041D0KK,041F4JK,041C09H,041E4ZK,041D0JH \| \| 041H0AH,410096,041J4JJ,041009H,04104AB \| \| 04100ZF,041E09H,04100AQ,04100ZB,041C4ZH \| \| 04100KC,04100AK,041H49J,04104ZG,041D0AJ \| \| 041J4JK,041J0JJ,04104K8,04104ZR,04100K9 \| \| 041H0AJ,041F4ZH,041J09K,041H4AK,041H4ZK \| \| 410496,041F0AH,041F09K,041H4ZH,04100JF \| \| 041E4AK,041C0ZK,04104JQ,041F0JK,041D09H \| \| 410499,04100KD,041H4AJ,041C0AJ,041H0ZK \| \| 041D0ZJ,04104A6,041J4ZH,04104KC,04104ZC \| \| 04100JC,041E0KJ,041D49J,041H09K,041H0ZJ \| \| 041E0ZK,041H0KK,041E4AJ,041H0JJ,041C09J \| \| 041H4KH,041009G,04100JD,04104Z8,041E4JK \| \| 041D0AK,041D4ZJ,041D09J,041C09K,041009J \| \| 04100AB,041J0JK,04104A8,04100JQ,041C49H \| \| 04104J8,041C4ZK,04104J9,04100ZJ,041049F \| \| 041F0KK,041H4AH,04104A7,04104K6,041C4AJ \| \| 04100JH,04100A9,04100JJ,041J0KJ,041009D \| \| 041J49K,041D49K,04104Z9,041J0AJ,04104KJ \| \| 041C0AH,041C0ZH,041D0AH,041F0AJ,041F4ZJ \| \| 04100KF,041E49K,04100AJ,04104KF,041E0ZH \| \| 041H4KK,041F09J,04104JB,041J4ZK,041D0ZH \| \| 041J0JH,041049C,04104ZJ,04100J7,04100J9 \| \| 041D0JJ,041J0ZH,041C0JJ,041049D,04100KH \| \| 04104AK,041H4ZJ,041J0ZJ,04104KR,041049B \| \| 04104ZB,041C49K,041F49J,04100ZG,041D4JK \| \| 04100KQ,04100KK,04104ZH,04104K9,04104JD \| \| 041J4AJ,04104JK,04100K7,04104AH,04100AC \| \| 041J0KH,04100KR,041C0ZJ,04104KD,041E4AH \| \| 04104AR,041049R,041D0ZK,041009B,041H09H \| \| 04104KQ,041F4ZK,041J0ZK,04100Z7,04104Z7 \| \| 04100KJ,04104J6,041C4ZJ,041E4KK,041E4ZH \| \| 041E4KH,410097,04100JR,041J4KH,041D4KJ \| \| 041D0KH,04104KK,04100AR,04100ZC,04104JC \| \| 041E4KJ,041C0KK,041H0AK,04100J6,041F0JH \| \| 041H4JH,041E4JJ,041D0KJ,041C4AK,04104JG \| \| 04100ZD,041D4KH,041E4JH,041F0KJ,041J4ZJ \| \| 041E0JH,04100KG,04100K6,041009K,04104KH \| \| 04104J7,04100ZQ,041E09K,041C0JH,041F09H \| \| 04104AD,041J4KJ,041C0AK,041J49J,041J09H \| \| 041H4JJ,041D0JK,041J0KK,041H0KH,041J4AH \| \| 04100Z6,04100AD,04100Z8,041C0KJ,041J09J \| \| 041D4KK,041C4AH,04104JH,04100Z9,041009Q \| \| 041E0AK,041C4KJ,041H49K,041H4KJ,041F49K \| \| 04100KB,04104JJ,041D4JJ,04104K7,04100J8 \| \| 041J49H,041049J,041E49H,041009R,041D4AH \| \| 04100AH,041H0KJ,041C4KK,04104ZQ,04104ZD \| \| 041D4AK,410498,04100A7,410098,041F0ZH \| \| 041H4JK,04100ZK,041D4ZK,041H0JH,041E4ZJ \| \| 041049K,041D49H,041E0AH,041F4JH,04100ZR \| \| 041F4AJ,04104ZK,041009F,04104AF,041J0AH \| \| 041D4AJ,04104KB,041F4AK,04104AC,041H0JK \| \| 04104AQ,04104AJ,410099,041009C,041D4JH \| \| 041J0AK,041F4JJ,04100K8,041049H,041F0KH \| \| 041F4KK,04104JF,04104KG,041C49J,041E09J \| \| 04100A8,041C4KH,041C4JJ,04100JK,04100JG \| \| 04104Z6,041049G,041J4AK,041F0AK,041F49H \| \| 04100AF,041E0KH,041F4KH,041H0ZH,410497 \| \| 041E0JK,041F4KJ,04104AG,04100AG,04104ZF \| |
|  |  | vasc shunt & bypass nec | 39.29 | \| 051G0KY,041K0KL,313097,316090,03150JF \| \| --- \| \| 061V4JY,041K09S,031A0ZF,051A07Y,316098 \| \| 03190KF,051N4JY,031209B,03130Z6,03150A8 \| \| 03140J3,051T0ZY,031C0AF,041L0KS,051M47Y \| \| 03140A9,041K09L,041K0ZH,051G07Y,03130A0 \| \| 03120KC,03160J5,03150ZJ,03140K9,051V0ZY \| \| 051L0AY,061N4KY,051H47Y,031B093,03130K6 \| \| 031609C,061M0AY,061M0JY,031B09F,041L0AJ \| \| 041K0AN,061F0JY,03160AJ,03140ZK,041K4JL \| \| 061C07Y,061G09Y,03140KC,061309Y,061R4JY \| \| 041K0AL,031J0AG,051G4KY,03150ZF,061Q0JY \| \| 041K4JK,03120KJ,03120AD,031609D,03130KB \| \| 03130K2,031G0AG,051F4JY,03150J3,03150KC \| \| 03120ZJ,031A09F,03120Z0,03130Z1,03140J9 \| \| 051G0AY,051S07Y,03150Z3,03160A6,041L4KM \| \| 03130K0,03160J6,03130A7,041L4AQ,05174KY \| \| 041K4ZL,041N4JQ,03160Z0,03170JF,041L0JM \| \| 06130AY,03170JD,041N4KS,03140JC,031A0J4 \| \| 03160ZC,051V0AY,061V4AY,041K0AM,051V0JY \| \| 05180JY,031J0KG,061P07Y,314090,041L0JK \| \| 041L09Q,041K4ZN,031H0JG,041M4AM,061R0AY \| \| 03160K2,061F4AY,051R0KY,041M0JS,051T49Y \| \| 051P4KY,03190Z3,03160AC,03140JF,05174JY \| \| 03150KD,061G0KY,051R4JY,06130ZY,06134KY \| \| 313091,03120ZK,051Q07Y,03120Z1,041K0JN \| \| 051H0JY,051B0ZY,051T0AY,03160K9,061G4JY \| \| 041K4KH,061M4ZY,03180JD,061S49Y,051C07Y \| \| 031A0K4,051V4ZY,041N0JL,05184JY,041K09Q \| \| 031809F,061R47Y,03160Z4,316095,041K4AJ \| \| 051S49Y,03160Z2,03160K4,051F0ZY,03140Z7 \| \| 061S0KY,061Q09Y,051L0KY,041L09H,041L0JJ \| \| 03170Z0,03130K1,05194ZY,041K49M,041M49L \| \| 041M0KP,041M4AQ,03160J8,051C4JY,041N4ZS \| \| 03150A7,041K4ZH,061H07Y,03130JC,041K0AK \| \| 03160J2,061V4ZY,041L4JS,03160JD,041L4JH \| \| 03150J7,061F0KY,041K0AS,03140A1,061M4AY \| \| 03120K3,061T4ZY,03160ZF,06134ZY,061S09Y \| \| 041L49K,061N0KY,061S4AY,03130J9,051H09Y \| \| 041N4ZM,061R0ZY,03180K4,312093,041K4JJ \| \| 03130A4,051A4AY,03140KJ,051Q0ZY,318091 \| \| 03130AJ,061C47Y,061H0AY,041L09S,041M0JM \| \| 061M4KY,041K4JN,041M0ZP,051A09Y,041N0AS \| \| 03120JF,051G47Y,031809D,031J0ZG,051P09Y \| \| 03140J7,061T0KY,03160A5,03130J6,03160K0 \| \| 061P0KY,03130Z2,061347Y,03150K3,03150A4 \| \| 03120JB,041K09J,051A49Y,031G0JG,03120Z9 \| \| 03170A3,03120K1,061P49Y,041K0KM,061G47Y \| \| 03140A8,051D09Y,031G09G,041L0JP,031A0JF \| \| 03130AF,061G07Y,03160A0,031C09F,061N47Y \| \| 041L0AM,03120A7,041L49H,03130AK,031B0A3 \| \| 315096,05190KY,061C09Y,041K0ZJ,031C0ZF \| \| 051G09Y,03160KD,041K0KQ,061C0ZY,041N4AQ \| \| 03170ZF,03150AJ,041K49P,03160JF,051S4JY \| \| 041K4KQ,03140JD,051F0KY,03120A8,03120A3 \| \| 03160Z9,051C49Y,061H0JY,041M4JS,03120KB \| \| 041N0ZL,051G0JY,041L09J,031C0A4,03150AD \| \| 315092,041M49P,03160J4,061N0ZY,041M0ZL \| \| 041K0ZQ,051847Y,03150K7,031C0J4,061Q4KY \| \| 041K4KN,061Q0ZY,041N49P,041K4AH,05184AY \| \| 041N09P,041M09S,05170KY,03140JK,03120KD \| \| 03160JC,051H07Y,041K4KS,03160AB,031509C \| \| 051V4AY,03130A1,041N0KL,051C47Y,061Q4AY \| \| 03120K0,06134JY,312095,031A0A4,315090 \| \| 031609B,03140J1,041M0ZQ,041K0AJ,051P4ZY \| \| 03180ZD,03150J1,05194AY,031C0KF,051A47Y \| \| 061V09Y,03130K7,051S4AY,03150K9,051P0JY \| \| 03140AF,051M49Y,051M0ZY,061N09Y,03120K4 \| \| 041M4ZP,041L4KP,05190AY,051T0JY,061N4AY \| \| 051G4ZY,051G49Y,051907Y,061H4JY,031209J \| \| 03120J3,051R07Y,041M0KQ,041K4KM,03160Z6 \| \| 041N49S,041L4KS,041M4KQ,03130ZK,031H0ZG \| \| 041M4KS,03150JJ,315095,03140K8,061N49Y \| \| 03120K9,316091,041M09L,041L0JQ,041N09M \| \| 051N4AY,051R0JY,312091,041M09Q,314094 \| \| 03120A0,061C49Y,041N4KP,041M4JM,041K49S \| \| 03130Z5,03130ZJ,061D0ZY,041M4KL,314099 \| \| 031B0ZF,051B09Y,03140K5,051D0KY,051S0JY \| \| 061P09Y,041K0KS,031209K,061M49Y,03120Z6 \| \| 041N0ZQ,031C0JF,041K0KK,051C09Y,03130Z8 \| \| 03150K2,031A0KF,041M0JQ,061P0ZY,031209D \| \| 041K4AP,03180J4,03120J1,03170ZD,051M09Y \| \| 03150J8,03130AB,041N4ZL,03130JB,03120Z4 \| \| 051707Y,03130AD,061V0JY,041N49M,03140ZF \| \| 041M4ZQ,03150Z4,061S47Y,061D09Y,041K4ZS \| \| 041K4JM,03120A4,312092,041L0ZL,03160A9 \| \| 03160A2,041K4KJ,03140A3,314095,051P49Y \| \| 041L4ZP,051M0KY,061G0JY,03150A6,041L09P \| \| 061Q49Y,03150A5,031609K,041K4AL,316096 \| \| 051C0JY,061V0KY,03140JJ,031509B,051M0JY \| \| 03150Z7,061C0JY,05170AY,061M0ZY,061N4ZY \| \| 03130ZB,031C0K4,061V4KY,041K0ZK,061R4AY \| \| 03130Z3,051N09Y,061F07Y,041K0ZN,03140KK \| \| 03120JD,041M4AL,03170Z3,03130A9,317090 \| \| 03160J7,051P07Y,051B0KY,03150JB,051N0KY \| \| 051D4JY,031509D,051S0KY,03140J4,061V07Y \| \| 051M4ZY,03130J3,041M0AQ,041L0KP,03120ZF \| \| 041K0KP,061T0ZY,03150ZD,03130KJ,041K0JK \| \| 051B07Y,061D47Y,041L4JP,03150K5,051M4AY \| \| 051S4ZY,051B4AY,051V07Y,041M0KM,041L4KJ \| \| 03190A3,03160K5,051F49Y,041M4JQ,316092 \| \| 051A0JY,061R0JY,315097,03180K1,051V0KY \| \| 03140A6,041K0KJ,03160A3,061T47Y,03140Z6 \| \| 041L49L,312098,061S4ZY,03160Z3,051949Y \| \| 061T4KY,03180A1,061N0AY,03150K8,315099 \| \| 031B0K3,051B4ZY,061M09Y,051D0JY,03150AK \| \| 03120ZD,03150JK,051Q0KY,061H0ZY,041L0ZS \| \| 03140AC,03150AB,041N0KS,03120AJ,041L4ZQ \| \| 03130Z0,03150Z5,03130KD,041K4ZM,03120J9 \| \| 051T09Y,051L4KY,041L4JL,041M49M,03140Z8 \| \| 051H4ZY,03150J2,316094,03150ZC,061C4ZY \| \| 061F49Y,051P0KY,03140Z4,041N09Q,061H47Y \| \| 051A0KY,319093,031G0KG,03190AF,317093 \| \| 051Q47Y,03160AF,051B47Y,03150A3,051C4AY \| \| 041K0AQ,061C0AY,03120ZC,051L0JY,03160ZJ \| \| 041L4AL,051H4JY,03120J4,03160KF,03140Z1 \| \| 061T09Y,041K0JP,041L09M,03140ZJ,313096 \| \| 051L4ZY,041L09K,061G4KY,041L4JM,041N0JP \| \| 041L4AP,041L0ZJ,051N47Y,03130K3,051M07Y \| \| 031C094,041L0ZQ,041N09L,041K4AQ,041L0JH \| \| 061T0JY,051M0AY,051S47Y,03120Z2,03130KK \| \| 031B0AF,05170JY,312099,031309C,03150Z6 \| \| 041L0AS,051D49Y,041M0KL,051A4KY,03150ZK \| \| 061H0KY,061S4JY,03120K8,041L0KJ,051849Y \| \| 031G0ZG,051N0ZY,051F07Y,061T0AY,03160J0 \| \| 03140KD,061C0KY,03160K6,061H4KY,041L49N \| \| 061D4ZY,041M4KM,041L0KH,03180AD,041L0KL \| \| 051A0AY,031B0Z3,061Q4JY,03170A0,061M4JY \| \| 03140Z2,03160AD,03160J1,041N0ZP,051L07Y \| \| 03120AB,312096,03140K0,03150ZB,03150Z1 \| \| 061S4KY,041L49P,03140KB,061Q0KY,061M0KY \| \| 041L0JL,041N0AL,051C4ZY,03130A6,03130K5 \| \| 051L4AY,03140ZB,041K0AP,031509F,061R0KY \| \| 03140Z9,061P4ZY,315091,03120J5,041L4JN \| \| 03150J5,03140J8,051V49Y,03140J2,051R0ZY \| \| 041M0AL,03120K7,051H0KY,03190JF,051B4KY \| \| 041L49J,03130ZD,041K4ZQ,03180J1,041L4ZH \| \| 041K09P,03150J6,03160A8,041L4ZS,315093 \| \| 041L4AM,051A4JY,061C4KY,314097,03160JB \| \| 041K49H,031409F,03160A7,051809Y,031609J \| \| 051Q49Y,03130Z4,051Q4JY,05190JY,061S0JY \| \| 041M49S,03120ZB,061H49Y,031H0AG,041L0AQ \| \| 051Q4KY,03140AK,061F0AY,03130A2,051A0ZY \| \| 041M4JL,051D4ZY,03130ZC,031309K,031209F \| \| 03140A0,03120J0,03160ZD,031B0KF,061G0ZY \| \| 031409B,03120A6,041K0JJ,03160A1,041K4JP \| \| 061D4JY,041K0JH,031B0JF,03140K1,03120A1 \| \| 03150K0,041K49K,041N4JL,061G4AY,031909F \| \| 03170J0,051T4JY,041L0KK,041K4KK,041N4AP \| \| 03120J8,051R47Y,051D4KY,041K49L,041L0ZH \| \| 041M0ZM,03120A5,313090,318094,041L4AS \| \| 03160J9,051M4KY,051C0ZY,03170AD,313099 \| \| 03140A2,03140AJ,03120K6,051V09Y,03130A5 \| \| 041K4AK,051R4AY,051Q0AY,05180ZY,041M4JP \| \| 05194JY,041N49Q,03140K6,03160Z1,061R49Y \| \| 031H0KG,031309D,041M4AS,041N4JP,03160KC \| \| 041K09M,03130KC,061N0JY,041K09K,041N0ZM \| \| 03140Z3,031509J,051807Y,061D4KY,03150AF \| \| 041N0JQ,051T4ZY,051V47Y,041L0ZM,03130K8 \| \| 061D07Y,05184KY,041N4KQ,03180A4,051N07Y \| \| 051A4ZY,03150J0,03140ZC,061P0AY,051F0AY \| \| 061F09Y,041N4JS,03180KD,041M0AM,041L4KQ \| \| 03180KF,031209C,031H09G,03140Z0,061V47Y \| \| 061M07Y,061P4AY,051G4JY,051P47Y,041L4KK \| \| 041M0KS,03160Z5,041K4ZJ,041N0AM,031A0Z4 \| \| 316093,03120Z7,051N0AY,03150KJ,041L0KM \| \| 03130A8,051R4ZY,03120A9,03120Z3,061T4AY \| \| 03160J3,041M4ZM,03140JB,041M0JL,03150KF \| \| 051G4AY,03120AF,041L0JS,041M4AP,051P0AY \| \| 041M4ZS,041L4KN,03130A3,061R4ZY,051S09Y \| \| 03140Z5,051F4KY,051P0ZY,051F09Y,051T4KY \| \| 031C0Z4,05194KY,03150KK,051S0ZY,061C4JY \| \| 051L09Y,05180AY,313098,061P4JY,041L4AJ \| \| 041K0JL,041N4KM,03150Z2,03160K3,041L0ZN \| \| 051F4ZY,051R49Y,061T07Y,03170K0,051749Y \| \| 041L09N,051N0JY,041N0JM,03160KB,03120J6 \| \| 061S07Y,03120A2,041M0JP,03130J1,041M0AS \| \| 041K4AN,041N0KP,03130J8,03160JK,03120JJ \| \| 061P0JY,061D0AY,061D4AY,051Q4AY,051Q09Y \| \| 03120KF,03140K4,03120KK,051947Y,051F0JY \| \| 061R4KY,03160Z7,05180KY,03160K1,061T49Y \| \| 03130KF,041K4JH,03180ZF,061Q07Y,061G49Y \| \| 041N4AL,03180AF,313092,314091,03150K6 \| \| 03150J9,051H0ZY,03120J2,314092,03150K4 \| \| 041N0AP,03150Z0,051L47Y,051N4ZY,03130ZF \| \| 03140J5,03140K3,051H49Y,03160ZB,041K0KN \| \| 031309B,041M0AP,041L4AH,041L49M,041L4KL \| \| 03140J0,041L0AK,051B0JY,061C4AY,041K0ZP \| \| 03150A0,05190ZY,061R09Y,051R4KY,03150JD \| \| 041K49N,061F0ZY,061D49Y,041L4ZM,041N0ZS \| \| 061349Y,051F4AY,03130Z7,041L49S,041K4AS \| \| 03140KF,03160A4,041K4KL,031309F,03130J2 \| \| 051L0ZY,313094,051H4KY,051909Y,06130JY \| \| 041M09M,051B4JY,03120K2,051T0KY,03130J0 \| \| 031509K,041N4AS,041N0AQ,03160Z8,061H4ZY \| \| 061Q4ZY,041M09P,313095,041L49Q,061R07Y \| \| 315098,03140K7,05184ZY,03180JF,051H0AY \| \| 041K09H,061S0AY,051D07Y,041L4JK,051M4JY \| \| 041L0ZK,051C0AY,041N4AM,061Q0AY,031409D \| \| 051B0AY,051D0ZY,03150Z9,041L4AN,051H4AY \| \| 061P4KY,03160JJ,031409C,041K4AM,03150A1 \| \| 031709D,03130JJ,03160KJ,316097,05170ZY \| \| 041N0KQ,061F47Y,041L0AN,041K09N,I029 \| \| 03120JC,314098,051C0KY,051L49Y,061F4ZY \| \| 03120Z8,316099,041L4ZN,03190J3,03140AB \| \| 061G4ZY,061D0KY,041M0ZS,03120AC,314096 \| \| 061N07Y,061V0AY,031409J,051S4KY,041L4ZK \| \| 03160ZK,051F47Y,051B49Y,051T07Y,03140A7 \| \| 061F4JY,061P47Y,061M47Y,03130JK,051709Y \| \| 313093,03130JF,041L4ZJ,041L0JN,03150JC \| \| 041K0ZS,031409K,03140A4,041L4AK,03130J4 \| \| 03130Z9,041N49L,041L4ZL,05174ZY,041L0AP \| \| 051D0AY,03170KF,051Q0JY,051T47Y,041M4KP \| \| 03120JK,03180Z1,041L09L,03130K4,03140K2 \| \| 041L0AL,041L4JQ,061Q47Y,03160K7,061307Y \| \| 041N4ZQ,05174AY,041L0ZP,03140AD,031A0AF \| \| 03130J5,031J09G,051P4JY,03150A9,061V0ZY \| \| 051R09Y,051747Y,03130K9,061F4KY,051T4AY \| \| 03190ZF,031B0J3,03170K3,03150Z8,03120J7 \| \| 041N4JM,041N09S,061H09Y,03170J3,041K49J \| \| 03170AF,03150AC,041L0KN,03140J6,041K4ZK \| \| 03130JD,041N4KL,041K4JS,03160K8,061D0JY \| \| 03120Z5,041K4KP,041K0ZL,03140A5,314093 \| \| 051R0AY,051N4KY,06134AY,061G0AY,03150KB \| \| 061H4AY,031A094,051D47Y,041K0JM,03120K5 \| \| 031309J,041K0KH,03160KK,031609F,041K0ZM \| \| 041K49Q,03120AK,03190K3,061N4JY,051D4AY \| \| 051V4JY,03130AC,051P4AY,03160AK,031J0JG \| \| 051Q4ZY,051L4JY,051N49Y,041M49Q,03180Z4 \| \| 061S0ZY,051C4KY,041N0KM,051S0AY,041L4JJ \| \| 03130J7,312090,041K0JQ,051V4KY,041L0AH \| \| 041L4KH,041L0KQ,03170KD,03140ZD,061V49Y \| \| 312094,051G0ZY,041M4ZL,061T4JY,03150A2 \| \| 03150K1,041K0JS,041K0AH,315094,041K4ZP \| \| 031709F,312097,03150J4,06130KY,041N0JS \| \| 041N4ZP,041K4JQ \| |
| Vascular | Vascular bypass and shunt, not heart | aorta-renal bypass | 39.24 | \| 410095,04100K4,04104A5,04104A4,04100Z4 \| \| --- \| \| 410495,04104Z4,04104K3,04104J3,04104A3 \| \| 04104Z3,04104J4,04100Z5,04100A5,04100J3 \| \| 04100A4,04100J5,04104K4,410093,04100K5 \| \| 04104J5,410494,04104Z5,04100J4,04100Z3 \| \| 04100K3,410493,04104K5,04100A3,410094 \| |
|  |  | intra-abdomin shunt nec | 39.26 | \| 041J49D,041J099,041E4KP,041C49B,041E4JD \| \| --- \| \| 041D0JC,410490,041F4KG,041C4K2,041C0Z5 \| \| 041J09B,041H49P,041F0J9,041E0KG,04140A3 \| \| 041C4K1,041J4ZF,04140Z4,041C0Z8,041E49B \| \| 041F4K9,041C4ZB,041J0KQ,041F49Q,041H0ZP \| \| 041D4A4,041H49D,041D0Z3,041C0Z4,041D0J0 \| \| 041E4ZP,041E0ZP,041D4A8,041C0Z7,041D0Z8 \| \| 041H49F,041C0KC,041C0ZQ,041C4J2,04104A2 \| \| 041C4A6,041D0K1,041E0KD,041F0JB,041J0AD \| \| 041C4J4,041F4KF,041D09D,041D4K5,041E49C \| \| 041C09C,041E0AQ,041E4JG,041F4ZD,041D4A6 \| \| 041C4JG,041E09F,041D0K9,041J4KQ,04140J3 \| \| 041F0KP,041D0A4,041C4A2,04140K5,041J0AG \| \| 041D4K8,041D094,041F0AG,041C0Z3,041D49R \| \| 041F4ZF,041D4Z0,041F4KQ,041H4JF,041D0AD \| \| 041H4KP,041H4J9,041E0KC,041D4JC,04100K0 \| \| 04144A3,041E4JQ,041F49C,041D4A9,041C0J3 \| \| 041F4KD,041E0JF,041C0KD,041H4AG,041C49C \| \| 041F4JF,041E4JP,041C0A7,041J0KB,041F099 \| \| 041J09D,041D4AB,041C0Z9,041D4Z8,041D0Z6 \| \| 041D4K7,041D4Z7,041J49F,041H4ZQ,04104K2 \| \| 041H4KC,041D0JB,041F0ZB,041C4Z6,041C09R \| \| 041C4A3,041C0ZR,041C0Z6,041J4AD,041D0AG \| \| 041D4JB,041E0AG,041D09G,041C0A8,041D49D \| \| 041D4J5,414495,041H4AP,041C0K9,041D0J8 \| \| 041C4KF,041C09F,041J0ZF,041H0AQ,041F0Z9 \| \| 041J0KG,041C090,041C0A3,041C0KR,041J09G \| \| 041J4ZG,041D4K2,041E0KB,041D0KF,041F49P \| \| 041C49Q,041C4ZG,041J0KD,041H09G,041C0Z2 \| \| 041H4AD,041J0AC,041C4AD,410090,041D0ZF \| \| 041H0ZC,041H0A9,041D4Z2,041E4K9,041C0J1 \| \| 041H0KF,041J4ZQ,041D0A9,041F4KP,04144K5 \| \| 041F49D,041E4AD,041C0J9,041J4KP,041E0AB \| \| 041C4JC,041D4ZQ,041F09P,041C497,041D095 \| \| 041D0K7,041F09Q,041D4A0,041D0ZD,041C4J3 \| \| 041C4K0,041D0A5,041D0J2,041F0ZG,041E0ZD \| \| 041C4J7,041J4KC,041C4J6,041E4JB,041J49P \| \| 041C4A9,04100Z1,041D0J7,041J09Q,04100J0 \| \| 04104Z2,041C097,041D0Z1,041H4K9,041C4Z9 \| \| 041C0A6,041D0ZB,041D4AR,041C495,041D0J1 \| \| 041C091,041F0KF,041C0A1,041C0K6,041D4KR \| \| 041D49Q,041E4JF,041D498,04140A5,041C4Z2 \| \| 041D0A8,041J09F,041C4ZQ,04140J5,041D0Z2 \| \| 041C0AF,041D0A2,041E0JD,041D0A7,041D499 \| \| 04144J3,041C49R,041J4AB,041C0J7,041F09C \| \| 041E499,041D49F,041D4Z9,04140Z3,041C4JB \| \| 041D4KC,041C09D,04100K2,041C09G,041C4JR \| \| 041C496,041F0KD,04104K1,041J0ZD,041D0K3 \| \| 041F0AC,041H0J9,041H0KB,041E09Q,041H4ZC \| \| 041D49G,041H4JP,041H4KF,041C4Z3,041C0A5 \| \| 041D4J8,041D0K0,041E49F,041C4KD,041D09C \| \| 041J4ZD,041D497,04104J2,041E0KF,041C49F \| \| 041C4K5,041E4A9,041C0KB,04144A5,041D4A3 \| \| 041E4AP,041D4ZR,041E0ZC,041H099,041D4A1 \| \| 04104J0,041H4JQ,041C0K8,041F4AB,041C499 \| \| 04140Z5,041D0KC,041D0AR,041D0A0,041E4ZG \| \| 04100K1,041C0AR,041C0ZG,041E49G,041C4Z1 \| \| 041D4J1,041D0A3,041E0JP,041J0AP,041C490 \| \| 041C4A4,041F4JP,041D4J6,041D0Z7,041E4AF \| \| 04140A4,041C4ZD,041H4ZF,041C0Z0,041D4ZD \| \| 041F0ZQ,041H0JG,041E0JC,041E49P,041D0JR \| \| 041J0KF,041D4JR,041C0K2,041D4K0,04144K3 \| \| 041F09B,041C4J5,041D096,041C094,041H4Z9 \| \| 041J0ZC,041C498,041D0ZG,041C492,041C0J2 \| \| 04140J4,041E4KB,041F09F,041D0JQ,041E4KD \| \| 041H4AF,041J4AC,041H499,041F4AG,041D4Z6 \| \| 041C4KG,041F0KB,041F0AD,041C0ZC,041C4AG \| \| 041J09P,041F4ZQ,041F4JB,041C4K7,041D4K9 \| \| 041D4KF,041F0JG,041D493,041E4KF,041E09B \| \| 041C0K5,041J0AB,041F0AB,041H4JC,041D0AF \| \| 041D4JG,041H0Z9,041C0JG,041J4Z9,041E4AB \| \| 414493,041C4K8,041D0AC,041C4A1,041D4AC \| \| 414494,041E4AC,041D4AD,041D0K2,041D492 \| \| 041D0ZQ,04144J4,041C0J6,041F0JP,041H0AG \| \| 041C4K3,041E09C,041J49B,041J4JQ,041C098 \| \| 041H4ZB,041E0A9,041C0AQ,041H09C,041F0ZD \| \| 041D4KQ,041J4KG,041J49Q,041D4J9,041J4KF \| \| 041H4ZD,041J4ZC,041D4AQ,041H0ZD,04144J5 \| \| 041H4KG,041D4A7,041F0JF,041E0Z9,04144A4 \| \| 041D0K4,041D0Z5,04144K4,041D494,041E0ZG \| \| 041C0J5,041C0K7,041E4J9,041J49C,041C4KR \| \| 041J09C,041C4AR,041J0A9,041C0J8,041C0JF \| \| 041C4J8,041H0ZG,041D0A6,041C49D,041H4JB \| \| 414093,041F499,041F4JG,041H0AB,041D0J5 \| \| 041C4JD,041E4ZF,041C099,041D09R,414094 \| \| 041E4JC,041H09F,041H09B,041C4J0,041D0Z0 \| \| 041E4ZD,041C0A4,041F4ZP,041C0JR,041C4JQ \| \| 041E4AQ,041H4JG,041C4K4,041D491,041D4K6 \| \| 041C0AB,041D4J7,041D4K3,041C093,04140K3 \| \| 04104A1,041C0J0,041D4ZB,041D09F,04144Z3 \| \| 041D0ZC,041D4ZF,041E0ZQ,041C0K1,041J0ZQ \| \| 041J4JF,041H09P,041J4J9,041C09B,041J4ZP \| \| 041D4Z1,041D09Q,041D4ZC,041E4KC,041H0KC \| \| 041D4AF,041J4AG,041E0JB,041F4ZC,041C4J9 \| \| 041C493,041D0Z4,041D090,04140K4,041H49G \| \| 041C4Z7,041D0ZR,041C0JC,041D092,041D0JG \| \| 041J49G,041J4KD,041C494,04104J1,041F0K9 \| \| 041J4A9,041C4Z4,04144Z4,041C0K4,041H0AF \| \| 041D4A2,041E0ZF,041C4J1,04100Z0,041C4A7 \| \| 041C096,041D4Z5,04100Z2,041J4JB,041D091 \| \| 041H09Q,041H0JP,041D4AG,041J4JP,041E0JQ \| \| 041C0ZD,041H0K9,041C0KQ,041E49Q,041D0J3 \| \| 041D0K6,041J4K9,041F0AF,041J0J9,041C4A5 \| \| 041J0ZP,041F4AQ,041F0KQ,041C49G,041H49B \| \| 041F4J9,041E0K9,041F4A9,041C0JQ,04104K0 \| \| 041E49D,04104A0,041H0AP,041H0JB,041D0AB \| \| 041H0KQ,041F4AD,041J4JD,041J0K9,410091 \| \| 041D0KQ,041H0JF,041D4J3,041D0J4,04104Z1 \| \| 041H4AB,041D49C,041D4J0,041E4KQ,041E0J9 \| \| 041J4KB,041J0KP,041J4AQ,041C0ZF,041D0JD \| \| 041D0J6,041C4K9,041D09B,041C0K0,041C095 \| \| 041J4JG,041J0KC,04100J1,041C4AF,041C0Z1 \| \| 041F4JQ,041H49Q,041D4Z4,041C4AC,041H4JD \| \| 041C491,410092,041C0AG,041H0ZF,041H0AD \| \| 041C4ZF,041E0KQ,041J0ZG,410492,041D0A1 \| \| 041F0ZF,041H0JD,041F4KC,041C4Z8,041E4KG \| \| 041C0K3,041E4AG,041D4K1,041F4JD,041C4Z5 \| \| 041D4ZG,041D4J4,041F0KC,041F4Z9,041E09P \| \| 041F4KB,041F0JQ,041D0Z9,041C0ZB,04104Z0 \| \| 041F49G,041D0KR,041H4ZG,041C0JD,041C4AQ \| \| 041E4ZQ,041C4JF,041F0KG,04100A2,041D0K8 \| \| 041D097,041D4JD,041E4Z9,041D4KB,041D098 \| \| 041H4KQ,041J0JF,041C0AD,041H49C,041H0JC \| \| 041F0JC,041D0J9,041C4AB,041C4A8,041H0AC \| \| 041H4KB,041D496,04100A1,041C0A0,041J499 \| \| 041F49F,041F4ZG,041C4Z0,041F4ZB,041J0JP \| \| 041J4ZB,041J0Z9,041F4JC,041F09D,041C0KF \| \| 041D093,041E0JG,041C4KB,041D490,041C092 \| \| 041D0K5,041C4ZC,041E09G,041C4K6,041D4JF \| \| 041D099,041D49B,041F0ZP,414095,041H4ZP \| \| 041D0KD,041J0JQ,041F0ZC,041D0JF,041F4AF \| \| 041D495,04144Z5,041D4KG,041C0A2,041E0AF \| \| 041C0AC,041H0ZQ,041H4AC,041D4A5,410491 \| \| 041J0JC,041C0KG,041D0KG,041H0ZB,041E0AD \| \| 041F49B,041E4ZB,041D4KD,041D4JQ,041H0KP \| \| 041F4AC,041C4KQ,041J0AF,041D4K4,041E0KP \| \| 041H0KG,041J0JG,041J0ZB,041F4AP,041H09D \| \| 041D4Z3,041H0KD,041D0KB,041E09D,041C0A9 \| \| 041F09G,041F0A9,041F0JD,041J4AP,041E4ZC \| \| 041J0AQ,041C4KC,04100A0,041J4JC,041D4J2 \| \| 041C09Q,4.1E+100,041E0AC,041C4ZR,041J0JD \| \| 041E0ZB,041C0J4,041C4A0,041H0JQ,041F0AP \| \| 041H4AQ,041E0AP,041J0JB,04100J2,041C0JB \| \| 041J4AF,041H4A9,041F0AQ,041H4KD,041D0AQ \| |
| Neurology |  | extracran-intracr bypass | 39.28 | \| 031H0JJ,031N0AK,031H09J,031M0ZJ,031H0AJ \| \| --- \| \| 031J0AK,031K0ZJ,031S0ZG,031J0ZK,031H0KJ \| \| 031M0AJ,031L0JK,031S09G,031K0AJ,031S0AG \| \| 031T0AG,031N09K,031K0KJ,031H0ZJ,031T09G \| \| 031N0ZK,031M0KJ,031T0KG,031S0KG,031L0ZK \| \| 031J0KK,031L09K,031J0JK,031N0KK,031S0JG \| \| 031M0JJ,031T0ZG,031K0JJ,031L0AK,031T0JG \| \| 031M09J,031N0JK,031L0KK,031K09J,031J09K \| |
|  | Neuro Vascular | intracran vessel incis | 38.01 | 05CL4ZZ,05CL0ZZ,03CG0ZZ |
|  |  | occlus intracran ves nec | 38.81 | \| 03LG3ZZ,05LL0CZ,05LL4ZZ,05LL3CZ,05LL4DZ \| \| --- \| \| 05LL4CZ,05LL3DZ,05LL0ZZ,03LG0ZZ,03LG0CZ \| \| 03LG4ZZ,05LL3ZZ,05LL0DZ,03LG4CZ,03LG3CZ \| |
| Vascular | Embolectomy and endarterectomy of lower limbs | lower limb endarterect | 38.18 | \| 04CR0ZZ,04CY0ZZ,04CY3ZZ,04CS4ZZ,04CV0ZZ \| \| --- \| \| 04CP3ZZ,04CU0ZZ,04CW4ZZ,04CN4ZZ,04CR4ZZ \| \| 04CS0ZZ,04CK3ZZ,04CU4ZZ,04CK0ZZ,04CW3ZZ \| \| 04CP0ZZ,04CT4ZZ,04CT0ZZ,04CU3ZZ,04CS3ZZ \| \| 04CW0ZZ,04CK4ZZ,04CV4ZZ,04CV3ZZ,04CN0ZZ \| \| 04CT3ZZ,04CR3ZZ,04CQ4ZZ,04CQ3ZZ,04CP4ZZ \| \| 04CY4ZZ,04CM0ZZ,04CQ0ZZ,04CL3ZZ,04CL4ZZ \| \| 04CL0ZZ,04CM3ZZ,04CN3ZZ,04CM4ZZ \| |
|  | Emboletomy of Central Vessels | endarterectomy of aorta | 38.14 | \| 02CW0ZZ,04C03ZZ,02CW4ZZ,04C04ZZ,04C00ZZ \| \| --- \| \| 02CW3ZZ \| |
|  |  | thoracic endarterectomy | 38.15 | \| 03C04ZZ,03C14ZZ,02CQ0ZZ,03C40ZZ,02CQ4ZZ \| \| --- \| \| 02CT4ZZ,03C33ZZ,03C34ZZ,02CP3ZZ,02CV3ZZ \| \| 02CV4ZZ,03C30ZZ,02CR4ZZ,03C44ZZ,02CR3ZZ \| \| 03C03ZZ,02CS3ZZ,02CS4ZZ,03C10ZZ,03C00ZZ \| \| 03C43ZZ,02CP0ZZ,03C24ZZ,02CT0ZZ,02CS0ZZ \| \| 03C20ZZ,02CR0ZZ,02CQ3ZZ,03C23ZZ,02CP4ZZ \| \| 02CV0ZZ,03C13ZZ,02CT3ZZ \| |
|  |  | abdominal endarterectomy | 38.16 | \| 04CF0ZZ,04C44ZZ,04C60ZZ,04CH4ZZ,04C43ZZ \| \| --- \| \| 04CC4ZZ,04CF3ZZ,04C10ZZ,04CA4ZZ,04CA3ZZ \| \| 04CE3ZZ,04CJ3ZZ,04C80ZZ,04C24ZZ,04CB3ZZ \| \| 04CC0ZZ,04C14ZZ,04C84ZZ,04C83ZZ,04CD4ZZ \| \| 04C53ZZ,04CD3ZZ,04C54ZZ,04C34ZZ,04CF4ZZ \| \| 04CE4ZZ,04C74ZZ,04C30ZZ,04CB0ZZ,04CJ4ZZ \| \| 04C20ZZ,04C33ZZ,04CH3ZZ,04C94ZZ,04C50ZZ \| \| 04CA0ZZ,04C64ZZ,04C63ZZ,04CJ0ZZ,04C73ZZ \| \| 04C70ZZ,04CC3ZZ,04C40ZZ,04C13ZZ,04C90ZZ \| \| 04CH0ZZ,04C23ZZ,04CB4ZZ,04CE0ZZ,04C93ZZ \| \| 04CD0ZZ \| |
|  | Other Major Vasc Repair/Intervention | incision of aorta | 38.04 | \| 04C04ZZ,02CW0ZZ,04C00ZZ,02CW3ZZ,02CW4ZZ \| \| --- \| \| 04C03ZZ \| |
|  |  | thoracic vessel inc nec | 38.05 | \| 03C30ZZ,02CT3ZZ,03C44ZZ,03C43ZZ,05C03ZZ \| \| --- \| \| 05C43ZZ,02HS4DZ,05C40ZZ,02HS42Z,05C34ZZ \| \| 05C30ZZ,05C64ZZ,02HT3DZ,03C23ZZ,03C20ZZ \| \| 05C00ZZ,02CQ3ZZ,02CP0ZZ,03C04ZZ,03C00ZZ \| \| 02HR4DZ,02CR3ZZ,02HQ0DZ,02CV0ZZ,02HT0DZ \| \| 02CP3ZZ,03C33ZZ,02HT42Z,02HW4DZ,02HS32Z \| \| 02CS3ZZ,02HS0DZ,02CQ0ZZ,03C03ZZ,02HW02Z \| \| 02HP3DZ,02CT4ZZ,05C54ZZ,05C04ZZ,03C14ZZ \| \| 02HP4DZ,02CV4ZZ,02HS3DZ,02HW42Z,02CR0ZZ \| \| 02HQ3DZ,05C14ZZ,03C13ZZ,02HS02Z,05C44ZZ \| \| 03C40ZZ,02HW3DZ,02HP0DZ,03C24ZZ,05C13ZZ \| \| 03C10ZZ,02HQ4DZ,02CV3ZZ,02HR0DZ,02HT4DZ \| \| 02HW0DZ,05C60ZZ,03C34ZZ,05C33ZZ,02CR4ZZ \| \| 02CP4ZZ,02CT0ZZ,02HT02Z,02HR3DZ,05C50ZZ \| \| 05C10ZZ,02CQ4ZZ,05C53ZZ,02HT32Z,05C63ZZ \| \| 02CS0ZZ,02HW32Z,02CS4ZZ \| |
|  |  | abdomen artery incision | 38.06 | \| 04C54ZZ,04C13ZZ,04CE0ZZ,04C10ZZ,04CH0ZZ \| \| --- \| \| 04C30ZZ,04CH4ZZ,04CJ3ZZ,04CE4ZZ,04C84ZZ \| \| 04CJ0ZZ,04C53ZZ,04C24ZZ,04C63ZZ,04C83ZZ \| \| 04CA3ZZ,04CC4ZZ,04C50ZZ,04C74ZZ,04C44ZZ \| \| 04C34ZZ,04C60ZZ,04CF3ZZ,04C43ZZ,04C64ZZ \| \| 04C20ZZ,04CD4ZZ,04CF0ZZ,04CC0ZZ,04C14ZZ \| \| 04CD3ZZ,04C40ZZ,04CH3ZZ,04CJ4ZZ,04C73ZZ \| \| 04CB0ZZ,04C94ZZ,04CE3ZZ,04C93ZZ,04C33ZZ \| \| 04CA0ZZ,04C70ZZ,04CA4ZZ,04CB3ZZ,04CC3ZZ \| \| 04C90ZZ,04C80ZZ,04C23ZZ,04CB4ZZ,04CF4ZZ \| \| 04CD0ZZ \| |
|  |  | abdominal vein incision | 38.07 | \| 06C04ZZ,06C54ZZ,06C70ZZ,06C03ZZ,06C43ZZ \| \| --- \| \| 06CH3ZZ,06CJ0ZZ,06CH0ZZ,06C84ZZ,06C60ZZ \| \| 06C83ZZ,06C63ZZ,06CB3ZZ,06CJ3ZZ,06C10ZZ \| \| 06C13ZZ,06CF3ZZ,06C40ZZ,06C50ZZ,06CG0ZZ \| \| 06CD4ZZ,06C73ZZ,06C80ZZ,06CC3ZZ,06C94ZZ \| \| 06CG3ZZ,06CD3ZZ,06CH4ZZ,06C53ZZ,06C00ZZ \| \| 06CF4ZZ,06C64ZZ,06CD0ZZ,06CJ4ZZ,06C44ZZ \| \| 06CB4ZZ,06CC0ZZ,06C93ZZ,06CF0ZZ,06C24ZZ \| \| 06CG4ZZ,06C90ZZ,06C74ZZ,06C23ZZ,06CB0ZZ \| \| 06C14ZZ,06C20ZZ,06CC4ZZ \| |
|  |  | thor vessel resect/anast | 38.35 | \| 05B60ZZ,03B34ZZ,05B04ZZ,02BP0ZZ,03B24ZZ \| \| --- \| \| 03B14ZZ,02BV0ZZ,02BS0ZZ,03B44ZZ,03B30ZZ \| \| 05B40ZZ,02BT4ZZ,05B30ZZ,03B00ZZ,05B10ZZ \| \| 02BS4ZZ,05B00ZZ,03B40ZZ,05B14ZZ,02BT0ZZ \| \| 03B10ZZ,05B64ZZ,02BQ4ZZ,05B34ZZ,03B04ZZ \| \| 03B20ZZ,05B44ZZ,02BR4ZZ,02BP4ZZ,05B54ZZ \| \| 02BV4ZZ,02BQ0ZZ,02BR0ZZ,05B50ZZ \| |
|  |  | abd vessel resect/anast | 38.36 | \| 04B74ZZ,04B60ZZ,04BE0ZZ,04BC0ZZ,04BH0ZZ \| \| --- \| \| 04B14ZZ,04B80ZZ,04B30ZZ,04BJ4ZZ,04BA0ZZ \| \| 04BD0ZZ,04BF0ZZ,04B40ZZ,04BB4ZZ,04BF4ZZ \| \| 04BE4ZZ,04BC4ZZ,04B50ZZ,04B34ZZ,04B94ZZ \| \| 04B54ZZ,04BA4ZZ,04B24ZZ,04B44ZZ,04B10ZZ \| \| 04B90ZZ,04BJ0ZZ,04BD4ZZ,04B64ZZ,04B20ZZ \| \| 04B84ZZ,04BB0ZZ,04BH4ZZ,04B70ZZ \| |
|  |  | resect thorac ves w repl | 38.45 | \| 05R10KZ,02RV4JZ,05R40KZ,05R54JZ,05R607Z \| \| --- \| \| 02RT48Z,02RQ0KZ,03R20KZ,02RV0KZ,02RS4KZ \| \| 02RQ08Z,02RR0JZ,05R10JZ,05R007Z,03R00KZ \| \| 05R14JZ,05R40JZ,03R00JZ,03R10JZ,02RR4KZ \| \| 05R34JZ,05R60JZ,02RP08Z,03R44KZ,02RV07Z \| \| 05R30JZ,02RR07Z,02RQ4JZ,03R20JZ,05R347Z \| \| 05R407Z,02RV48Z,05R547Z,02RT0JZ,02RQ0JZ \| \| 02RQ48Z,02RV0JZ,03R24JZ,03R24KZ,02RW4KZ \| \| 03R40JZ,02RT4KZ,05R507Z,05R60KZ,03R207Z \| \| 02RR0KZ,02RQ4KZ,02RS0KZ,03R34KZ,02RS0JZ \| \| 03R04JZ,05R64JZ,05R04KZ,05R30KZ,02RR4JZ \| \| 05R00KZ,02RS47Z,02RR08Z,02RP0KZ,05R44JZ \| \| 05R64KZ,05R50KZ,02RT08Z,02RT07Z,03R447Z \| \| 05R44KZ,05R14KZ,03R247Z,03R347Z,05R54KZ \| \| 02RP07Z,05R50JZ,03R14JZ,03R30JZ,03R30KZ \| \| 02RW4JZ,05R307Z,02RW07Z,02RS08Z,02RW0KZ \| \| 02RR47Z,02RS4JZ,02RV47Z,03R04KZ,02RT47Z \| \| 03R007Z,05R34KZ,02RV08Z,05R147Z,02RP4KZ \| \| 02RT4JZ,05R04JZ,02RT0KZ,02RS48Z,02RW0JZ \| \| 02RP0JZ,02RP48Z,02RW08Z,03R107Z,05R447Z \| \| 05R047Z,02RV4KZ,05R00JZ,03R047Z,02RQ07Z \| \| 03R307Z,03R44JZ,03R10KZ,02RP47Z,02RQ47Z \| \| 02RW47Z,02RR48Z,02RW48Z,02RP4JZ,05R107Z \| \| 05R647Z,03R147Z,03R34JZ,03R14KZ,03R407Z \| \| 02RS07Z,03R40KZ \| |
|  |  | abd artery resec w repla | 38.46 | \| 04RH07Z,04RB47Z,04RC4KZ,04R747Z,04R90JZ \| \| --- \| \| 04RC4JZ,04RH4KZ,04R14JZ,04RJ47Z,04R64KZ \| \| 04RJ0KZ,04R70JZ,04R407Z,04R74JZ,04RE0JZ \| \| 04R847Z,04R20JZ,04R34JZ,04RF0JZ,04RF0KZ \| \| 04RD0KZ,04R207Z,04R44KZ,04RB0JZ,04RA0KZ \| \| 04RE4JZ,04R10JZ,04RA07Z,04R107Z,04RA47Z \| \| 04R147Z,04R40KZ,04R54KZ,04R74KZ,04RJ0JZ \| \| 04R90KZ,04RC47Z,04R607Z,04RE07Z,04RE4KZ \| \| 04RH0KZ,04RH0JZ,04RC07Z,04RH4JZ,04RE0KZ \| \| 04R50JZ,04RE47Z,04RF47Z,04R94KZ,04R54JZ \| \| 04R947Z,04R707Z,04R60KZ,04RJ4KZ,04RD47Z \| \| 04R60JZ,04R247Z,04R14KZ,04R30JZ,04RF07Z \| \| 04R447Z,04RD07Z,04RA0JZ,04RH47Z,04R40JZ \| \| 04R20KZ,04RD4JZ,04R94JZ,04RA4KZ,04R50KZ \| \| 04RB0KZ,04R507Z,04R24KZ,04R347Z,04R84JZ \| \| 04R84KZ,04RC0KZ,04RD0JZ,04R547Z,04R64JZ \| \| 04R307Z,04RB4KZ,04RC0JZ,04RB4JZ,04R70KZ \| \| 04R80KZ,04R647Z,04R80JZ,04R807Z,04RD4KZ \| \| 04R44JZ,04RJ07Z,04R907Z,04RB07Z,04RJ4JZ \| \| 04R30KZ,04R10KZ,04RA4JZ,04R24JZ,04RF4KZ \| \| 04RF4JZ,04R34KZ \| |
|  |  | thoracic vessel excision | 38.65 | \| 05503ZZ,03B04ZZ,05560ZZ,05543ZZ,05544ZZ \| \| --- \| \| 05553ZZ,02BP4ZZ,03504ZZ,05B64ZZ,03B13ZZ \| \| 02BR4ZZ,05B13ZZ,05B14ZZ,025T0ZZ,05510ZZ \| \| 02BT0ZZ,05B54ZZ,02BV0ZZ,03500ZZ,025T3ZZ \| \| 02BS3ZZ,025V4ZZ,05B43ZZ,025S0ZZ,025W0ZZ \| \| 03534ZZ,03523ZZ,05554ZZ,03B23ZZ,05533ZZ \| \| 03B30ZZ,025P3ZZ,05530ZZ,02BS4ZZ,05B30ZZ \| \| 025Q0ZZ,05550ZZ,02BS0ZZ,025V0ZZ,05564ZZ \| \| 02BV4ZZ,025P0ZZ,05B04ZZ,03B20ZZ,025T4ZZ \| \| 02BV3ZZ,025Q4ZZ,03503ZZ,03544ZZ,03510ZZ \| \| 025S4ZZ,025R0ZZ,05B63ZZ,03B33ZZ,05B10ZZ \| \| 03530ZZ,03540ZZ,05540ZZ,025W3ZZ,05B03ZZ \| \| 05B53ZZ,03B14ZZ,03B34ZZ,03514ZZ,05B44ZZ \| \| 02BQ4ZZ,03B43ZZ,03520ZZ,02BT4ZZ,025V3ZZ \| \| 025R4ZZ,03B44ZZ,03524ZZ,02BQ3ZZ,05B33ZZ \| \| 02BR0ZZ,025P4ZZ,025Q3ZZ,05500ZZ,03B40ZZ \| \| 03B10ZZ,02BR3ZZ,05B50ZZ,03B00ZZ,05B34ZZ \| \| 03513ZZ,025R3ZZ,05B60ZZ,025W4ZZ,05504ZZ \| \| 05534ZZ,02BP0ZZ,05563ZZ,05B00ZZ,03543ZZ \| \| 025S3ZZ,05513ZZ,02BQ0ZZ,05514ZZ,02BT3ZZ \| \| 03B24ZZ,05B40ZZ,03533ZZ,02BP3ZZ,02BW3ZZ \| \| 03B03ZZ \| |
|  |  | vasc proc revision nec | 39.49 | \| 05CY0ZZ,06CY3ZZ,04QY0ZZ,03CY3ZZ,06QY0ZZ \| \| --- \| \| 03CY0ZZ,05CY4ZZ,05QY3ZZ,05QY4ZZ,05CY3ZZ \| \| 04QY4ZZ,04QY3ZZ,06CY4ZZ,03QY4ZZ,04CY0ZZ \| \| 06CY0ZZ,03QY0ZZ,06QY3ZZ,03QY3ZZ,05QY0ZZ \| \| 03CY4ZZ,04CY4ZZ,04CY3ZZ,06QY4ZZ \| |
|  |  | aneurysm repair nec | 39.52 | \| 041J49D,041J099,041E4KP,041C49B,041E4JD \| \| --- \| \| 041D0JC,410490,041F4KG,041C4K2,041C0Z5 \| \| 041J09B,041H49P,041F0J9,041E0KG,04140A3 \| \| 041C4K1,041J4ZF,04140Z4,041C0Z8,041E49B \| \| 041F4K9,041C4ZB,041J0KQ,041F49Q,041H0ZP \| \| 041D4A4,041H49D,041D0Z3,041C0Z4,041D0J0 \| \| 041E4ZP,041E0ZP,041D4A8,041C0Z7,041D0Z8 \| \| 041H49F,041C0KC,041C0ZQ,041C4J2,04104A2 \| \| 041C4A6,041D0K1,041E0KD,041F0JB,041J0AD \| \| 041C4J4,041F4KF,041D09D,041D4K5,041E49C \| \| 041C09C,041E0AQ,041E4JG,041F4ZD,041D4A6 \| \| 041C4JG,041E09F,041D0K9,041J4KQ,04140J3 \| \| 041F0KP,041D0A4,041C4A2,04140K5,041J0AG \| \| 041D4K8,041D094,041F0AG,041C0Z3,041D49R \| \| 041F4ZF,041D4Z0,041F4KQ,041H4JF,041D0AD \| \| 041H4KP,041H4J9,041E0KC,041D4JC,04100K0 \| \| 04144A3,041E4JQ,041F49C,041D4A9,041C0J3 \| \| 041F4KD,041E0JF,041C0KD,041H4AG,041C49C \| \| 041F4JF,041E4JP,041C0A7,041J0KB,041F099 \| \| 041J09D,041D4AB,041C0Z9,041D4Z8,041D0Z6 \| \| 041D4K7,041D4Z7,041J49F,041H4ZQ,04104K2 \| \| 041H4KC,041D0JB,041F0ZB,041C4Z6,041C09R \| \| 041C4A3,041C0ZR,041C0Z6,041J4AD,041D0AG \| \| 041D4JB,041E0AG,041D09G,041C0A8,041D49D \| \| 041D4J5,414495,041H4AP,041C0K9,041D0J8 \| \| 041C4KF,041C09F,041J0ZF,041H0AQ,041F0Z9 \| \| 041J0KG,041C090,041C0A3,041C0KR,041J09G \| \| 041J4ZG,041D4K2,041E0KB,041D0KF,041F49P \| \| 041C49Q,041C4ZG,041J0KD,041H09G,041C0Z2 \| \| 041H4AD,041J0AC,041C4AD,410090,041D0ZF \| \| 041H0ZC,041H0A9,041D4Z2,041E4K9,041C0J1 \| \| 041H0KF,041J4ZQ,041D0A9,041F4KP,04144K5 \| \| 041F49D,041E4AD,041C0J9,041J4KP,041E0AB \| \| 041C4JC,041D4ZQ,041F09P,041C497,041D095 \| \| 041D0K7,041F09Q,041D4A0,041D0ZD,041C4J3 \| \| 041C4K0,041D0A5,041D0J2,041F0ZG,041E0ZD \| \| 041C4J7,041J4KC,041C4J6,041E4JB,041J49P \| \| 041C4A9,04100Z1,041D0J7,041J09Q,04100J0 \| \| 04104Z2,041C097,041D0Z1,041H4K9,041C4Z9 \| \| 041C0A6,041D0ZB,041D4AR,041C495,041D0J1 \| \| 041C091,041F0KF,041C0A1,041C0K6,041D4KR \| \| 041D49Q,041E4JF,041D498,04140A5,041C4Z2 \| \| 041D0A8,041J09F,041C4ZQ,04140J5,041D0Z2 \| \| 041C0AF,041D0A2,041E0JD,041D0A7,041D499 \| \| 04144J3,041C49R,041J4AB,041C0J7,041F09C \| \| 041E499,041D49F,041D4Z9,04140Z3,041C4JB \| \| 041D4KC,041C09D,04100K2,041C09G,041C4JR \| \| 041C496,041F0KD,04104K1,041J0ZD,041D0K3 \| \| 041F0AC,041H0J9,041H0KB,041E09Q,041H4ZC \| \| 041D49G,041H4JP,041H4KF,041C4Z3,041C0A5 \| \| 041D4J8,041D0K0,041E49F,041C4KD,041D09C \| \| 041J4ZD,041D497,04104J2,041E0KF,041C49F \| \| 041C4K5,041E4A9,041C0KB,04144A5,041D4A3 \| \| 041E4AP,041D4ZR,041E0ZC,041H099,041D4A1 \| \| 04104J0,041H4JQ,041C0K8,041F4AB,041C499 \| \| 04140Z5,041D0KC,041D0AR,041D0A0,041E4ZG \| \| 04100K1,041C0AR,041C0ZG,041E49G,041C4Z1 \| \| 041D4J1,041D0A3,041E0JP,041J0AP,041C490 \| \| 041C4A4,041F4JP,041D4J6,041D0Z7,041E4AF \| \| 04140A4,041C4ZD,041H4ZF,041C0Z0,041D4ZD \| \| 041F0ZQ,041H0JG,041E0JC,041E49P,041D0JR \| \| 041J0KF,041D4JR,041C0K2,041D4K0,04144K3 \| \| 041F09B,041C4J5,041D096,041C094,041H4Z9 \| \| 041J0ZC,041C498,041D0ZG,041C492,041C0J2 \| \| 04140J4,041E4KB,041F09F,041D0JQ,041E4KD \| \| 041H4AF,041J4AC,041H499,041F4AG,041D4Z6 \| \| 041C4KG,041F0KB,041F0AD,041C0ZC,041C4AG \| \| 041J09P,041F4ZQ,041F4JB,041C4K7,041D4K9 \| \| 041D4KF,041F0JG,041D493,041E4KF,041E09B \| \| 041C0K5,041J0AB,041F0AB,041H4JC,041D0AF \| \| 041D4JG,041H0Z9,041C0JG,041J4Z9,041E4AB \| \| 414493,041C4K8,041D0AC,041C4A1,041D4AC \| \| 414494,041E4AC,041D4AD,041D0K2,041D492 \| \| 041D0ZQ,04144J4,041C0J6,041F0JP,041H0AG \| \| 041C4K3,041E09C,041J49B,041J4JQ,041C098 \| \| 041H4ZB,041E0A9,041C0AQ,041H09C,041F0ZD \| \| 041D4KQ,041J4KG,041J49Q,041D4J9,041J4KF \| \| 041H4ZD,041J4ZC,041D4AQ,041H0ZD,04144J5 \| \| 041H4KG,041D4A7,041F0JF,041E0Z9,04144A4 \| \| 041D0K4,041D0Z5,04144K4,041D494,041E0ZG \| \| 041C0J5,041C0K7,041E4J9,041J49C,041C4KR \| \| 041J09C,041C4AR,041J0A9,041C0J8,041C0JF \| \| 041C4J8,041H0ZG,041D0A6,041C49D,041H4JB \| \| 414093,041F499,041F4JG,041H0AB,041D0J5 \| \| 041C4JD,041E4ZF,041C099,041D09R,414094 \| \| 041E4JC,041H09F,041H09B,041C4J0,041D0Z0 \| \| 041E4ZD,041C0A4,041F4ZP,041C0JR,041C4JQ \| \| 041E4AQ,041H4JG,041C4K4,041D491,041D4K6 \| \| 041C0AB,041D4J7,041D4K3,041C093,04140K3 \| \| 04104A1,041C0J0,041D4ZB,041D09F,04144Z3 \| \| 041D0ZC,041D4ZF,041E0ZQ,041C0K1,041J0ZQ \| \| 041J4JF,041H09P,041J4J9,041C09B,041J4ZP \| \| 041D4Z1,041D09Q,041D4ZC,041E4KC,041H0KC \| \| 041D4AF,041J4AG,041E0JB,041F4ZC,041C4J9 \| \| 041C493,041D0Z4,041D090,04140K4,041H49G \| \| 041C4Z7,041D0ZR,041C0JC,041D092,041D0JG \| \| 041J49G,041J4KD,041C494,04104J1,041F0K9 \| \| 041J4A9,041C4Z4,04144Z4,041C0K4,041H0AF \| \| 041D4A2,041E0ZF,041C4J1,04100Z0,041C4A7 \| \| 041C096,041D4Z5,04100Z2,041J4JB,041D091 \| \| 041H09Q,041H0JP,041D4AG,041J4JP,041E0JQ \| \| 041C0ZD,041H0K9,041C0KQ,041E49Q,041D0J3 \| \| 041D0K6,041J4K9,041F0AF,041J0J9,041C4A5 \| \| 041J0ZP,041F4AQ,041F0KQ,041C49G,041H49B \| \| 041F4J9,041E0K9,041F4A9,041C0JQ,04104K0 \| \| 041E49D,04104A0,041H0AP,041H0JB,041D0AB \| \| 041H0KQ,041F4AD,041J4JD,041J0K9,410091 \| \| 041D0KQ,041H0JF,041D4J3,041D0J4,04104Z1 \| \| 041H4AB,041D49C,041D4J0,041E4KQ,041E0J9 \| \| 041J4KB,041J0KP,041J4AQ,041C0ZF,041D0JD \| \| 041D0J6,041C4K9,041D09B,041C0K0,041C095 \| \| 041J4JG,041J0KC,04100J1,041C4AF,041C0Z1 \| \| 041F4JQ,041H49Q,041D4Z4,041C4AC,041H4JD \| \| 041C491,410092,041C0AG,041H0ZF,041H0AD \| \| 041C4ZF,041E0KQ,041J0ZG,410492,041D0A1 \| \| 041F0ZF,041H0JD,041F4KC,041C4Z8,041E4KG \| \| 041C0K3,041E4AG,041D4K1,041F4JD,041C4Z5 \| \| 041D4ZG,041D4J4,041F0KC,041F4Z9,041E09P \| \| 041F4KB,041F0JQ,041D0Z9,041C0ZB,04104Z0 \| \| 041F49G,041D0KR,041H4ZG,041C0JD,041C4AQ \| \| 041E4ZQ,041C4JF,041F0KG,04100A2,041D0K8 \| \| 041D097,041D4JD,041E4Z9,041D4KB,041D098 \| \| 041H4KQ,041J0JF,041C0AD,041H49C,041H0JC \| \| 041F0JC,041D0J9,041C4AB,041C4A8,041H0AC \| \| 041H4KB,041D496,04100A1,041C0A0,041J499 \| \| 041F49F,041F4ZG,041C4Z0,041F4ZB,041J0JP \| \| 041J4ZB,041J0Z9,041F4JC,041F09D,041C0KF \| \| 041D093,041E0JG,041C4KB,041D490,041C092 \| \| 041D0K5,041C4ZC,041E09G,041C4K6,041D4JF \| \| 041D099,041D49B,041F0ZP,414095,041H4ZP \| \| 041D0KD,041J0JQ,041F0ZC,041D0JF,041F4AF \| \| 041D495,04144Z5,041D4KG,041C0A2,041E0AF \| \| 041C0AC,041H0ZQ,041H4AC,041D4A5,410491 \| \| 041J0JC,041C0KG,041D0KG,041H0ZB,041E0AD \| \| 041F49B,041E4ZB,041D4KD,041D4JQ,041H0KP \| \| 041F4AC,041C4KQ,041J0AF,041D4K4,041E0KP \| \| 041H0KG,041J0JG,041J0ZB,041F4AP,041H09D \| \| 041D4Z3,041H0KD,041D0KB,041E09D,041C0A9 \| \| 041F09G,041F0A9,041F0JD,041J4AP,041E4ZC \| \| 041J0AQ,041C4KC,04100A0,041J4JC,041D4J2 \| \| 041C09Q,4.1E+100,041E0AC,041C4ZR,041J0JD \| \| 041E0ZB,041C0J4,041C4A0,041H0JQ,041F0AP \| \| 041H4AQ,041E0AP,041J0JB,04100J2,041C0JB \| \| 041J4AF,041H4A9,041F0AQ,041H4KD,041D0AQ \| |
| Neurology |  | extracran-intracr bypass | 39.28 | \| 031H0JJ,031N0AK,031H09J,031M0ZJ,031H0AJ \| \| --- \| \| 031J0AK,031K0ZJ,031S0ZG,031J0ZK,031H0KJ \| \| 031M0AJ,031L0JK,031S09G,031K0AJ,031S0AG \| \| 031T0AG,031N09K,031K0KJ,031H0ZJ,031T09G \| \| 031N0ZK,031M0KJ,031T0KG,031S0KG,031L0ZK \| \| 031J0KK,031L09K,031J0JK,031N0KK,031S0JG \| \| 031M0JJ,031T0ZG,031K0JJ,031L0AK,031T0JG \| \| 031M09J,031N0JK,031L0KK,031K09J,031J09K \| |
|  | Neuro Vascular | intracran vessel incis | 38.01 | 05CL4ZZ,05CL0ZZ,03CG0ZZ |
|  |  | occlus intracran ves nec | 38.81 | \| 03LG3ZZ,05LL0CZ,05LL4ZZ,05LL3CZ,05LL4DZ \| \| --- \| \| 05LL4CZ,05LL3DZ,05LL0ZZ,03LG0ZZ,03LG0CZ \| \| 03LG4ZZ,05LL3ZZ,05LL0DZ,03LG4CZ,03LG3CZ \| |
| Vascular | Embolectomy and endarterectomy of lower limbs | lower limb endarterect | 38.18 | \| 04CR0ZZ,04CY0ZZ,04CY3ZZ,04CS4ZZ,04CV0ZZ \| \| --- \| \| 04CP3ZZ,04CU0ZZ,04CW4ZZ,04CN4ZZ,04CR4ZZ \| \| 04CS0ZZ,04CK3ZZ,04CU4ZZ,04CK0ZZ,04CW3ZZ \| \| 04CP0ZZ,04CT4ZZ,04CT0ZZ,04CU3ZZ,04CS3ZZ \| \| 04CW0ZZ,04CK4ZZ,04CV4ZZ,04CV3ZZ,04CN0ZZ \| \| 04CT3ZZ,04CR3ZZ,04CQ4ZZ,04CQ3ZZ,04CP4ZZ \| \| 04CY4ZZ,04CM0ZZ,04CQ0ZZ,04CL3ZZ,04CL4ZZ \| \| 04CL0ZZ,04CM3ZZ,04CN3ZZ,04CM4ZZ \| |
|  | Emboletomy of Central Vessels | endarterectomy of aorta | 38.14 | \| 02CW0ZZ,04C03ZZ,02CW4ZZ,04C04ZZ,04C00ZZ \| \| --- \| \| 02CW3ZZ \| |
|  |  | thoracic endarterectomy | 38.15 | \| 03C04ZZ,03C14ZZ,02CQ0ZZ,03C40ZZ,02CQ4ZZ \| \| --- \| \| 02CT4ZZ,03C33ZZ,03C34ZZ,02CP3ZZ,02CV3ZZ \| \| 02CV4ZZ,03C30ZZ,02CR4ZZ,03C44ZZ,02CR3ZZ \| \| 03C03ZZ,02CS3ZZ,02CS4ZZ,03C10ZZ,03C00ZZ \| \| 03C43ZZ,02CP0ZZ,03C24ZZ,02CT0ZZ,02CS0ZZ \| \| 03C20ZZ,02CR0ZZ,02CQ3ZZ,03C23ZZ,02CP4ZZ \| \| 02CV0ZZ,03C13ZZ,02CT3ZZ \| |
|  |  | abdominal endarterectomy | 38.16 | \| 04CF0ZZ,04C44ZZ,04C60ZZ,04CH4ZZ,04C43ZZ \| \| --- \| \| 04CC4ZZ,04CF3ZZ,04C10ZZ,04CA4ZZ,04CA3ZZ \| \| 04CE3ZZ,04CJ3ZZ,04C80ZZ,04C24ZZ,04CB3ZZ \| \| 04CC0ZZ,04C14ZZ,04C84ZZ,04C83ZZ,04CD4ZZ \| \| 04C53ZZ,04CD3ZZ,04C54ZZ,04C34ZZ,04CF4ZZ \| \| 04CE4ZZ,04C74ZZ,04C30ZZ,04CB0ZZ,04CJ4ZZ \| \| 04C20ZZ,04C33ZZ,04CH3ZZ,04C94ZZ,04C50ZZ \| \| 04CA0ZZ,04C64ZZ,04C63ZZ,04CJ0ZZ,04C73ZZ \| \| 04C70ZZ,04CC3ZZ,04C40ZZ,04C13ZZ,04C90ZZ \| \| 04CH0ZZ,04C23ZZ,04CB4ZZ,04CE0ZZ,04C93ZZ \| \| 04CD0ZZ \| |
|  | Other Major Vasc Repair/Intervention | incision of aorta | 38.04 | \| 04C04ZZ,02CW0ZZ,04C00ZZ,02CW3ZZ,02CW4ZZ \| \| --- \| \| 04C03ZZ \| |
|  |  | thoracic vessel inc nec | 38.05 | \| 03C30ZZ,02CT3ZZ,03C44ZZ,03C43ZZ,05C03ZZ \| \| --- \| \| 05C43ZZ,02HS4DZ,05C40ZZ,02HS42Z,05C34ZZ \| \| 05C30ZZ,05C64ZZ,02HT3DZ,03C23ZZ,03C20ZZ \| \| 05C00ZZ,02CQ3ZZ,02CP0ZZ,03C04ZZ,03C00ZZ \| \| 02HR4DZ,02CR3ZZ,02HQ0DZ,02CV0ZZ,02HT0DZ \| \| 02CP3ZZ,03C33ZZ,02HT42Z,02HW4DZ,02HS32Z \| \| 02CS3ZZ,02HS0DZ,02CQ0ZZ,03C03ZZ,02HW02Z \| \| 02HP3DZ,02CT4ZZ,05C54ZZ,05C04ZZ,03C14ZZ \| \| 02HP4DZ,02CV4ZZ,02HS3DZ,02HW42Z,02CR0ZZ \| \| 02HQ3DZ,05C14ZZ,03C13ZZ,02HS02Z,05C44ZZ \| \| 03C40ZZ,02HW3DZ,02HP0DZ,03C24ZZ,05C13ZZ \| \| 03C10ZZ,02HQ4DZ,02CV3ZZ,02HR0DZ,02HT4DZ \| \| 02HW0DZ,05C60ZZ,03C34ZZ,05C33ZZ,02CR4ZZ \| \| 02CP4ZZ,02CT0ZZ,02HT02Z,02HR3DZ,05C50ZZ \| \| 05C10ZZ,02CQ4ZZ,05C53ZZ,02HT32Z,05C63ZZ \| \| 02CS0ZZ,02HW32Z,02CS4ZZ \| |
|  |  | abdomen artery incision | 38.06 | \| 04C54ZZ,04C13ZZ,04CE0ZZ,04C10ZZ,04CH0ZZ \| \| --- \| \| 04C30ZZ,04CH4ZZ,04CJ3ZZ,04CE4ZZ,04C84ZZ \| \| 04CJ0ZZ,04C53ZZ,04C24ZZ,04C63ZZ,04C83ZZ \| \| 04CA3ZZ,04CC4ZZ,04C50ZZ,04C74ZZ,04C44ZZ \| \| 04C34ZZ,04C60ZZ,04CF3ZZ,04C43ZZ,04C64ZZ \| \| 04C20ZZ,04CD4ZZ,04CF0ZZ,04CC0ZZ,04C14ZZ \| \| 04CD3ZZ,04C40ZZ,04CH3ZZ,04CJ4ZZ,04C73ZZ \| \| 04CB0ZZ,04C94ZZ,04CE3ZZ,04C93ZZ,04C33ZZ \| \| 04CA0ZZ,04C70ZZ,04CA4ZZ,04CB3ZZ,04CC3ZZ \| \| 04C90ZZ,04C80ZZ,04C23ZZ,04CB4ZZ,04CF4ZZ \| \| 04CD0ZZ \| |
|  |  | abdominal vein incision | 38.07 | \| 06C04ZZ,06C54ZZ,06C70ZZ,06C03ZZ,06C43ZZ \| \| --- \| \| 06CH3ZZ,06CJ0ZZ,06CH0ZZ,06C84ZZ,06C60ZZ \| \| 06C83ZZ,06C63ZZ,06CB3ZZ,06CJ3ZZ,06C10ZZ \| \| 06C13ZZ,06CF3ZZ,06C40ZZ,06C50ZZ,06CG0ZZ \| \| 06CD4ZZ,06C73ZZ,06C80ZZ,06CC3ZZ,06C94ZZ \| \| 06CG3ZZ,06CD3ZZ,06CH4ZZ,06C53ZZ,06C00ZZ \| \| 06CF4ZZ,06C64ZZ,06CD0ZZ,06CJ4ZZ,06C44ZZ \| \| 06CB4ZZ,06CC0ZZ,06C93ZZ,06CF0ZZ,06C24ZZ \| \| 06CG4ZZ,06C90ZZ,06C74ZZ,06C23ZZ,06CB0ZZ \| \| 06C14ZZ,06C20ZZ,06CC4ZZ \| |
|  |  | thor vessel resect/anast | 38.35 | \| 05B60ZZ,03B34ZZ,05B04ZZ,02BP0ZZ,03B24ZZ \| \| --- \| \| 03B14ZZ,02BV0ZZ,02BS0ZZ,03B44ZZ,03B30ZZ \| \| 05B40ZZ,02BT4ZZ,05B30ZZ,03B00ZZ,05B10ZZ \| \| 02BS4ZZ,05B00ZZ,03B40ZZ,05B14ZZ,02BT0ZZ \| \| 03B10ZZ,05B64ZZ,02BQ4ZZ,05B34ZZ,03B04ZZ \| \| 03B20ZZ,05B44ZZ,02BR4ZZ,02BP4ZZ,05B54ZZ \| \| 02BV4ZZ,02BQ0ZZ,02BR0ZZ,05B50ZZ \| |
|  |  | abd vessel resect/anast | 38.36 | \| 04B74ZZ,04B60ZZ,04BE0ZZ,04BC0ZZ,04BH0ZZ \| \| --- \| \| 04B14ZZ,04B80ZZ,04B30ZZ,04BJ4ZZ,04BA0ZZ \| \| 04BD0ZZ,04BF0ZZ,04B40ZZ,04BB4ZZ,04BF4ZZ \| \| 04BE4ZZ,04BC4ZZ,04B50ZZ,04B34ZZ,04B94ZZ \| \| 04B54ZZ,04BA4ZZ,04B24ZZ,04B44ZZ,04B10ZZ \| \| 04B90ZZ,04BJ0ZZ,04BD4ZZ,04B64ZZ,04B20ZZ \| \| 04B84ZZ,04BB0ZZ,04BH4ZZ,04B70ZZ \| |
|  |  | resect thorac ves w repl | 38.45 | \| 05R10KZ,02RV4JZ,05R40KZ,05R54JZ,05R607Z \| \| --- \| \| 02RT48Z,02RQ0KZ,03R20KZ,02RV0KZ,02RS4KZ \| \| 02RQ08Z,02RR0JZ,05R10JZ,05R007Z,03R00KZ \| \| 05R14JZ,05R40JZ,03R00JZ,03R10JZ,02RR4KZ \| \| 05R34JZ,05R60JZ,02RP08Z,03R44KZ,02RV07Z \| \| 05R30JZ,02RR07Z,02RQ4JZ,03R20JZ,05R347Z \| \| 05R407Z,02RV48Z,05R547Z,02RT0JZ,02RQ0JZ \| \| 02RQ48Z,02RV0JZ,03R24JZ,03R24KZ,02RW4KZ \| \| 03R40JZ,02RT4KZ,05R507Z,05R60KZ,03R207Z \| \| 02RR0KZ,02RQ4KZ,02RS0KZ,03R34KZ,02RS0JZ \| \| 03R04JZ,05R64JZ,05R04KZ,05R30KZ,02RR4JZ \| \| 05R00KZ,02RS47Z,02RR08Z,02RP0KZ,05R44JZ \| \| 05R64KZ,05R50KZ,02RT08Z,02RT07Z,03R447Z \| \| 05R44KZ,05R14KZ,03R247Z,03R347Z,05R54KZ \| \| 02RP07Z,05R50JZ,03R14JZ,03R30JZ,03R30KZ \| \| 02RW4JZ,05R307Z,02RW07Z,02RS08Z,02RW0KZ \| \| 02RR47Z,02RS4JZ,02RV47Z,03R04KZ,02RT47Z \| \| 03R007Z,05R34KZ,02RV08Z,05R147Z,02RP4KZ \| \| 02RT4JZ,05R04JZ,02RT0KZ,02RS48Z,02RW0JZ \| \| 02RP0JZ,02RP48Z,02RW08Z,03R107Z,05R447Z \| \| 05R047Z,02RV4KZ,05R00JZ,03R047Z,02RQ07Z \| \| 03R307Z,03R44JZ,03R10KZ,02RP47Z,02RQ47Z \| \| 02RW47Z,02RR48Z,02RW48Z,02RP4JZ,05R107Z \| \| 05R647Z,03R147Z,03R34JZ,03R14KZ,03R407Z \| \| 02RS07Z,03R40KZ \| |
|  |  | abd artery resec w repla | 38.46 | \| 04RH07Z,04RB47Z,04RC4KZ,04R747Z,04R90JZ \| \| --- \| \| 04RC4JZ,04RH4KZ,04R14JZ,04RJ47Z,04R64KZ \| \| 04RJ0KZ,04R70JZ,04R407Z,04R74JZ,04RE0JZ \| \| 04R847Z,04R20JZ,04R34JZ,04RF0JZ,04RF0KZ \| \| 04RD0KZ,04R207Z,04R44KZ,04RB0JZ,04RA0KZ \| \| 04RE4JZ,04R10JZ,04RA07Z,04R107Z,04RA47Z \| \| 04R147Z,04R40KZ,04R54KZ,04R74KZ,04RJ0JZ \| \| 04R90KZ,04RC47Z,04R607Z,04RE07Z,04RE4KZ \| \| 04RH0KZ,04RH0JZ,04RC07Z,04RH4JZ,04RE0KZ \| \| 04R50JZ,04RE47Z,04RF47Z,04R94KZ,04R54JZ \| \| 04R947Z,04R707Z,04R60KZ,04RJ4KZ,04RD47Z \| \| 04R60JZ,04R247Z,04R14KZ,04R30JZ,04RF07Z \| \| 04R447Z,04RD07Z,04RA0JZ,04RH47Z,04R40JZ \| \| 04R20KZ,04RD4JZ,04R94JZ,04RA4KZ,04R50KZ \| \| 04RB0KZ,04R507Z,04R24KZ,04R347Z,04R84JZ \| \| 04R84KZ,04RC0KZ,04RD0JZ,04R547Z,04R64JZ \| \| 04R307Z,04RB4KZ,04RC0JZ,04RB4JZ,04R70KZ \| \| 04R80KZ,04R647Z,04R80JZ,04R807Z,04RD4KZ \| \| 04R44JZ,04RJ07Z,04R907Z,04RB07Z,04RJ4JZ \| \| 04R30KZ,04R10KZ,04RA4JZ,04R24JZ,04RF4KZ \| \| 04RF4JZ,04R34KZ \| |
|  |  | thoracic vessel excision | 38.65 | \| 05503ZZ,03B04ZZ,05560ZZ,05543ZZ,05544ZZ \| \| --- \| \| 05553ZZ,02BP4ZZ,03504ZZ,05B64ZZ,03B13ZZ \| \| 02BR4ZZ,05B13ZZ,05B14ZZ,025T0ZZ,05510ZZ \| \| 02BT0ZZ,05B54ZZ,02BV0ZZ,03500ZZ,025T3ZZ \| \| 02BS3ZZ,025V4ZZ,05B43ZZ,025S0ZZ,025W0ZZ \| \| 03534ZZ,03523ZZ,05554ZZ,03B23ZZ,05533ZZ \| \| 03B30ZZ,025P3ZZ,05530ZZ,02BS4ZZ,05B30ZZ \| \| 025Q0ZZ,05550ZZ,02BS0ZZ,025V0ZZ,05564ZZ \| \| 02BV4ZZ,025P0ZZ,05B04ZZ,03B20ZZ,025T4ZZ \| \| 02BV3ZZ,025Q4ZZ,03503ZZ,03544ZZ,03510ZZ \| \| 025S4ZZ,025R0ZZ,05B63ZZ,03B33ZZ,05B10ZZ \| \| 03530ZZ,03540ZZ,05540ZZ,025W3ZZ,05B03ZZ \| \| 05B53ZZ,03B14ZZ,03B34ZZ,03514ZZ,05B44ZZ \| \| 02BQ4ZZ,03B43ZZ,03520ZZ,02BT4ZZ,025V3ZZ \| \| 025R4ZZ,03B44ZZ,03524ZZ,02BQ3ZZ,05B33ZZ \| \| 02BR0ZZ,025P4ZZ,025Q3ZZ,05500ZZ,03B40ZZ \| \| 03B10ZZ,02BR3ZZ,05B50ZZ,03B00ZZ,05B34ZZ \| \| 03513ZZ,025R3ZZ,05B60ZZ,025W4ZZ,05504ZZ \| \| 05534ZZ,02BP0ZZ,05563ZZ,05B00ZZ,03543ZZ \| \| 025S3ZZ,05513ZZ,02BQ0ZZ,05514ZZ,02BT3ZZ \| \| 03B24ZZ,05B40ZZ,03533ZZ,02BP3ZZ,02BW3ZZ \| \| 03B03ZZ \| |
|  |  | vasc proc revision nec | 39.49 | \| 05CY0ZZ,06CY3ZZ,04QY0ZZ,03CY3ZZ,06QY0ZZ \| \| --- \| \| 03CY0ZZ,05CY4ZZ,05QY3ZZ,05QY4ZZ,05CY3ZZ \| \| 04QY4ZZ,04QY3ZZ,06CY4ZZ,03QY4ZZ,04CY0ZZ \| \| 06CY0ZZ,03QY0ZZ,06QY3ZZ,03QY3ZZ,05QY0ZZ \| \| 03CY4ZZ,04CY4ZZ,04CY3ZZ,06QY4ZZ \| |
|  |  | aneurysm repair nec | 39.52 | \| 02VW0ZZ,06VS4ZZ,04VM4ZZ,06VG0ZZ,03V64ZZ \| \| --- \| \| 03VQ0ZZ,06VH0ZZ,05VG4ZZ,06VQ0ZZ,05VL4ZZ \| \| 06V50ZZ,03V20ZZ,05V10ZZ,06VC4ZZ,03VR0ZZ \| \| 02VS0ZZ,05VA0ZZ,04VK0ZZ,05V70ZZ,03V54ZZ \| \| 04V20ZZ,03V14ZZ,05V14ZZ,04VY4ZZ,05VS4ZZ \| \| 04V34ZZ,06V54ZZ,06VG4ZZ,06VR4ZZ,04VJ0ZZ \| \| 06VP0ZZ,03VN0ZZ,03VF0ZZ,05V60ZZ,05VV0ZZ \| \| 06V30ZZ,02VT0DZ,06VQ4ZZ,06VD4ZZ,03V24ZZ \| \| 03VL0ZZ,05VB4ZZ,04VD4ZZ,03VU4ZZ,05V90ZZ \| \| 05VY4ZZ,06V70ZZ,05VP4ZZ,03VC0ZZ,05VA4ZZ \| \| 04VN4ZZ,04V24ZZ,04VF4ZZ,05VM0ZZ,03V40ZZ \| \| 03V04ZZ,04V50ZZ,04V80ZZ,04VS0ZZ,06V64ZZ \| \| 06V44ZZ,04V00DZ,04VQ4ZZ,04VS4ZZ,06V60ZZ \| \| 05VD4ZZ,03V30ZZ,04VD0ZZ,06VY0ZZ,05VN4ZZ \| \| 04V54ZZ,03VY0ZZ,04VU0ZZ,03V70ZZ,03VM0ZZ \| \| 05VH4ZZ,06V24ZZ,04VT0ZZ,03VY4ZZ,05VM4ZZ \| \| 02VP0ZZ,06VM0ZZ,03V44ZZ,06VN4ZZ,04VU4ZZ \| \| 04VE4ZZ,04VJ4ZZ,05V44ZZ,04V04ZZ,05V50ZZ \| \| 06VY0DZ,06V20ZZ,04VK4ZZ,03VR4ZZ,04VL4ZZ \| \| 03V80ZZ,06VY4ZZ,06VR0ZZ,04VW4ZZ,04VY0ZZ \| \| 03VD4ZZ,03VJ0ZZ,02VT4ZZ,04V64ZZ,06V84ZZ \| \| 05VC0ZZ,05VT0ZZ,04VV4ZZ,04V44ZZ,05VL0ZZ \| \| 03VB4ZZ,03VA0ZZ,02VT4DZ,05VY0DZ,02VQ4ZZ \| \| 06V94ZZ,03VP4ZZ,03V34ZZ,03V00ZZ,06V14ZZ \| \| 02VW4DZ,04VB0ZZ,04VB4ZZ,04VC0ZZ,04VV0ZZ \| \| 05VR0ZZ,05VQ4ZZ,04V60ZZ,06V40ZZ,05V34ZZ \| \| 06VF0ZZ,02VS4ZZ,02VS4DZ,02VS0DZ,06VB4ZZ \| \| 03VC4ZZ,06VD0ZZ,03V90ZZ,06VB0ZZ,03VQ4ZZ \| \| 04V90ZZ,06V34ZZ,06VJ4ZZ,06VV0ZZ,05V00ZZ \| \| 05V64ZZ,04VL0ZZ,05VS0ZZ,03V10ZZ,04VE0ZZ \| \| 03V50ZZ,05VT4ZZ,03VS4ZZ,04VH4ZZ,04VP0ZZ \| \| 02VR0DZ,03VU0ZZ,06VM4ZZ,03VG0ZZ,05VP0ZZ \| \| 05VR4ZZ,03VF4ZZ,05VB0ZZ,03VT4ZZ,03VS0ZZ \| \| 04VC4ZZ,06V90ZZ,03VD0ZZ,05VV4ZZ,02VR4ZZ \| \| 04VA4ZZ,03V60ZZ,05V80ZZ,05V40ZZ,04V14ZZ \| \| 04VR4ZZ,03VN4ZZ,02VR4DZ,04VR0ZZ,06VT0ZZ \| \| 06V74ZZ,05VC4ZZ,06VS0ZZ,06VN0ZZ,02VQ4DZ \| \| 06VV4ZZ,02VP4DZ,03VV4ZZ,02VR4DT,06VP4ZZ \| \| 04VN0ZZ,06VT4ZZ,04VT4ZZ,05VN0ZZ,03VV0ZZ \| \| 04V10ZZ,02VR0ZT,04V70ZZ,04V00ZZ,06VY4DZ \| \| 04VA0ZZ,05V74ZZ,04VM0ZZ,02VW4ZZ,04V74ZZ \| \| 04VF0ZZ,06VJ0ZZ,03V74ZZ,06VF4ZZ,03VT0ZZ \| \| 03V84ZZ,04V94ZZ,05VQ0ZZ,04V40ZZ,06V80ZZ \| \| 04VQ0ZZ,02VR0ZZ,03VK0ZZ,03VB0ZZ,03VJ4ZZ \| \| 05VD0ZZ,03VG4ZZ,05VF4ZZ,05VY4DZ,04VH0ZZ \| \| 05VH0ZZ,05VG0ZZ,05V30ZZ,03VP0ZZ,03VL4ZZ \| \| 05VF0ZZ,06VC0ZZ,05V94ZZ,05V84ZZ,03VM4ZZ \| \| 03V94ZZ,02VQ0DZ,02VP0DZ,03VK4ZZ,02VQ0ZZ \| \| 02VR4ZT,05V04ZZ,05V54ZZ,06VH4ZZ,02VR0DT \| \| 02VP4ZZ,04VP4ZZ,06V10ZZ,04VW0ZZ,03VA4ZZ \| \| 02VT0ZZ,03VH0ZZ,03VH4ZZ,05VY0ZZ,04V84ZZ \| \| 04V30ZZ \| |
|  |  | rep vess w synth patch | 39.57 | \| 03UU4JZ,03UH0JZ,03U93JZ,03U70JZ,03UC4JZ \| \| --- \| \| 03U64JZ,04U70JZ,05U53JZ,06UT4JZ,04UJ3JZ \| \| 04UA4JZ,06UP4JZ,04UW0JZ,02UQ4JZ,03U24JZ \| \| 03UF4JZ,06UT3JZ,05U63JZ,03U00JZ,06UD3JZ \| \| 05U14JZ,05UQ4JZ,06U74JZ,03UB3JZ,04UY4JZ \| \| 04UH3JZ,06UV4JZ,06U90JZ,03UU3JZ,02UV0JZ \| \| 05UH0JZ,06U14JZ,04U74JZ,06UR4JZ,06US4JZ \| \| 04UJ4JZ,04U94JZ,04U00JZ,06UQ3JZ,06UV0JZ \| \| 06UN4JZ,06U04JZ,06UF4JZ,05UC4JZ,03U23JZ \| \| 03U20JZ,06UH4JZ,05UA4JZ,06UG0JZ,06UV3JZ \| \| 03UB4JZ,03UP0JZ,03UM0JZ,04UP0JZ,05U70JZ \| \| 04UM4JZ,04UK4JZ,03UL0JZ,04UF0JZ,06UY0JZ \| \| 03UR4JZ,04U63JZ,05US0JZ,05U60JZ,05UP4JZ \| \| 05UF4JZ,05U44JZ,03U60JZ,06UT0JZ,04UA0JZ \| \| 06U93JZ,03U43JZ,04UT3JZ,03U90JZ,06UN3JZ \| \| 03UY0JZ,04UU4JZ,04U64JZ,04U53JZ,06U54JZ \| \| 05U94JZ,05U54JZ,03U80JZ,03U84JZ,05U34JZ \| \| 04U34JZ,02US0JZ,05U84JZ,02UV3JZ,04UH4JZ \| \| 04U03JZ,03UH3JZ,05UY3JZ,05UF3JZ,03UT0JZ \| \| 06U73JZ,04U43JZ,03U40JZ,04UB3JZ,02UP3JZ \| \| 03UJ3JZ,06U13JZ,06UM3JZ,06U20JZ,03UC3JZ \| \| 06U80JZ,05UQ3JZ,06UG3JZ,03UC0JZ,05UL4JZ \| \| 04U13JZ,03UD3JZ,05UY0JZ,05UA0JZ,03UY3JZ \| \| 06U24JZ,04U14JZ,03UN0JZ,05U04JZ,03UJ4JZ \| \| 04UW4JZ,03U74JZ,06U84JZ,03UG0JZ,06UQ4JZ \| \| 03U54JZ,02UQ3JZ,03U03JZ,06U83JZ,04U24JZ \| \| 05UM4JZ,03UR0JZ,02US4JZ,03U33JZ,02UT4JZ \| \| 03UY4JZ,04UP4JZ,06UQ0JZ,04UL4JZ,05UB3JZ \| \| 03UH4JZ,05UD3JZ,05U10JZ,04UT0JZ,04UM0JZ \| \| 06UD0JZ,02UP0JZ,03UF0JZ,06UJ4JZ,05UQ0JZ \| \| 05UN3JZ,05UH4JZ,04UE0JZ,05U80JZ,06UM0JZ \| \| 02UT0JZ,04UR0JZ,03UA0JZ,05UV4JZ,06UD4JZ \| \| 04UU0JZ,05U00JZ,03UM4JZ,06U34JZ,04UU3JZ \| \| 04U40JZ,05UN0JZ,03UB0JZ,04UF4JZ,06UF3JZ \| \| 06U30JZ,04U83JZ,05UT4JZ,05U74JZ,06UC3JZ \| \| 04UK3JZ,03UP4JZ,04UN3JZ,02UV4JZ,04UF3JZ \| \| 06UN0JZ,04U20JZ,03UQ3JZ,05US4JZ,05UM3JZ \| \| 03U44JZ,04UH0JZ,04UQ0JZ,03UG4JZ,04US0JZ \| \| 04UN0JZ,06UH0JZ,04UY3JZ,06U94JZ,06U23JZ \| \| 05UD4JZ,05UG0JZ,06UB3JZ,03UR3JZ,03US4JZ \| \| 06U44JZ,06U10JZ,04UL0JZ,04U50JZ,03UT3JZ \| \| 06U40JZ,06U43JZ,03UL4JZ,06U60JZ,05UR0JZ \| \| 04UD0JZ,06UR3JZ,03UN4JZ,04U10JZ,04UR4JZ \| \| 05U43JZ,06UJ3JZ,06U50JZ,04UD4JZ,05U93JZ \| \| 02UR3JZ,04US4JZ,04UR3JZ,03U30JZ,05UR3JZ \| \| 05UT3JZ,04UD3JZ,03U14JZ,05U90JZ,04U84JZ \| \| 04U60JZ,04UV4JZ,06UY4JZ,04U73JZ,03UJ0JZ \| \| 04UC3JZ,04UB4JZ,03UD4JZ,03UL3JZ,04UE4JZ \| \| 05UV3JZ,04UM3JZ,04UJ0JZ,03U83JZ,04U90JZ \| \| 05U13JZ,04UQ4JZ,06UC4JZ,03UV3JZ,05U33JZ \| \| 05UR4JZ,04U54JZ,03UQ4JZ,03U13JZ,03U63JZ \| \| 03U94JZ,06UB0JZ,06UB4JZ,02UW0JZ,03UT4JZ \| \| 06U03JZ,05UN4JZ,06UM4JZ,06US3JZ,04UC4JZ \| \| 05UL0JZ,04UW3JZ,03U53JZ,03UF3JZ,04U33JZ \| \| 04UE3JZ,05UY4JZ,06U00JZ,04U30JZ,05UD0JZ \| \| 04U23JZ,05U73JZ,03UU0JZ,06UG4JZ,04UB0JZ \| \| 03UP3JZ,02UT3JZ,05U40JZ,04UK0JZ,04US3JZ \| \| 03U04JZ,03US3JZ,06UH3JZ,02US3JZ,05UF0JZ \| \| 03UN3JZ,05UV0JZ,03UQ0JZ,05UL3JZ,05UC3JZ \| \| 03UK4JZ,06UJ0JZ,04UP3JZ,04UT4JZ,02UP4JZ \| \| 03U73JZ,03UK0JZ,05U30JZ,04U80JZ,04U93JZ \| \| 06UC0JZ,03UV4JZ,05UH3JZ,04UN4JZ,04UL3JZ \| \| 05U50JZ,05UP0JZ,04UC0JZ,03UV0JZ,05U03JZ \| \| 06US0JZ,06U64JZ,06UF0JZ,05UB0JZ,05UC0JZ \| \| 06UP3JZ,05UP3JZ,06UP0JZ,05UA3JZ,06U33JZ \| \| 06UR0JZ,02UR4JZ,06UY3JZ,03UK3JZ,03U34JZ \| \| 04UQ3JZ,05UM0JZ,02UQ0JZ,05UT0JZ,04UV3JZ \| \| 06U70JZ,03UA3JZ,06U53JZ,05U64JZ,04UA3JZ \| \| 04U04JZ,03US0JZ,05U83JZ,03UG3JZ,03UD0JZ \| \| 03U10JZ,03UM3JZ,06U63JZ,04U44JZ,05UB4JZ \| \| 03UA4JZ,05UG3JZ,02UR0JZ,04UV0JZ,05US3JZ \| \| 03U50JZ,04UY0JZ,05UG4JZ \| |
| Cardiac | Ligation of Vessel | occlude aorta nec | 38.84 | \| 04L03ZZ,04L04ZZ,04L03DZ,04L04CZ,04L00ZZ \| \| --- \| \| 04L00DZ,04L04DZ,04L03CZ,04L00CZ \| |
| Thoracic |  | occlude thoracic ves nec | 38.85 | \| 05L63CZ,05L60DZ,03L00DZ,03L14DZ,03L30DZ \| \| --- \| \| 03L43DZ,05L33ZZ,03L23DZ,05L30DZ,03L00CZ \| \| 02VR3CZ,05L50DZ,03L34ZZ,02LT4ZZ,03L33DZ \| \| 02LT0CZ,03L33ZZ,02LS0DZ,05L54DZ,03L43CZ \| \| 05L30ZZ,05L10ZZ,02LR0ZT,03L03ZZ,05L63ZZ \| \| 03L23CZ,03L34CZ,05L14DZ,05L44DZ,03L13CZ \| \| 03L44CZ,02LT4CZ,05L34CZ,05L00DZ,03L14CZ \| \| 05L53CZ,05L33DZ,05L03CZ,02LR4CT,03L20DZ \| \| 05L34DZ,03L33CZ,03L44DZ,05L53DZ,05L13ZZ \| \| 02LT0ZZ,03L24ZZ,03L34DZ,05L04ZZ,03L23ZZ \| \| 05L50CZ,02LS0CZ,02LT3DZ,05L00CZ,02LS3DZ \| \| 02LR0CT,05L40CZ,03L20ZZ,03L00ZZ,05L43DZ \| \| 03L43ZZ,02LS0ZZ,02LT3ZZ,02LR4ZT,03L24CZ \| \| 05L60ZZ,03L04ZZ,03L30CZ,05L43CZ,05L44ZZ \| \| 02LS3CZ,02LS3ZZ,02VQ0CZ,05L54ZZ,05L13DZ \| \| 02LR3CT,02LS4CZ,03L10ZZ,03L04DZ,03L13DZ \| \| 03L20CZ,05L14ZZ,05L13CZ,03L44ZZ,03L40DZ \| \| 02LT0DZ,03L10CZ,05L03ZZ,05L64CZ,03L03CZ \| \| 05L30CZ,02LS4ZZ,05L04CZ,05L44CZ,03L40CZ \| \| 05L34ZZ,05L40DZ,05L33CZ,05L00ZZ,02VR0CZ \| \| 05L40ZZ,05L64ZZ,02LR3DT,02LR0DT,03L14ZZ \| \| 02LS4DZ,03L30ZZ,02VQ3CZ,05L43ZZ,03L04CZ \| \| 02VR4CZ,05L53ZZ,02VQ4CZ,05L14CZ,03L10DZ \| \| 02LR4DT,05L54CZ,05L10CZ,02LT4DZ,05L64DZ \| \| 05L50ZZ,03L40ZZ,03L24DZ,05L04DZ,05L10DZ \| \| 03L13ZZ,02LR3ZT,02LT3CZ,03L03DZ,05L60CZ \| \| 05L03DZ,05L63DZ \| |
| General | Procedures on spleen | total splenectomy | 41.5 | 07TP0ZZ |
|  | Gastrectomy | proximal gastrectomy | 43.5 | 0DB40ZZ |
|  |  | distal gastrectomy | 43.5 | \| 0DB43ZZ,0DB44ZZ,0DB47ZZ,0DT40ZZ,0DT44ZZ \| \| --- \| \| 0DT47ZZ,0DT48ZZ \| |
|  |  | part gastrec w jej anast | 43.7 | \| 0D160ZA,0D164ZA,0D168ZA,0DB60ZZ,0DB63ZZ \| \| --- \| \| 0DB64ZZ,0DB67ZZ,0DB68ZZ \| |
|  |  | partial gastrectomy nec | 43.89 | 0DB67ZZ |
|  |  |  | 43.89 | 0DB63ZZ, 0DB60ZZ |
|  |  | total gastrectomy nec | 43.99 | 0DT60ZZ,0DT67ZZ,0DT64ZZ,0DT68ZZ |
|  | Small bowel procedure | small bowel incision nec | 45.02 | \| 0D9B8ZZ,0D984ZZ,0DCB4ZZ,0D983ZZ,0D9B7ZZ \| \| --- \| \| 0D9830Z,0D9A7ZZ,0D9B4ZZ,0D9A4ZZ,0D9B00Z \| \| 0DC84ZZ,0D9800Z,0D9B40Z,0D9A30Z,0D9A8ZZ \| \| 0D9A3ZZ,0D9A00Z,0D9B0ZZ,0D987ZZ,0D9A0ZZ \| \| 0DC83ZZ,0D9B3ZZ,0D9B30Z,0D988ZZ,0D980ZZ \| \| 0DCA3ZZ,0DCA0ZZ,0DC80ZZ,0DCA4ZZ,0DCB0ZZ \| \| 0D9840Z,0D9A40Z,0DCB3ZZ \| |
|  |  | oth excise duodenum les | 45.31 | 0DB97ZZ,0DB90ZZ,0DB93ZZ |
|  |  | local excis sm bowel nec | 45.33 | 0DB80ZZ,0DB83ZZ,0DB88ZZ,0DB87ZZ,0DB84ZZ |
|  |  | mult seg sm bowel excis | 45.61 | 0DB87ZZ,0DB84ZZ,0DB80ZZ,0DB88ZZ,0DB83ZZ |
|  |  | part sm bowel resect nec | 45.62 | \| 0DT98ZZ,0DT90ZZ,0DTB8ZZ,0DTB0ZZ,0DT94ZZ \| \| --- \| \| 0DTA4ZZ,0DTB7ZZ,0DTA0ZZ,0DTA7ZZ,0DTB4ZZ \| \| 0DTA8ZZ,0DT97ZZ \| |
|  |  | sm-to-sm bowel anastom | 45.91 | \| 0D1A8ZA,0D1B8ZB,0D1A0ZB,0D1A8ZB,0D1B0ZB \| \| --- \| \| 0D1B4ZB,0D190ZB,0D194Z9,0D198ZA,0D198ZB \| \| 0D194ZB,0D190Z9,0D1A4ZA,0D1B8ZH,0D1A4ZB \| \| 0D194ZA,0D190ZA,0D1A8ZH,0D1A0ZA,0D198Z9 \| |
|  |  | sm bowel-rect stump anas | 45.92 | \| 0D1A0ZP,0D1H8ZP,0D1B0ZP,0D1B8ZP,0D1B4ZP \| \| --- \| \| 0D1A4ZP,0D1A8ZP \| |
|  |  | small-to-large bowel nec | 45.93 | \| 0D1A8ZN,0D1B4ZK,0D1A0ZH,0D1A0ZM,0D1A8ZM \| \| --- \| \| 0D1B8ZN,0D1A4ZL,0D1B8ZL,0D1A4ZK,0D1B4ZL \| \| 0D1B0ZK,0D194ZL,0D1B8ZM,0D190ZL,0D1B4ZM \| \| 0D1B4ZH,0D1B0ZL,0D1A8ZK,0D1B0ZN,0D1B0ZH \| \| 0D1B4ZN,0D1A8ZL,0D1A4ZN,0D1A4ZM,0D1B8ZK \| \| 0D198ZL,0D1A0ZK,0D1A0ZN,0D1A0ZL,0D1B0ZM \| \| 0D1A4ZH \| |
|  |  | anal anastomosis | 45.95 | 0D1B4ZQ, 0D1B8ZQ, 0D1B0ZQ |
|  |  | duodenal lacerat suture | 46.71 | 0DQ97ZZ,0DQ93ZZ,0DQ98ZZ,0DQ94ZZ,0DQ90ZZ |
|  |  | duodenal fistula closure | 46.72 | 0DQ94ZZ,0DQ90ZZ,0DQ93ZZ,0DQ97ZZ,0DQ98ZZ |
|  |  | close sm bowel fist nec | 46.74 | \| 0DQE4ZZ,0DQ83ZZ,0DQA8ZZ,0DQE3ZZ,0DQ87ZZ \| \| --- \| \| 0DQP7ZZ,0DQN3ZZ,0DQB3ZZ,0DQ83ZZ,0DQN7ZZ \| \| 0DQB0ZZ,0HQ7XZZ,0DQB4ZZ,0DQ88ZZ,0DQA7ZZ \| \| 0DQN8ZZ,0HQ6XZZ,0DQ88ZZ,0DQE7ZZ,0DQN4ZZ \| \| 0DQA3ZZ,0DQP0ZZ,0DQN0ZZ,0DQP3ZZ,0DQB0ZZ \| \| 0DQB3ZZ,0DQ87ZZ,0DQB7ZZ,0DQE8ZZ,0DQ84ZZ \| \| 0DQ80ZZ,0DQP8ZZ,0DQ80ZZ,0DQE0ZZ,0DQ84ZZ \| \| 0DQP4ZZ,0DQB7ZZ,0DQA4ZZ,0DQA0ZZ,0DQB4ZZ \| \| 0DQB8ZZ,0DQB8ZZ \| |
|  |  | repair of intestine nec | 46.79 | \| 0DQE0ZZ,0DQE8ZZ,0DQE4ZZ,0DQE7ZZ,0DQ93ZZ \| \| --- \| \| 0DQE3ZZ,0DQ90ZZ,0DQ94ZZ,0DQ98ZZ,0DQ97ZZ \| |
|  | Colorectal procedure | tot intra-abd colectomy# | 45.8 |  |
|  |  | abd-perineal rect resect# | 48.5 | 0DTP4ZZ,0DTP0ZZ,0D1N0Z4,0DTP7ZZ,0DTP8ZZ |
|  |  | opn mul seg lg intes nec | 45.71 | 0DBE7ZZ,0DBE8ZZ,0DBE0ZZ,0DBE3ZZ |
|  |  | open cecectomy nec | 45.72 | 0DTH7ZZ,0DTH8ZZ,0DTH0ZZ |
|  |  | opn rt hemicolectomy nec | 45.73 | 0DTF8ZZ,0DTF0ZZ,0DTK0ZZ,0DTF7ZZ |
|  |  | opn transv colon res nec | 45.74 | 0DTL8ZZ,0DTL0ZZ,0DTL7ZZ |
|  |  | opn lft hemicolectmy nec | 45.75 | 0DTG8ZZ,0DTG7ZZ,0DTG0ZZ |
|  |  | open sigmoidectomy nec | 45.76 | 0DTN8ZZ,0DTN7ZZ,0DTN0ZZ |
|  |  | prt lg intes exc nec/nos | 45.79 | 0DBE0ZZ,0DBE3ZZ,0DBE7ZZ,0DBE8ZZ |
|  |  | lg-to-lg bowel anastom | 45.94 | \| 0D1K4ZN,0D1H8ZL,0D1M8ZM,0D1N4ZN,0D1K8ZM \| \| --- \| \| 0D1M8ZN,0D1L0ZM,0D1H8ZM,0D1K0ZK,0D1M4ZN \| \| 0D1L4ZM,0D1M4ZM,0D1K0ZP,0D1K8ZL,0D1L8ZN \| \| 0D1K4ZL,0D1M4ZP,0D1M0ZM,0D1K0ZL,0D1L8ZM \| \| 0D1H4ZN,0D1H0ZH,0D1H4ZP,0D1H0ZM,0D1K4ZP \| \| 0D1H8ZN,0D1L0ZL,0D1H0ZP,0D1H8ZH,0D1L4ZN \| \| 0D1N0ZN,0D1K0ZM,0D1H8ZK,0D1L8ZL,0D1N0ZP \| \| 0D1H0ZL,0D1H4ZL,0D1M8ZP,0D1N4ZP,0D1L0ZN \| \| 0D1K4ZM,0D1H0ZK,0D1L4ZL,0D1H4ZH,0D1K0ZN \| \| 0D1H4ZK,0D1L4ZP,0D1K8ZN,0D1K4ZK,0D1K8ZK \| \| 0D1L8ZP,0D1L0ZP,0D1H4ZM,0D1M0ZN,0D1N8ZN \| \| 0D1H0ZN,0D1K8ZP,0D1M0ZP,0D1N8ZP \| |
|  |  | suture lg bowel lacerat | 46.75 | \| 0DQK3ZZ,0DQE7ZZ,0DQH8ZZ,0DQN4ZZ,0DQK8ZZ \| \| --- \| \| 0DQN0ZZ,0DQK4ZZ,0DQE4ZZ,0DQN3ZZ,0DQE8ZZ \| \| 0DQK7ZZ,0DQE3ZZ,0DQK0ZZ,0DQH0ZZ,0DQN8ZZ \| \| 0DQH4ZZ,0DQN7ZZ,0DQE0ZZ,0DQH7ZZ,0DQH3ZZ \| |
|  |  | revise lg bowel anastom | 46.94 | 0DQE3ZZ,0DQE4ZZ,0DQE0ZZ,0DQE7ZZ,0DQE8ZZ |
|  |  | ant rect resect w colost | 48.62 | 0DTP0ZZ,0DTP4ZZ,0D1N0Z4,0D1N4Z4 |
|  |  | anterior rect resect nec | 48.63 | 0DTP4ZZ, 0DTP0ZZ |
|  | Hernia repair (other than inguinal and femoral) | abd repair-diaphr hernia | 53.7 |  |
|  |  | thor rep-diaph hern nos | 53.8 | \| 0BQS0ZZ,0BQR3ZZ,0BQR4ZZ,0BQR0ZZ,0BQS4ZZ \| \| --- \| \| 0BQS3ZZ \| |
| Thoracic | Therapeutic esophageal procedures | esophageal incision nec | 42.09 | \| 0D954ZZ,0D9570Z,0D958ZZ,0D9540Z,0D953ZZ \| \| --- \| \| 0D950ZZ,0DC53ZZ,0D9530Z,0DC54ZZ,0DC50ZZ \| \| 0D957ZZ,0D9580Z \| |
|  |  | cervical esophagostomy | 42.11 | \| 0D114J4,0D11474,0D114Z4,0D113J4,0D11074 \| \| --- \| \| 0D114K4,0D110J4,0D110Z4,0D110K4 \| |
|  |  | esophagectomy nos | 42.4 | 0DB53ZZ,0DB50ZZ,0DB57ZZ |
|  |  | partial esophagectomy | 42.41 | 0DB57ZZ,0DB50ZZ,0DB53ZZ |
|  |  | total esophagectomy | 42.42 | 0DT50ZZ,0DT57ZZ,0DT54ZZ,0DT58ZZ |
|  |  | thorac esophagogastrost | 42.52 | \| 0D158Z6,0D158K6,0D158J6,0D154K6,0D15076 \| \| --- \| \| 0D154J6,0DX60Z5,0D150Z6,0DX64Z5,0D150K6 \| \| 0D150J6,0D154Z6,0D15876,0D15476 \| |
|  |  | suture esophageal lacer | 42.82 | 0DQ54ZZ,0DQ57ZZ,0DQ58ZZ,0DQ53ZZ,0DQ50ZZ |
|  |  | esophag stricture repair | 42.85 | \| 0D744ZZ,0D744DZ,0D754ZZ,0D740DZ,0D753DZ \| \| --- \| \| 0D754DZ,0D750ZZ,0D743ZZ,0D740ZZ,0D753ZZ \| \| 0D750DZ,0D743DZ \| |
|  |  | esophageal repair nec | 42.89 | 0DQ53ZZ,0DQ54ZZ,0DQ50ZZ,0DQ57ZZ,0DQ58ZZ |
| General | Therapeutic gastric procedure | gastroenterostomy nec | 44.39 | \| 0D160J9,0D160JA,0D160KA,0D160ZA,0D1687A \| \| --- \| \| 0D168K9,0D160Z9,0D1607A,0D168J9,0D160K9 \| \| 0D168ZA,0D168KA,0D16079,0D16879,0D168JA \| \| 0D168Z9 \| |
|  |  | sut gastric ulcer site | 44.41 | 0DQ60ZZ,0DQ63ZZ,0DQ64ZZ,0DQ67ZZ |
|  |  | suture duoden ulcer site | 44.42 | 0DQ98ZZ,0DQ97ZZ,0DQ94ZZ,0DQ93ZZ,0DQ90ZZ |
|  |  | suture gastric lacerat | 44.61 | 0DQ63ZZ,0DQ60ZZ,0DQ68ZZ,0DQ64ZZ,0DQ67ZZ |
|  |  | close gastric fistul nec | 44.63 | \| 0DQE3ZZ,0DQE4ZZ,0DQ83ZZ,0DQA4ZZ,0DQ68ZZ \| \| --- \| \| 0DQA3ZZ,0DQ84ZZ,0DQ63ZZ,0DQE8ZZ,0DQA7ZZ \| \| 0DQ64ZZ,0DQ60ZZ,0DQ67ZZ,0DQA8ZZ,0DQA0ZZ \| \| 0DQ87ZZ,0DQE0ZZ,0DQ80ZZ,0DQE7ZZ,0DQ88ZZ \| |
|  |  | esophagogastroplasty | 44.65 | 0DQ40ZZ,0DQ44ZZ,0DQ43ZZ,0DQ48ZZ,0DQ47ZZ |
|  |  | gastric repair nec | 44.69 | \| 0DV60DZ,0DV63DZ,0DV60ZZ,0DV64ZZ,0DV67ZZ \| \| --- \| \| 0DV63CZ,0DQ63ZZ,0DV63ZZ,0DQ60ZZ,0DV64DZ \| \| 0DV60CZ,0DQ68ZZ,0DQ67ZZ,0DV68ZZ \| |
|  | Biliary major | gb-to-intestine anastom | 51.32 | \| 0F144ZB,0F140Z3,0F140ZB,0F144Z3,0F144DB \| \| --- \| \| 0F144D3,0F140DB,0F140D3 \| |
|  |  | choledochoenterostomy | 51.36 | 0F194Z3,0F190D3,0F194D3,0F190Z3 |
|  |  | hepatic duct-gi anastom | 51.37 | \| 0F194ZB,0F154D3,0F150Z3,0F154Z3,0F160DB \| \| --- \| \| 0F164ZB,0F164DB,0F150ZB,0F184DB,0F164Z3 \| \| 0F184ZB,0F164D3,0F150D3,0F194DB,0F184D3 \| \| 0F154ZB,0F160ZB,0F150DB,0F160Z3,0F180DB \| \| 0F190DB,0F190ZB,0F180D3,0F180ZB,0F180Z3 \| \| 0F184Z3,0F154DB,0F160D3 \| |
|  |  | bile duct repair nec | 51.79 | \| 0FQ63ZZ,0FQ68ZZ,0FQ84ZZ,0FQ87ZZ,0FQ53ZZ \| \| --- \| \| 0FQ57ZZ,0FQ60ZZ,0FQ54ZZ,0FQ58ZZ,0FQ83ZZ \| \| 0FQ80ZZ,0FQ67ZZ,0FQ50ZZ,0FQ88ZZ,0FQ64ZZ \| |
|  | Pancreatic procedure | pancreat cyst marsupiali | 52.3 | 0F9G0ZZ,0F9G3ZZ,0F9G4ZZ |
|  |  | int drain pancreat cyst | 52.4 | \| 0F1D0D3,0F1D0DB,0F1D0Z3,0F1D0ZB,0F1D4D3 \| \| --- \| \| 0F1D4DB,0F1D4Z3,0F1D4ZB \| |
|  |  | total pancreatectomy | 52.6 | \| 0DT90ZZ,0DT94ZZ,0DT97ZZ,0DT98ZZ,0FTG0ZZ \| \| --- \| \| 0FTG4ZZ \| |
|  |  | rad pancreaticoduodenect | 52.7 | \| 0D1607A,0D160JA,0D160KA,0D160ZA,0DT90ZZ \| \| --- \| \| 0DT90ZZ,0F190Z3,0F1G0ZC,0FTG0ZZ,0FTG0ZZ \| |
|  |  | pancreatotomy nec | 52.09 | \| 0FFD4ZZ,0FCG0ZZ,0FCD7ZZ,0FFD3ZZ,0FFD0ZZ \| \| --- \| \| 0F9G3ZZ,0F9D4ZZ,0F9D3ZZ,0F9D8ZZ,0F9D0ZZ \| \| 0FCG4ZZ,0FCG3ZZ,0FFD8ZZ,0F9G4ZZ,0FFD7ZZ \| \| 0F9G0ZZ,0F9D7ZZ,0FCD0ZZ \| |
|  |  | other destru pancrea les | 52.22 | \| 0FTD0ZZ,0F5D7ZZ,0FBD0ZZ,0FTD7ZZ,0F5D0ZZ \| \| --- \| \| 0F5G3ZZ,0FBG0ZZ,0F5G0ZZ,0F5D3ZZ,0FBD3ZZ \| \| 0FBD7ZZ,0FBG3ZZ \| |
|  |  | distal pancreatectomy | 52.52 | 0FBG4ZZ,0FBG0ZZ,0FBG3ZZ |
|  |  | rad subtot pancreatectom | 52.53 | 0FBG4ZZ,0FBG0ZZ,0FBG3ZZ |
|  |  | partial pancreatect nec | 52.59 | 0FBG4ZZ,0FBG3ZZ,0FBG0ZZ |
|  |  | pancreatic anastomosis | 52.96 | \| 0F1G0D3,0F1D0D3,0F1G4ZB,0F1G4D3,0F1G4DB \| \| --- \| \| 0F1D4DB,0F1G0ZB,0F1D4ZB,0F1D4D3,0F1D0Z3 \| \| 0F1D0ZB,0F1D0DB,0F1G4Z3,0F1D4Z3,0F1G0DB \| \| 0F1G0Z3 \| |
|  | Liver major | hepatic lobectomy | 50.3 | 0FT10ZZ,0FT14ZZ,0FT20ZZ,0FT24ZZ |
|  |  | partial hepatectomy | 50.22 | 0FB00ZZ,0FB04ZZ,0FB03ZZ |
| Urology | Nephrectomy | nephroureterectomy | 55.51 | \| 0TT00ZZ,0TT04ZZ,0TT14ZZ,0TT10ZZ,0TT68ZZ \| \| --- \| \| 0TT67ZZ,0TT70ZZ,0TT64ZZ,0TT78ZZ,0TT74ZZ \| \| 0TT04ZZ,0TT14ZZ,0TT60ZZ,0TT00ZZ,0TT77ZZ \| \| 0TT10ZZ \| |
|  |  | bilateral nephrectomy | 55.54 | 0TT24ZZ, 0TT20ZZ |
|  | Major therapeutic procedures of urinary tract | form cutan ileoureterost | 56.51 | \| 0T164ZC,0T1707C,0T170JC,0T1747C,0T184JC \| \| --- \| \| 0DBB0ZZ,0T184KC,0T180KC,0T174KC,0T164KC \| \| 0DBB4ZZ,0T160KC,0T174ZC,0T174JC,0T1847C \| \| 0DBB7ZZ,0T164JC,0T184ZC,0T160KC,0T1607C \| \| 0T170ZC,0T1647C,0T184KC,0T160JC,0T160ZC \| \| 0T1807C,0T1847C,0DBB8ZZ,0T170KC,0T174KC \| \| 0T164KC,0T1807C,0T180ZC,0DBB3ZZ,0T1747C \| \| 0T180KC,0T1607C,0T170KC,0T1707C,0T1647C \| \| 0T180JC \| |
|  |  | radical cystectomy | 57.71 | \| 0VT30ZZ,0TTB4ZZ,0TTD8ZZ,0TTB0ZZ,0TTD7ZZ \| \| --- \| \| 0VT07ZZ,0VT34ZZ,0TTB8ZZ,0TTB7ZZ,0VT08ZZ \| \| 0VT04ZZ,0VT00ZZ,0TTD0ZZ,0TTB4ZZ,0TTB7ZZ \| \| 0TTB8ZZ,0TTD4ZZ,0TTB0ZZ \| |
|  |  | suture bladder lacerat | 57.81 | 0TQB8ZZ,0TQB3ZZ,0TQB0ZZ,0TQB4ZZ,0TQB7ZZ |
|  |  | enterovesico fist repair | 57.83 | \| 0DQP3ZZ,0DQN0ZZ,0TQB7ZZ,0DQP7ZZ,0DQP0ZZ \| \| --- \| \| 0DQE0ZZ,0TQB8ZZ,0UQG8ZZ,0DQP8ZZ,0TQD7ZZ \| \| 0DQB3ZZ,0DQP4ZZ,0DQB4ZZ,0TQB4ZZ,0UQG4ZZ \| \| 0DQN0ZZ,0DQP8ZZ,0UQG7ZZ,0DQB0ZZ,0DQP4ZZ \| \| 0TQB7ZZ,0TQB8ZZ,0DQP7ZZ,0DQN8ZZ,0DQN7ZZ \| \| 0DQN4ZZ,0TQB0ZZ,0DQ87ZZ,0TQB4ZZ,0TQB8ZZ \| \| 0DQE8ZZ,0TQB8ZZ,0DQN3ZZ,0DQN7ZZ,0DQE3ZZ \| \| 0DQB7ZZ,0TQB3ZZ,0UQG3ZZ,0DQN4ZZ,0TQB7ZZ \| \| 0DQP3ZZ,0TQD3ZZ,0DQN3ZZ,0DQP0ZZ,0DQE4ZZ \| \| 0TQB4ZZ,0DQP3ZZ,0DQB8ZZ,0UQG0ZZ,0DQN8ZZ \| \| 0TQB3ZZ,0TQB3ZZ,0DQP8ZZ,0TQD8ZZ,0TQD0ZZ \| \| 0TQB0ZZ,0TQB7ZZ,0DQE7ZZ,0DQP0ZZ,0TQB0ZZ \| \| 0DQ88ZZ,0TQD4ZZ,0DQP4ZZ,0DQP7ZZ,0TQB4ZZ \| \| 0TQB0ZZ,0DQ80ZZ,0DQ84ZZ,0TQB3ZZ,0DQ83ZZ \| |
|  |  | perivesical incision nec | 59.19 | 0WJJ0ZZ,0TJB0ZZ,0WJJ3ZZ,0TJB4ZZ,0WJJ4ZZ |
| Gynecology | Major theraptueic procedures; female organs | pelvic evisceration | 68.8 | \| 0DTN0ZZ,0DTP0ZZ,0TTB0ZZ,0TTB0ZZ,0TTD0ZZ \| \| --- \| \| 0TTD0ZZ,0UT20ZZ,0UT20ZZ,0UT70ZZ,0UT70ZZ \| \| 0UT90ZZ,0UT90ZZ,0UTC0ZZ,0UTC0ZZ,0UTG0ZZ \| \| 0UTG0ZZ \| |
| Orthopedics | Amputation of lower extremity | disarticulation of hip | 84.18 | 0Y680ZZ,S53499A,0Y670ZZ |
|  |  | hindquarter amputation | 84.19 | 0Y620ZZ,S56919A,0Y640ZZ,0Y630ZZ,S53409A |
| Transplant | Ot orgn tran | heart transplantation# | 37.5 |  |
|  |  | heart transplantation | 37.51 | 02YA0Z2,02YA0Z0,02YA0Z1 |
|  |  | liver transplant nec | 50.59 | 0FY00Z0,0FY00Z1,0FY00Z2 |

Abbreviations: ICD-9/10: International Classification of Diseases, Ninth/Tenth; CM: Clinical Modification; PCS: Procedure Coding System

**Appendix Table 2.** Frailty measures from the 10-Item Johns Hopkins ACG with associated conditions and related ICD-9/10-CM codes

| **Diagnosis** | **ICD-9-CM Codes** | **ICD-10-CM Codes** |
| --- | --- | --- |
| Malnutrition | 261, 262, 263.8, 263.9, V77.2 | E41, E43, E44.0, E44.1, E45, E46,  Z13.21 |
| Senile dementia with  delusional or depressive  feature or with delirium | 290.20, 290.21, 290.3 | F03.90, F05 |
| Severe vision impairment | 369.0, 369.00, 369.01, 369.03,  369.04, 369.06, 369.07, 369.08,  369.1, 369.10, 369.16, 396.18,  396.12, 369.14 | H54.0, H54.10 |
| Decubitus ulcer | 707.0, 707.00, 707.01, 707.02, 707.03, 707.04, 707.05, 707.06, 707.07, 707.09, 707.20, 707.21, 707.22, 707.23, 707.24, 707.25 | L89.119, L89.129, L89.139, L89.149,  L89.159, L89.209, L89.309, L89.509,  L89.609, L89.899, L89.90 |
| Incontinence of urine | 788.34, 788.37 | N39.42, N39.45 |
| Loss of weight | 783.2, 783.21, 783.22, 783.3 | R63.3, R63.4, R63.6 |
| Fecal incontinence | 787.6, 787.60 | R15.9 |
| Lack of housing  (Social support needs) | V60.0, V60.1, V60.2 | Z59.0, Z59.1, Z59.5 |
| Difficulty in walking | 719.7, 781.2 | R26.1, R26.2, R26.9 |
| Fall on stairs or steps, or  from wheelchair | E880, E880.0, E880.1, E880.9, E884.3 | W05.2XXA, W10.0XXA, W10.1XXA,  W10.8XXA |

Abbreviations: ACG, Adjusted Clinical Groups (ACG); ICD, International Classification of Diseases; CM, Clinical Modification

**Appendix Table 3.** Estimates of the Association between race and ethnicity and the receipt of inpatient palliative care consultation from the Logistic Regression Model

|  | Covariate-Adjusted Probabilities of  receiving inpatient palliative care consultation (%)  from  Logistic Regression Model (95% CI) |
| --- | --- |
| **Difference among non-frail patients** |  |
| Black – Asian/Pacific Islander | 0.06 (-0.09, 0.21) |
| Black – Hispanic/Latine | 0.02 (-0.11, 0.14) |
| Black – Other | 0.16 (0.02, 0.29) |
| Black – White | 0.05 (-0.04, 0.13) |
| Asian/Pacific Islander – Hispanic/Latine | -0.04 (-0.19, 0.11) |
| Asian/Pacific Islander – Other | 0.10 (-0.06, 0.26) |
| Asian/Pacific Islander – White | -0.01 (-0.14, 0.12) |
| Hispanic/Latine – Other | 0.14 (-0.00, 0.28) |
| Hispanic/Latine – White | 0.03 (-0.07, 0.13) |
| Other – White | -0.11 (-0.22, 0.00) |
| **Difference among frail patient** |  |
| Black – Asian/Pacific Islander* | -0.99 (-1.66, -0.32) |
| Black – Hispanic/Latine * | -0.45 (-0.89, -0.02) |
| Black – Other | -0.34 (-0.89, 0.20) |
| Black – White* | -0.61 (-0.86, -0.37) |
| Asian/Pacific Islander – Hispanic/Latine | 0.53 (-0.18, 1.25) |
| Asian/Pacific Islander – Other | 0.64 (-0.16, 1.45) |
| Asian/Pacific Islander – White | 0.38 (-0.26, 1.01) |
| Hispanic/Latine – Other | 0.11 (-0.47, 0.69) |
| Hispanic/Latine – White | -0.16 (-0.54, 0.22) |
| Other – White | -0.27 (-0.77, 0.23) |

**Appendix Figure 1.** Boxplots of standardized mean differences of covariates between racial/ethnic groups


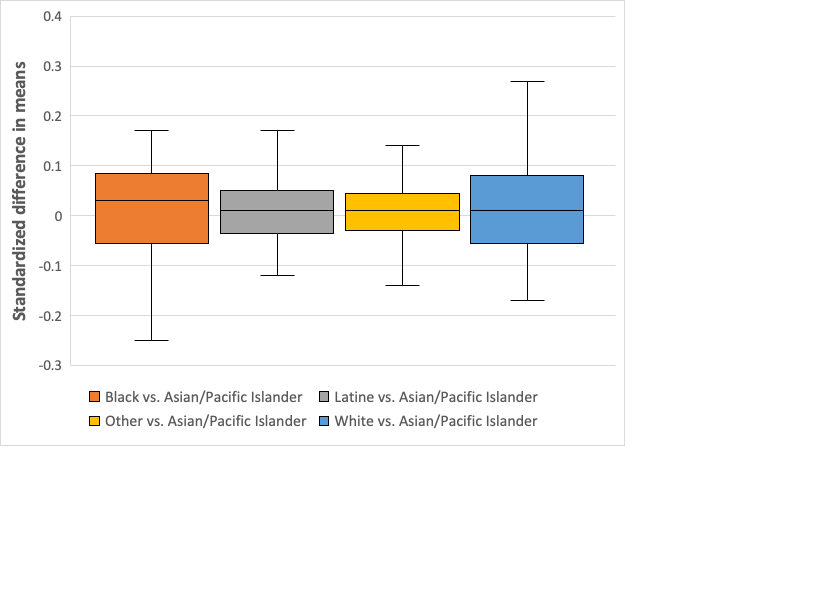


**Note:** Standardized differences in means greater than 0.1 or 0.2 represent a substantial difference. The error bars represent the upper and lower extreme values, but some of them are very small in scale.
